# High-Pressure Limit and Pressure-Dependent Rate Rules for *β*-Scission Reaction Class of Hydroperoxyl Alkyl Hydroperoxyl Radicals (•P(OOH)_2_) in Normal-Alkyl Cyclohexanes Combustion

**DOI:** 10.3390/molecules29020544

**Published:** 2024-01-22

**Authors:** Xiaoxia Yao, Xiaoli Sun, Yifei Zhu

**Affiliations:** 1 National Key Lab of Aerospace Power System and Plasma Technology, Air Force Engineering University, Xi’an 710038, China; yaoxxkgd@163.com; 2 Institute of Theoretical Chemistry, Jilin University, Changchun 130023, China; 3 School of Mechanical Engineering, Xi’an Jiaotong University, Xi’an 710049, China

**Keywords:** hydroperoxyl alkyl hydroperoxyl radicals, *β*-scission reaction class, reaction rate constants, reaction rate rules

## Abstract

Chemical kinetic studies of the *β*-scission reaction class of hydroperoxyl alkyl hydroperoxyl radicals (•P(OOH)_2_) from normal-alkyl cyclohexanes are carried out systematically through high-level ab initio calculations. Geometry optimizations and frequency calculations for all species involved in the reactions are performed at the B3LYP/CBSB7 level of theory. Electronic single-point energy calculations are calculated at the CBS-QB3 level of theory. Rate constants for the reactions of *β*-scission, in the temperature range of 500–1500 K and the pressure range of 0.01–100 atm, are calculated using transition state theory (TST) and Rice-Ramsberger-Kassel-Marcus/Master-Equation (RRKM/ME) theory taking asymmetric Eckart tunneling corrections and the one-dimensional hindered rotor approximation into consideration. The rate rules are obtained by averaging the rate constants of the representative reactions of this class. These rate rules can greatly assist in constructing more accurate low-temperature combustion mechanisms for normal-alkyl cyclohexanes.

## 1. Introduction

Hydrocarbons can be classified into several major groups, including alkanes, cycloalkanes (also known as naphthenes), aromatics, and alkenes [1]. Cycloalkanes, especially those with C5 and C6 rings, are significant constituents of traditional transportation fuels like diesel and gasoline, accounting for approximately 10 to 30% of their composition [2,3]. They also play a crucial role in jet fuels such as Jet-A/Jet-A1/JP-8 and RP-1, making up a considerable portion of their composition [4,5,6,7]. Based on a comprehensive global study, it has been estimated that mono-, di-, and tri-cycloalkanes collectively constitute approximately 21% of the composition of jet fuel [8]. Recently, there has been growing interest in cyclohexane and related compounds due to the impending depletion of conventional petroleum sources and the ever-increasing prices of fuels. Compared with non-cyclic counterparts such as alkanes, cycloalkanes are more prone to dehydrogenation due to their ring structure, resulting in the formation of unsaturated compounds such as cycloalkenes. These cycloalkenes can be further aromatized to form aromatics. Compared with non-aromatic compounds, aromatic compounds have a higher tendency to form soot during combustion. In addition, the existence of cyclic structures in cycloalkanes is conducive to the formation of polycyclic aromatic hydrocarbons (PAHs). PAHs are large, complex molecules made up of multiple carbon rings fused together. They are highly thermally stable and are considered to be significant contributors to soot emissions [9,10,11]. To mitigate the production of unwanted pollutants and enhance the practical performance of engines, it is crucial to develop detailed combustion models that better represent low-temperature combustion techniques. These models play a crucial role in identifying critical reaction pathways and comprehending the low-temperature oxidation characteristics of large hydrocarbon fuels under different temperature and pressure conditions. This understanding is of immense significance in illuminating ignition phenomena within practical combustion systems like HCCI engines, jet engines, and spark ignition systems [12,13,14,15,16,17].

In the aspects of theoretical research, cycloalkanes [18,19,20,21] have received more attention in the past decade because they have the simplest molecular structure among all of the cyclic alkanes with a six-membered ring. The study of the oxidation reactivity of cyclohexanes with substituted alkyl-chains continues to be neglected, and very little information is available. There are only a few studies of the oxidation of methyl cyclohexane, ethyl cyclohexane, n-propyl cyclohexane, and n-butyl cyclohexane. In their study, Yang et al. [22] employed quantum chemical calculations, specifically CBS-QB3, to examine the uniqueness of the low-temperature oxidation of cycloalkanes. Their research focused on the influence of methyl substitution and analyzed the impacts of molecular structure, particularly in relation to potential energy surfaces (PES) involved in the isomerization reactions of ROO•, specifically 1,4 and 1,5 H-migration of cyclic alkylperoxyl radicals. Weber et al. [23] conducted calculations to determine the isomerization rate constants for the 2-methylcyclohexyl-1-peroxy radical, where the -OO group is attached at the beta position in relation to the methyl group. The researchers employed the CBS-QB3 composite method to calculate high-pressure limit rate constants, which were then utilized to update the kinetic model. Xing et al. [24,25] studied three different types of methyl cyclohexyl radicals, focusing on the low-temperature combustion reactions of these radicals. Furthermore, their work delved into the similarities and differences in reaction kinetics between acyclic and cyclic alkanes. Ning et al. [26] combined electron structure theory, transition state theory (TST), and Rice-Ramsberger-Kassel-Marcus/Master-Equation (RRKM/ME) theory to study the oxidation mechanism and kinetics of ethyl cyclohexane at low and medium temperatures. The high-pressure limit rate constants of tight transition state reactions are obtained from TST, and the high-pressure limit rate constants of barrierless reactions are obtained from the variational transition state theory (VTST). The rate constants for pressure-dependent reactions within the fall-off range were obtained using RRKM/ME theory. Through accurate computational analysis, valuable insights into the behavior of these reactions under varying temperature and pressure conditions were gained, thereby enhancing the low-temperature combustion model for cyclic alkanes. Liu et al. [27] conducted comprehensive chemical kinetic studies using high-level ab initio calculations to investigate hydrogen atom abstraction reactions by hydroperoxyl radicals from six alkyl cyclohexanes. Their systematic approach involves performing geometry optimizations and frequency calculations for all species involved in the reactions at the M06-2X/6-311++G(d,p) level of theory. Their approach also includes electronic single-point energy calculations, incorporating zero-point energy corrections, conducted at the UCCSD(T)-F12a/cc-pVDZ-F12 level of theory. They used the obtained kinetic and thermochemical data to update the mechanism of alkyl cyclohexane, and carried out further analysis to determine the impact of ignition delay time (in shock tube and fast compressor experiments) on the prediction. In our previous studies, we conducted systematic investigations into the isomerization reaction class of peroxyl alkyl radicals, the concerted elimination reaction class of peroxyl alkyl radicals, the *β*-scission reaction class of hydroperoxyl alkyl radicals, and the concerted elimination reaction class of peroxyl hydroperoxyl alkyl radicals derived from methyl, ethyl, n-propyl, and n-butyl cyclohexane. Through these studies, we developed high-pressure limit rate rules and pressure-dependent rate rules for these reactions. Ali et al. [28] examined the reactivity of n-butyl cyclohexane by employing the high-level quantum composite G3B3 method in conjunction with RRKM/ME simulations to elucidate reaction pathways. They obtained precise fuel ring-opening rate constants under high temperature conditions and extensively investigated seven distinct unimolecular decomposition channels for ring-opening. However, their study lacked theoretical kinetics and quantum chemical calculations at lower temperatures. Yang et al. [3] conducted a theoretical study on the H-migration reactions of cyclic alkylperoxy radicals and calculated the high-pressure limit rate constant and pressure-dependent rate constant of different reactions. All reactions are classified into seven reaction types, and these rules may be used in the development of low-temperature kinetic mechanisms for cycloalkanes. Generally speaking, as the simplest normal-alkyl cyclohexane, methyl cyclohexane has been widely studied in recent years. In contrast with methyl cyclohexane, the study on the combustion behavior of cyclohexanes with larger substituted alkyl-chains (the alkylic side chains from C2 to C4) remains too limited, and very little information is available. Therefore, increasing the credibility of a kinetic mechanism in jet fuels deserves much more effort, not only in experimental work but also in theoretical calculations.

**Scheme 1:**
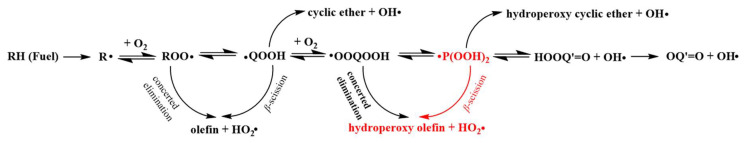
The kinetic scheme for the low-temperature oxidation of cycloalkanes, with the specific class of reaction studied in this research highlighted in red [30].

The simplified kinetic scheme for the low-temperature oxidation of cycloalkanes, which is similar to alkanes, is illustrated in Scheme 1 [29].

Initially, alkane consumption takes place through H-atom abstraction, resulting in the formation of alkyl radicals (R•). These R• can react with the first molecular oxygen to generate alkyl peroxy radicals (ROO•), with the possibility of intermolecular hydrogen migration leading to the formation of hydroperoxyl alkyl radicals (•QOOH). Subsequently, •QOOH can react with the second molecular oxygen to produce hydroperoxylalkyl peroxyl radicals (•OOQOOH). Through intramolecular hydrogen migration, •OOQOOH can further transform into hydroperoxyl alkyl hydroperoxyl radicals (•P(OOH)_2_). In the low temperature regions, the decomposition of a •P(OOH)_2_ with a radical site beta to the -OOH moiety can result in the production of high molecular weight hydroperoxy olefin and HO_2_ radicals. This type of reaction is thought to be one of the reasons for the negative temperature coefficient (NTC) behavior of the fuel, as the reaction generates a relatively inactive HO_2_ radical, which has the potential to inhibit the ignition behavior of normal-alkyl cyclohexanes.

With the continuous advancement of engine technology and the introduction of new fuels, it remains crucial to effectively anticipate combustion behavior under various conditions of temperature, pressure, and equivalence ratio. This requires reliance on dependable combustion mechanisms and models that can accurately predict such behavior. The reliability of these combustion mechanisms depends on the comprehensiveness of the combustion reaction network as well as the precision of thermodynamic and kinetic parameters. However, precise kinetic parameters for various important reaction classes are difficult to obtain through experimental methods during the combustion process. Therefore, it becomes necessary to rely on advanced theoretical calculation methods to obtain rate constants for these crucial reactions. Internationally, numerous studies have been conducted to explore the formation of cyclohexenes and HO_2_ radicals through *β*-scission reactions of •QOOH derived from specific cycloalkanes. For instance, Xing et al. [24,25] focused on methyl cyclohexane and investigated potential energy surfaces, calculating pressure-dependent rate constants for the *β*-scission of •QOOH using the theoretical QCISD(T)/CBS level. Similarly, Ning et al. [26] examined the oxidation mechanisms and kinetics of ethyl cyclohexane at low and intermediate temperatures, and computed pressure-dependent rate constants for the *β*-scission of •QOOH using the CBS-QB3 level. In our previous work, we extensively investigated the potential energy surface of the oxidation pathway for the *β*-scission reactions of •QOOH in cyclohexanes during low-temperature combustion. Moreover, we accurately calculated the rate constants for this reaction class using the highly reliable CBS-QB3 theoretical level. Our findings strongly indicate that the *β*-scission reactions of •QOOH play a significant role in the oxidation pathways of cyclohexanes at low temperatures. However, research on the kinetics of the *β*-scission reactions of •P(OOH)_2_ in cycloalkanes has been relatively limited. Consequently, in modeling studies of cycloalkane combustion, certain kinetic parameters are estimated by assuming similarities with reaction classes in alkanes. These approximations can introduce substantial errors in the modeling process.

Constructing a comprehensive combustion mechanism involves thousands of elementary reactions and hundreds of species. As hydrocarbon molecules increase in carbon atom count, the diversity and quantity of elementary reactions escalate exponentially, rendering manual construction of a detailed combustion mechanism impossible. A detailed hydrocarbon fuel mechanism typically comprises two primary components. First, a core mechanism encompasses smaller species (C0–C4) exhibiting high generality. Second, an extended mechanism caters to larger molecules (C5 and beyond) and is automatically generated by mechanism generation programs employing reaction class rules [31]. This approach ensures an efficient and accurate representation of complex combustion processes. To the best of our knowledge, there is currently no existing literature that discusses rate rules for the essential *β*-scission process from •P(OOH)_2_ of cyclohexanes. This process plays a critical role in constructing accurate low- and intermediate-temperature models for cycloalkanes.

Hence, the objective of this study is to comprehensively investigate the *β*-scission reactions of cyclohexanes to bridge the research gap in this field. Through theoretical calculations, we determine the rate constants at the high-pressure limit and a number of other pressures. Ultimately, this research aims to establish accurate high-pressure limit rate rules and the pressure-dependent rate rules for *β*-scission reactions of hydroperoxyl alkyl hydroperoxyl radicals in normal-alkyl cyclohexanes. These findings will serve as a theoretical foundation for constructing more comprehensive low-temperature combustion mechanisms for normal-alkyl cyclohexanes.

## 2. Results and Discussion

**Scheme 2:**
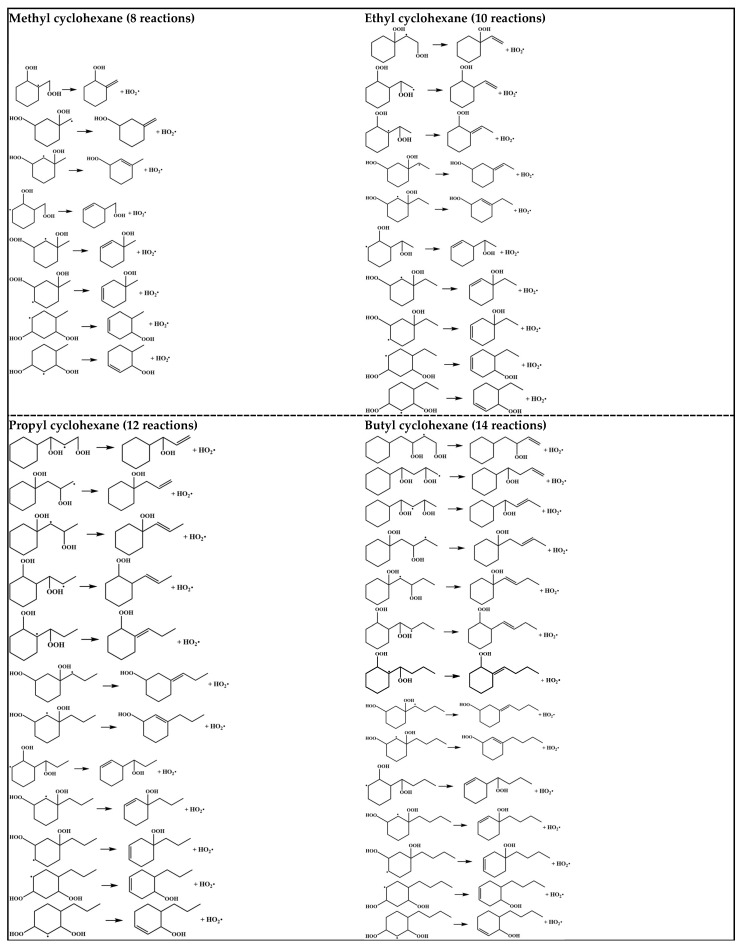
Summary sheet of the reactions for *β*-scission reaction of •P(OOH)_2_.

In our previous study, it was revealed that •QOOH generated via the 1.5 H-migration of ROO• from normal-alkyl cyclohexane is the primary intermediate in the H-migration reaction pathway, and this •QOOH species further facilitates O_2_ addition. Consequently, the initial reactant configuration selected for the *β*-scission reaction of •P(OOH)_2_ corresponding to the •QOOH configuration resulting from the 1.5 H-migration of ROO•. For our present investigation, a comprehensive set of 44 representative reactions involving methyl, ethyl, n-propyl, and n-butyl cyclohexane was chosen. These selected reactions are displayed in Scheme 2.

### 2.1. Conformational Analysis

**Scheme 3:**
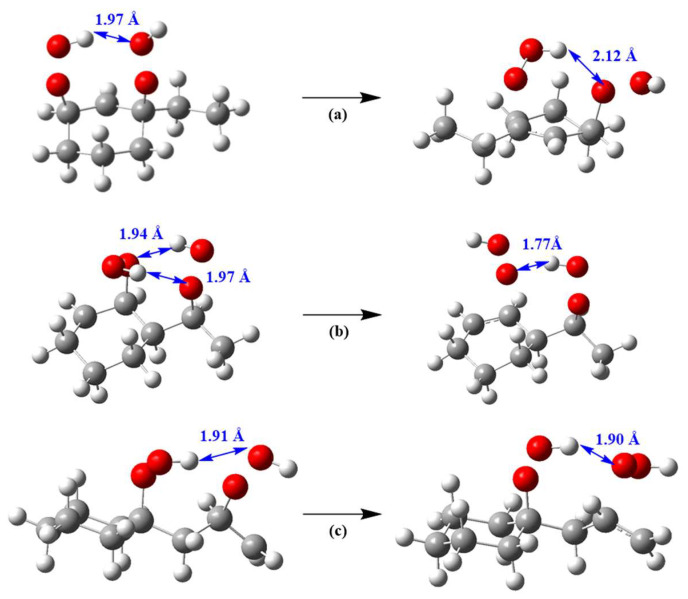
The presence of hydrogen bonds in reactants (left) and transition state (right) for three different scenarios: (**a**) where both -OOH groups are positioned on the ring; (**b**) where one -OOH group is situated on the ring and one on the alkyl side chain; (**c**) where both -OOH groups are placed on the alkyl side chain.

In this study system, the significance of intramolecular hydrogen bonds between -OOH and -OOH groups in the reactants and transition states emphasizes the need to investigate their impact on conformational variations. Specifically, this study focuses on how intramolecular hydrogen bonds formed between the hydrogen atoms in one -OOH group and the oxygen atoms in another -OOH group affect the conformational behavior of the species. The influence of hydrogen bonding on the formation of ring structures plays a crucial role in diverse conformations and ring-to-ring conversions. This study focuses on three specific scenarios regarding the placement of -OOH and -OOH groups within a cycloalkane: (1) both groups located on the ring (Scheme 3a); (2) one group on the ring and the other on the alkyl side chain (Scheme 3b); (3) both groups positioned on the alkyl side chain (Scheme 3c).

In order to demonstrate the significance of intramolecular hydrogen bonding, we have chosen the reaction R17 as a representative example. Our aim is to study and analyze the effects of the presence or absence of intramolecular hydrogen bonding on the configurations of the reactants and the transition states, and further on the energy barriers associated with reactions, and the results are shown in Table 1. In Case (1), where neither the reactants nor the transition states contain intramolecular hydrogen bonds, the energy barrier is measured at 16.4 kcal mol^−1^. Contrarily, in Case (2) of Table 1, the presence of intramolecular hydrogen bonds in the reactant leads to a reduction in energy, resulting in a higher energy barrier of 19.9 kcal mol^−1^. However, in Case (3), we find that the presence of intramolecular hydrogen bonds in the transition state leads to a decrease in energy, resulting in a lower energy barrier of 12.7 kcal mol^−1^. Finally, in Case (4), both the reactant and transition state contain intramolecular hydrogen bonds, and the energies of both the reactant and transition state decrease, resulting in a lower energy barrier of 16.2 kcal mol^−1^ compared to Case (1) in Table 1. However, an analysis of the intrinsic reaction coordinates (IRCs) for the four reactions listed in Table 1 reveals that only the reactant structure containing an intramolecular hydrogen bond can connect with the transition state that also contains an intramolecular hydrogen bond. Conversely, the reactant structure without an intramolecular hydrogen bond can only connect with a transition state that lacks an intramolecular hydrogen bond. As a result, only reactions (1) and (4) from Table 1 are observed in practical scenarios. Furthermore, due to the significant reduction in energy barriers offered by intramolecular hydrogen bonds, this study considers the presence of intramolecular hydrogen bonds in both the reactant and transition state structures.

### 2.2. Energy Barrier

In this study, the reaction barriers for all *β*-scission reactions of •P(OOH)_2_ are calculated using the CBS-QB3 level and are documented in Table 2. At the same time, we calculate the average energy barrier and the maximum energy barrier deviation of the 44 reactions in this study, where the maximum energy barrier deviation represents the difference between the highest and lowest barriers, which are also listed in Table 2. It can be seen from Table 2 that the maximum energy barrier deviation is 2.5 kcal mol^−1^, which indicates that for the *β*-scission reactions of •P(OOH)_2_, the difference in energy barriers of the 44 representative reactions selected is not particularly large. Therefore, a general reaction rate rule can be constructed for this reaction class.

### 2.3. Comparison of Energy Barriers for β-Scission Class

In our previous studies, we have conducted systematic investigations into the *β*-scission reaction class of •QOOH derived from methyl, ethyl, n-propyl, and n-butyl cyclohexane, and we have computed the energy barriers for the *β*-scission reactions of •QOOH utilizing the CBS-QB3 method. In order to verify whether intramolecular hydrogen bonds resulting from the presence of two -OOH groups in •P(OOH)_2_ affect the energy barriers of the *β*-scission reactions of •P(OOH)_2_, we compared those with the energy barriers of the *β*-scission reactions of •QOOH (reactive species containing one -OOH group). The results are shown in Table 3, and the difference in the energy barriers between two classes is discussed.

As can be seen from Table 3, when the radical sites are located on the primary and secondary carbon, the energy barriers of the *β*-scission reactions of •QOOH is basically similar to or lower than those of •P(OOH)_2_. When the radical sites are located on the tertiary carbon, the energy barriers of the *β*-scission reactions of •QOOH are higher than those of •P(OOH)_2_. However, when the reaction centers of the *β*-scission reactions occur on the ring, there are some *β*-scission reactions of •QOOH that have lower energy barriers than those of •P(OOH)_2_, while others have higher energy barriers than those of •P(OOH)_2_. These comparison results show that the presence of intramolecular hydrogen bonds in the reactants and transition states will affect the energy barrier of the reactions with similar reaction classes.

### 2.4. Rate Constants and Rate Rules

In this study, rate constants for the investigated reactions are calculated within the temperature range of 500 K to 1500 K. These rate constants are then fitted to the modified Arrhenius Equation:

(1) $$k\left(T\right)=AT^{n}exp\left[-E_{a}/RT\right]$$

Which is given in the form (*A*, *n*, *E*). To derive the rate rule for a class, the rate constant of each reaction at each temperature is fitted to Equation (2):

(2) $$k_{avg}^{T}=\frac{1}{N}\sum_{i=1}^{i=N}k_{i}^{T}$$

where $k_{avg}^{T}$ denotes the average rate constant at temperature *T*, the rate constant for the *i* reaction at temperature *T* is denoted by $k_{i}^{T}$, and *N* represents the total number of reactions within a particular class. By utilizing this method, a three-parameter (*A*, *n*, *E*) representation for the rate rule of the class is obtained through fitting to the modified Arrhenius Equation.

To measure the uncertainty and the applicability of the rate rule for a class, an uncertainty factor f of a reaction class is defined as: *f* = *k_max_*/*k_min_*, where *k_max_* and *k_min_* are the maximum and the minimum rate constant in a class, respectively. *k*/*k_ave_*, which is defined as the ratio of the rate constant of a reaction to the average rate constant of the reactions in a class, are also calculated at a given temperature to measure the suitability of the rate rule.

#### 2.4.1. High-Pressure Limit Rate Constants and Rate Rules

The calculated high-pressure limit rate constants at temperatures from 500–1500 K for all studied reactions are given in the form of (*A*, *n*, *E*) in Table 4. The *f* = *k_max_*/*k_min_* and *k*/*k_ave_* in each reaction subclass are also given in these Table 4. Observing Table 4, it is apparent that the ratios between reaction rate constants and the average rate constant for the class, along with the uncertainty factor for the class, fall within a range of one order of magnitude. This signifies that the high-pressure limit rate rules formulated for the *β*-scission class are both reasonable and valid.

#### 2.4.2. Comparison of the High-Pressure Limit Rate Constants with the Kinetic Data for the Corresponding Reactions in Published Mechanisms

**Figure 1:**
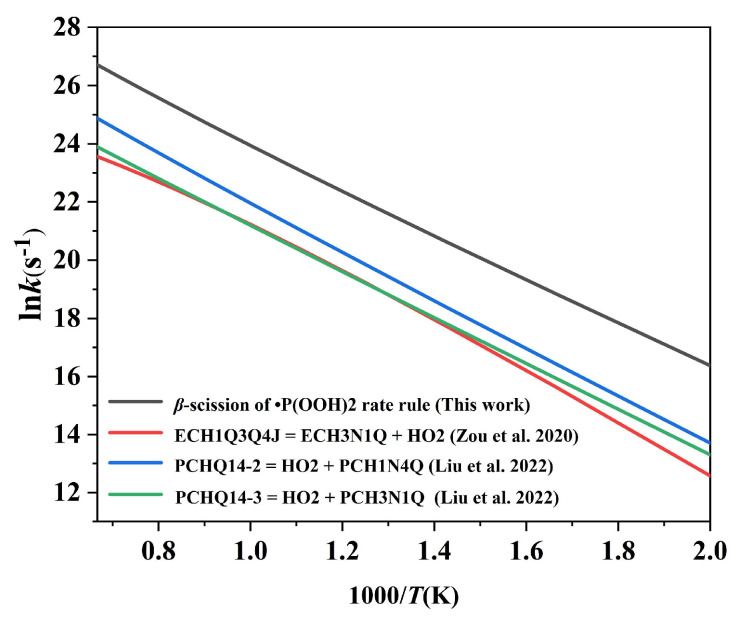
Comparison of the high-pressure limit rate constants: *β*-scission reaction rate rule of •P(OOH)_2_ correspond to ECH1Q3Q4J = ECH3N1Q + HO2 in the mechanism from Zou et al. [32], PCHQ14-2 = HO2 + PCH1N4Q and PCHQ14-3 = HO2 + PCH3N1Q in the mechanism from Liu et al. [27], respectively.

Zou et al. [32] successfully developed a comprehensive combustion mechanism for ethyl cyclohexane at low temperature. Within this mechanism, the high-pressure limit rate constants for the *β*-scission reactions of •P(OOH)_2_ were determined using kinetic data from similar reaction classes. Specifically, these rate constants were derived from the computational results of Fernandes et al. [21] for cyclohexane and Xing et al. [24,25] for methylcyclohexane. Recently, Liu et al. [27] developed an intricate chemical kinetic model that encompasses the entire combustion process of n-propyl cyclohexane, spanning from low to high temperatures. Within this comprehensive mechanism, the rate constants for the *β*-scission reactions of •P(OOH)_2_ were determined based on the kinetic data obtained from the *β*-scission reactions of •QOOH with similar structural characteristics. When the reaction centers reside on the alkyl side chain of n-propyl cyclohexane, the rate constants of the reactions in the mechanism are derived from the kinetic data obtained from *β*-scission reactions occurring within analogous alkane structures [33]. Conversely, when the reaction center is situated within the cyclic structure, the rate constants of the reactions in the mechanism primarily rely on theoretical kinetic studies of cyclohexane with similar structural characteristics [21]. Mao et al. [34,35] have devised mechanisms to replicate the ignition and oxidation of n-butyl cyclohexane across diverse operational scenarios. Nevertheless, the detailed kinetic models they proposed omitted the *β*-scission reactions of •P(OOH)_2_ within n-butyl cyclohexane. Figure 1 presents a comparative analysis of rate constants between our calculated values for high-pressure limit reactions and those from the models developed by Zou et al. for ethyl cyclohexane and Liu et al. for n-propyl cyclohexane. These comparisons were conducted using a temperature range of 500 K to 1500 K. Figure 1 clearly illustrates that the rate constants derived from the *β*-scission of •P(OOH)_2_ rate rule in our study consistently surpass the values of similar reactions found in the mechanisms. This discrepancy is evident with ratio ranges of 14.9 to 44.7, 6.3 to 14.4, and 15.5 to 21.5, respectively. These findings highlight a substantial deviation between our calculated rate constants and those reported in the mechanisms by Zou et al., and Liu et al.

#### 2.4.3. Comparison of the High-Pressure Limit Rate Rule with the Kinetic Data for the *β*-Scission Class in Non-Cyclic Systems

**Figure 2:**
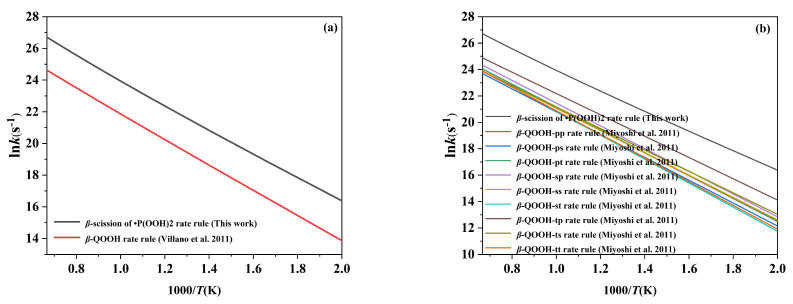
Comparisons of the rate rule derived in this work (black lines) to previously reported rules: (**a**) the reported rules by Villano et al. [33]; (**b**) the reported rules by Miyoshi [36].

Villano et al. [33] systematically investigated the *β*-scission reactions of C2-C6 hydroperoxy alkyl radicals in non-cyclic systems using the CBS-QB3 level of theory. In their study, they assigned a single rate rule for the *β*-scission reaction within this reaction class. Miyoshi [36] reported high-pressure limit rate rules for the *β*-scission class in non-cyclic systems at the CBS-QB3 level of theory. In his study, the *β*-scission class was further categorized into nine distinct subclasses based on the specific carbon atom bonded to the hydroperoxy group and the characteristics of the radical site. For each subclass, Miyoshi developed a unique rate rule. Figure 2 provides comparisons with the literature. In Figure 2a, the rate constants of the *β*-scission of •P(OOH)_2_ rate rule derived in this study are consistently higher than those obtained from the *β*-scission of QOOH rate rule in the study conducted by Villano et al., with a ratio ranging from 7.8 to 12.2. Similarly, Figure 2b illustrates that the rate constants of the *β*-scission of •P(OOH)_2_ rate rule derived in this study are all greater than the values obtained from the *β*-scission of QOOH subclasses rate rules in the study conducted by Miyoshi et al. The respective ratios range from 14.5 to 48.8, 20.5 to 68.8, 14.3 to 47.3, 10.8 to 34.1, 16.6 to 43.1, 16.8 to 101.6, 5.8 to 9.7, 16.1 to 28.5, and 17.2 to 87.2.

### 2.5. Pressure-Dependent Rate Constants and Rate Rules

The *β*-scission of •P(OOH)_2_ is classified as a pressure-dependent channel. To determine the rate constants for this reaction, the RRKM/ME theory is employed. The pressure-dependent rate constants are calculated over a range of pressures (0.01–100 atm) and temperatures (500–1500 K). The calculated pressure-dependent rate constants are presented in Table 5, denoted in the form of (*A*, *n*, *E*). The simple rate rule method is utilized to determine the pressure-dependent rate rules. This method entails averaging the pressure-dependent rate constants for all reactions within each subclass. The resulting pressure-dependent rate rules are fitted into the form of (*A*, *n*, *E*) and presented in Table 5. To enable easier comparisons between different reactions or rate rules, Table 4 also includes the pressure-dependent rate constants at 800 K. The ratios of the rate constants for each reaction to the average rate constant within this class are presented in Table 5, providing a visual representation of the deviation of the rate constants within the class. To evaluate the uncertainty of the rate rule, we define the uncertainty factor as the ratio of the maximum and minimum pressure-dependent rate constants within the class. The uncertainty factors for this class are also listed in Table 5. Based on the findings presented in Table 5, it is evident that the ratios between the rate constants for reactions and the average rate constants within the class, along with the uncertainty factors for the class, fall within the range of 1 to 2 orders of magnitude. This result implies that the pressure-dependent rate rules formulated for the *β*-scission of •P(OOH)_2_ are both reasonable and satisfactory.

#### 2.5.1. Comparison of the Pressure-Dependent Rate Constants with the Kinetic Data for the Corresponding Reactions in Published Mechanisms

**Figure 3:**
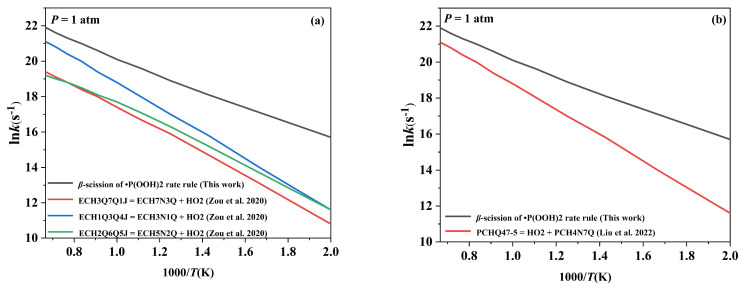
Comparison of the pressure−dependent rate rule derived in this work with the values from the published mechanisms: (**a**) the combustion mechanism constructed by Zou et al. [32]; (**b**) the combustion mechanism constructed by Liu et al. [27].

In this study, we performed a comparative analysis between the pressure-dependent rate rule for the *β*-scission reactions of •P(OOH)_2_ obtained in our research, and the values derived from the combustion mechanisms proposed by Zou et al. [32] for ethyl cyclohexane and Liu et al. [27] for n-propyl cyclohexane at atmospheric pressure. The results are illustrated in Figure 3a and b, respectively. The results presented in Figure 3a clearly demonstrate that the pressure-dependent rate constants calculated in this study are consistently higher than the corresponding values obtained from the combustion mechanism, with ratios ranging from 12.4 to 131.2, 2.3 to 61.4, and 11.8 to 61.3. In Liu’s propyl cyclohexane mechanism, all pressure-dependent rate constants associated with reactions occurring on the rings are based on the same set of kinetic data, derived from the rate constants of *β*-scission reactions observed in similar reaction types in cyclohexane. Therefore, in Figure 3b, we solely focus on comparing the pressure-dependent rate rule obtained in this study at 1 atm with the rate constants of one of these reactions. Based on Figure 3b, it is evident that the pressure-dependent rate rule calculated in our study for the *β*-scission reaction surpasses the corresponding values present in the mechanism, particularly in the lower temperature range. In fact, the ratios can reach almost 60 times at 500 K.

#### 2.5.2. Comparison of the Pressure-Dependent Rate Rules at Different Pressures

**Figure 4:**
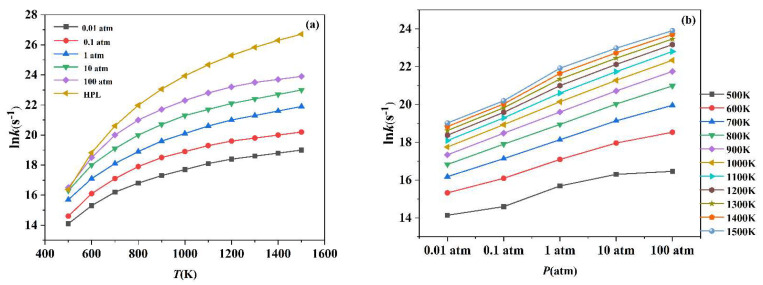
Plot of the calculated average pressure-dependent rate constants of the class *β*-scission of •P(OOH)_2_:(**a**) the dependence on temperatures across the range of 500–1500 K; (**b**) the dependence on pressures across the range of 0.01–100 atm.

The rate constants for the *β*-scission reactions of •P(OOH)_2_ are depicted in Figure 4a, showcasing their dependence on temperatures across the range of 500–1500 K. Figure 4b exhibits the rate constants for this reaction class as a function of pressures, spanning from 0.01 to 100 atm. Notably, Figure 4a reveals that the *β*-scission reactions are highly sensitive to pressure, with the impact of pressure on the rate constants intensifying with increasing temperature. Particularly, this influence becomes significant at temperatures surpassing 700 K. By examining Figure 4b, it becomes evident that reactions belonging to the class of *β*-scission of •P(OOH)_2_ reach their high-pressure limit rate constants at varying pressures at different temperatures. When the temperature is relatively low, for instance, 500 K, the reaction rate constants swiftly reach the high-pressure limit at around 10 atm. However, as the temperature escalates to 800 K, the reaction rate constants exhibit continuous growth across a wide pressure range, spanning from 0.01 atm to 100 atm. Astonishingly, even at the highest pressure of 100 atm, the rate constants have not yet achieved the high-pressure limit. Based on the aforementioned comparison, it is evident that the rate constants associated with the *β*-scission of •P(OOH)_2_ exhibit significant dependence on pressure. This implies that the pressure effect of the *β*-scission reactions within this class cannot be disregarded, particularly at elevated temperatures.

## 3. Methods

### 3.1. Electronic Structure Calculation

The energies of reactants, products, intermediates, and transition states (TS) involved in the reactions are determined using the CBS-QB3 composite method [37] implemented in the Gaussian 16 quantum chemistry software package [38]. In the CBS-QB3 composite method, the geometry optimization and frequency analysis are carried out at the B3LYP/CBSB7 level, and the single-point energy calculations are performed at the CCSD(T)/6−31+G(d’) and MP4SDQ/CBSB4 levels. The total energy is extrapolated to the infinite-basis-set limit using pair natural-orbital energies at the MP2/CBSB3 level and an additive correction to the CCSD(T) level. A correction for spin contamination in open-shell species is added to the total energy. In Supplemental Material-SI Table S1, the comparison between the spin eigenvalues with the S^2^ computed by the Gaussian 16 package for the species involved in this study is given. It is worth noting that the maximum relative error observed is 4.3%. As a result, the spin contamination involved in this study can be safely ignored. To validate the reaction pathways and the connection between reactants and products, we performed an intrinsic reaction coordinate (IRC) analysis [39] at the B3LYP/CBSB7 level. The representative IRCs involving transition states for *β*-scission of •P(OOH)_2_ reactions are provided in the Figure S1 of the Supplemental Material-SI. The optimized structures of all species in this study can be accessed in Supplemental Material-SI, including their corresponding Cartesian coordinates.

### 3.2. Calculation of Rate Constant

In this study, we utilized the Chemrate program [40] to compute the high-pressure limit rate constants and pressure-dependent rate constants for all reactions. The computation of these rate constants is based on the principles of traditional TST [41,42]. The expression for these rate constants is as follows:

(3) $$k\left(T\right)=\kappa \left(T\right)rpd\frac{\kappa_{B}T}{h}\frac{Q_{TS}(T)}{Q_{R}(T)}exp\left[-\frac{V^{\neq }}{RT}\right]$$

The expression provided includes several important parameters. $\kappa \left(T\right)$, rpd, $\kappa_{B}$, and h represent the Eckart tunneling correction factor, reaction path degeneracy, Boltzmann constant, and Planck constant, respectively. The parameter *T* signifies the temperature, and $V^{\neq }$ indicates the magnitude of the energy barrier. Furthermore, $Q_{TS}(T)$ and $Q_{R}(T)$ represent the partition functions of the transition state and the reactants, respectively.

In this study, we demonstrate that the *β*-scission reactions arising from •P(OOH)_2_ exhibit pressure-dependent characteristics. The rate constants’ sensitivity to pressure is thoroughly investigated using RRKM/ME theory [43]. To simulate the reaction environment, argon (Ar) is chosen as the bath gas, and the collision frequency between the reactant and the bath gas is estimated utilizing the Lennard-Jones (L-J) parameters σ (in Å) and ε (in K). To determine these L-J parameters, we employ the method proposed by Wang and Frenklach [44]. The σ and ε values employed in this study are outlined in Table S2 of Supplemental Material-SI.

In this study, we apply the one-dimensional asymmetric Eckart tunneling correction method [45,46] to account for tunneling effects. To rectify the substantial errors associated with approximating low-frequency vibrations of single bond torsions harmonically [47], we utilize a one-dimensional (1-D) hindered rotor model [48,49] to accurately represent the low-frequency vibrations of single bond torsions in the reaction. To determine the torsional potential energy of the reactants, we employed the B3LYP/CBSB7 theoretical level and conducted a dihedral angle scan at 10-degree intervals. To ensure the integrity of the reaction center, the atoms involved are initially fixed during the scanning process for the transition state. The potential energy profiles for internal rotations about C-C, C-OO, and CO-OH single bonds of the reactant, the transition state, and the product in a representative reaction are provided in the Figure S2 of the Supplemental Material-SI. In addition, in order to facilitate combustion modeling using widely-used software such as Chemkin-PRO (15092) [50], the thermodynamic parameters, namely enthalpy, entropy, and heat capacity, were fitted using the NASA format’s 14 parameters. The outcomes of this fitting process can be found in Supplemental Material-SII.

In conclusion, the rate constants within the temperature range of 500 K to 1500 K are determined by fitting them using the three-parameter form of the Arrhenius Equation:

(4) $$k\left(T\right)=AT^{n}\exp(-\frac{E}{RT})$$

## 4. Conclusions

In this research study, systematic calculations were performed to determine the energy barriers, high-pressure limit rate constants, and pressure-dependent rate constants of *β*-scission reactions of •P(OOH)_2_ compounds in normal-alkyl cyclohexanes. The calculations were carried out over a temperature range of 500–1500 K and a pressure range of 0.01–100 atm. The following conclusions can be drawn from our study: (1) The energy barriers and rate constants for the *β*-scission of •P(OOH)_2_ reactions in our calculations did not exhibit significant variations. As a result, there is no need to further categorize these reactions into different subclasses. Instead, a comprehensive high-pressure limit rate rule and pressure-dependent rate rule can be established for this reaction class. (2) The maximum deviation in energy barriers and the uncertainty factor associated with the rate rule within this class were found to be within the bounds of chemical accuracy. This demonstrates the reliability and acceptability of our classification schemes. (3) The rate constants for the *β*-scission reactions of •P(OOH)_2_ in normal-alkyl cyclohexanes exhibit significant discrepancies compared to the kinetic data provided in certain published mechanisms. This highlights the critical need to establish accurate rate rules for the *β*-scission reactions of •P(OOH)_2_ of normal-alkyl cyclohexanes. (4) Pressure exerts a substantial influence on the rate constants of the *β*-scission reactions of •P(OOH)_2_ of normal-alkyl cyclohexanes. Hence, our precisely-computed high-pressure limit rate rule and pressure-dependent rate rules are of immense importance, as they contribute to advancing the automatic generation of combustion mechanisms for normal-alkyl cyclohexanes.

## Figures and Tables

**Table 1:** A comparison of the energy barriers for the reaction R17 with and without hydrogen bond conformation (unit: kcal mol^−1^).

	Reactants		Transition States		Energy Barriers
(1)	R-no HB	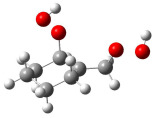	TS-no HB	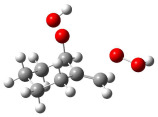	16.4
(2)	R-HB	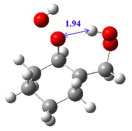	TS-no HB	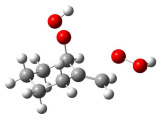	19.9
(3)	R-no HB	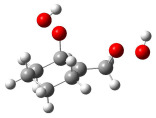	TS-HB	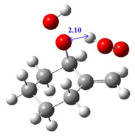	12.7
(4)	R-HB	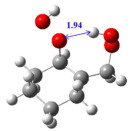	TS-HB	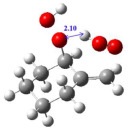	16.2

**Table 2:** Energy barriers for the *β*-scission reactions of •P(OOH)_2_ (kcal mol^−1^).

	Reaction	Energy Barrier
R1	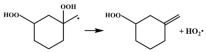	16.3
R2	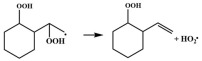	15.1
R3	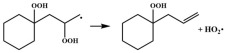	14.8
R4	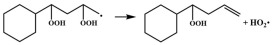	15.4
R5	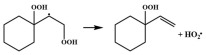	14.5
R6	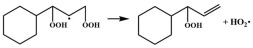	14.6
R7	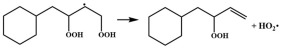	15.7
R8	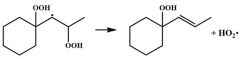	15.3
R9	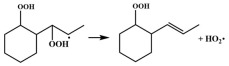	14.7
R10	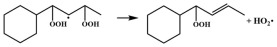	14.6
R11	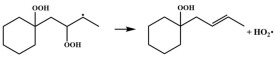	14.0
R12	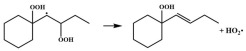	15.9
R13	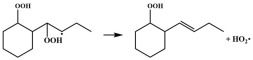	14.5
R14	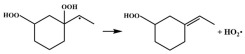	16.5
R15	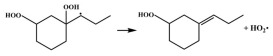	16.4
R16	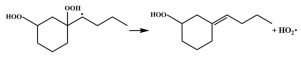	16.3
R17	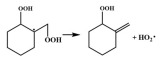	16.2
R18	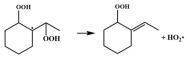	15.6
R19	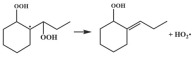	15.8
R20	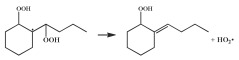	15.6
R21	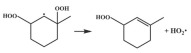	14.7
R22	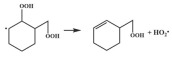	15.9
R23	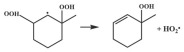	14.8
R24	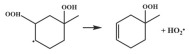	15.3
R25	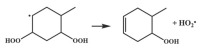	14.8
R26	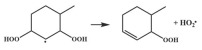	16.4
R27	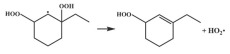	15.0
R28	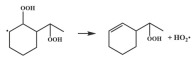	16.6
R29	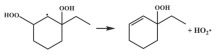	15.1
R30	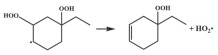	15.2
R31	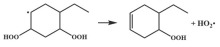	14.8
R32	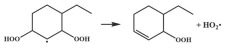	16.4
R33	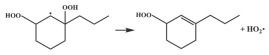	14.9
R34	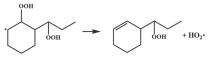	16.8
R35	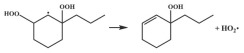	15.1
R36	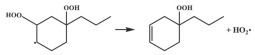	15.2
R37	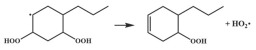	14.8
R38	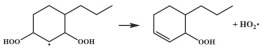	16.4
R39	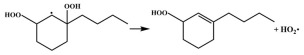	14.9
R40	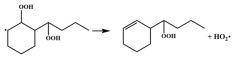	16.8
R41	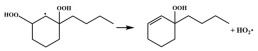	15.2
R42	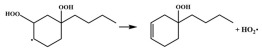	15.3
R43	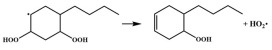	14.8
R44	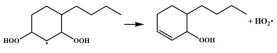	16.5
***β*-scission class**		**(15.5 ^a^, 2.8 ^b^)**

^a^ The average energy barrier for reactions within this class. ^b^ The maximum deviation for reactions within this class.

**Table 3:** Comparison of the energy barriers between the *β*-scission reactions of •QOOH and those of •P(OOH)_2_ (kcal mol^−1^).

*β*-Scission of •P(OOH)_2_ This Work	Energy Barrier	*β*-Scission of •QOOH Previous Work	Energy Barrier
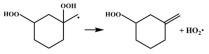	16.3	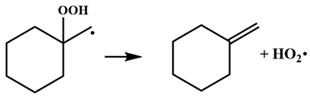	16.0
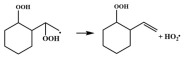	15.1	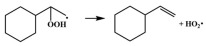	16.0
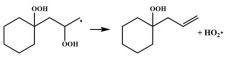	14.8	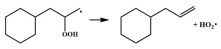	15.9
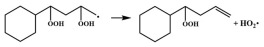	15.4	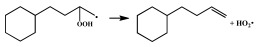	16.2
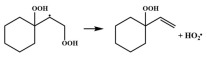	14.5	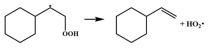	15.6
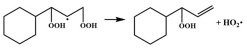	14.6	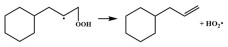	16.1
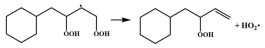	15.7	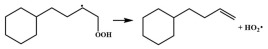	16.0
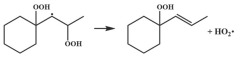	15.3	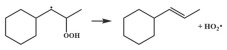	15.3
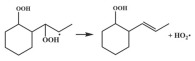	14.7	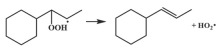	15.3
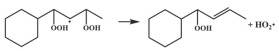	14.6	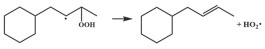	16.3
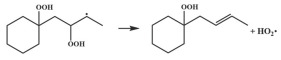	14.0	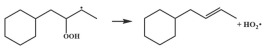	15.6
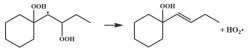	15.9	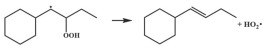	15.7
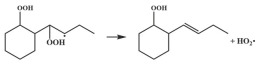	14.5	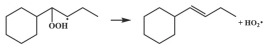	15.2
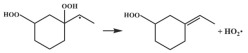	16.5	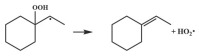	16.6
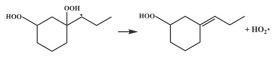	16.4	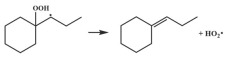	16.5
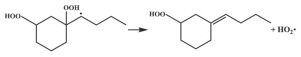	16.3	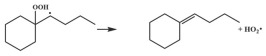	16.4
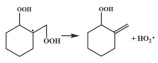	16.2	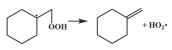	14.4
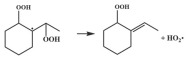	15.6	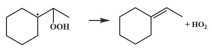	14.1
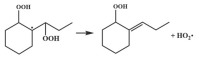	15.8	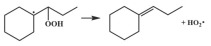	14.0
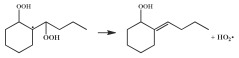	15.6	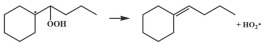	13.9
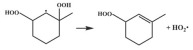	14.7	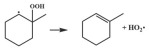	15.3
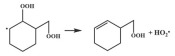	15.9	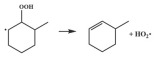	15.6
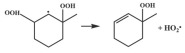	14.8	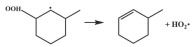	15.2
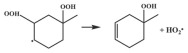	15.3	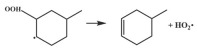	15.0
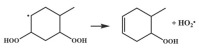	14.8	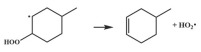	15.1
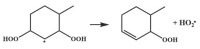	16.4	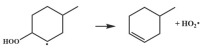	15.1
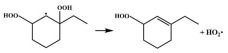	15.0	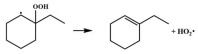	15.2
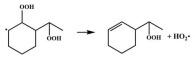	16.6	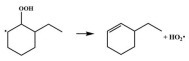	15.6
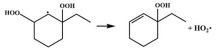	15.1	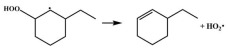	15.1
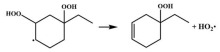	15.2	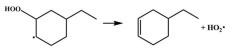	14.9
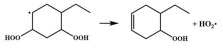	14.8	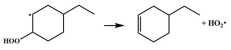	15.1
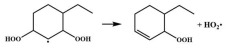	16.4	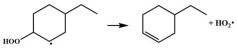	15.1
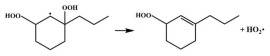	14.9	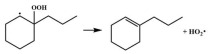	15.1
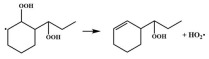	16.8	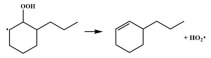	15.7
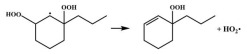	15.1	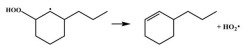	15.1
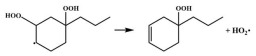	15.2	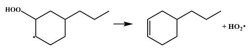	15.0
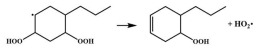	14.8	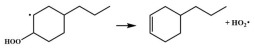	15.1
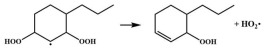	16.4	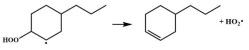	15.1
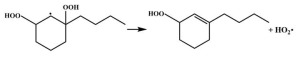	14.9	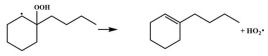	15.2
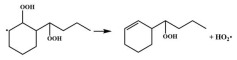	16.8	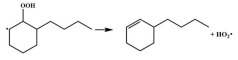	15.6
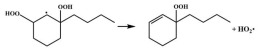	15.2	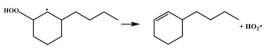	15.0
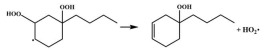	15.3	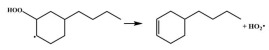	15.0
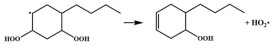	14.8	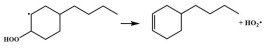	15.1
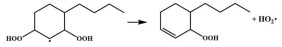	16.5	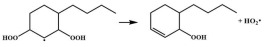	15.1

**Table 4:** Analysis of rate constants, rate ratios, and uncertainty factor in *β*-scission class at high pressure.

	Modified Arrhenius Parameters			*T* = 800 K	
Reaction	*A* (s^−1^)	*n*	*E* (cal mol^−1^)	*k* (s^−1^)	*k*/*k_ave_^e^*
R1	7.89 × 10^10^	0.68	14,825	6.53 × 10^8^	0.2
R2	3.30 × 10^10^	0.72	12,917.3	1.21 × 10^9^	0.4
R3	1.76 × 10^14^	−0.47	13,467.6	1.57 × 10^9^	0.6
R4	1.69 × 10^21^	−2.79	15,678.3	6.97 × 10^8^	0.3
R5	8.10 × 10^10^	0.57	12,586.8	1.29 × 10^9^	0.5
R6	1.62 × 10^11^	0.57	12,591	2.58 × 10^9^	0.9
R7	3.42 × 10^12^	0.33	14,195.2	4.24 × 10^9^	1.5
R8	1.30 × 10^15^	−0.53	15,412.3	2.35 × 10^9^	0.8
R9	1.54 × 10^14^	−0.46	14,286.6	9.02 × 10^8^	0.3
R10	6.39 × 10^14^	−0.61	14,562.3	1.11 × 10^9^	0.4
R11	5.28 × 10^10^	0.56	12,988.8	6.37 × 10^8^	0.2
R12	1.88 × 10^14^	−0.20	16,039.2	2.10 × 10^9^	0.8
R13	6.92 × 10^13^	−0.14	14,058.2	3.80 × 10^9^	1.4
R14	8.06 × 10^10^	0.75	15,413.8	7.54 × 10^8^	0.3
R15	8.91 × 10^15^	−0.65	16,530.5	3.58 × 10^9^	1.3
R16	3.21 × 10^14^	−0.21	16,328.9	2.75 × 10^9^	1.0
R17	3.37 × 10^12^	0.29	14,444.9	2.62 × 10^9^	0.9
R18	2.11 × 10^12^	0.39	14,762.5	2.70 × 10^9^	1.0
R19	1.21 × 10^15^	−0.41	15,684.1	4.14 × 10^9^	1.5
R20	2.00 × 10^10^	1.00	14,149.3	2.25 × 10^9^	0.8
R21	1.11 × 10^11^	0.81	13,327.4	5.60 × 10^9^	2.0
R22	7.51 × 10^12^	0.45	15,685.9	7.67 × 10^9^	2.8
R23	4.22 × 10^11^	0.43	13,543.5	1.46 × 10^9^	0.5
R24	4.67 × 10^11^	0.37	14,010.2	8.15 × 10^8^	0.3
R25	5.82 × 10^11^	0.39	13,522.5	1.65 × 10^9^	0.6
R26	4.46 × 10^11^	0.49	15,149.1	8.33 × 10^8^	0.3
R27	1.31 × 10^14^	−0.14	15,881.9	2.40 × 10^9^	0.9
R28	4.37 × 10^14^	0.01	17,887.1	6.07 × 10^9^	2.2
R29	7.82 × 10^13^	−0.17	15,816.1	1.23 × 10^9^	0.4
R30	5.75 × 10^13^	−0.17	15,859.5	8.64 × 10^8^	0.3
R31	1.06 × 10^12^	0.43	13,397.7	3.97 × 10^9^	1.4
R32	9.02 × 10^11^	0.48	15,188.5	1.62 × 10^9^	0.6
R33	1.68 × 10^12^	0.44	13,802.2	5.40 × 10^9^	1.9
R34	1.20 × 10^11^	0.99	16,357	3.11 × 10^9^	1.1
R35	2.14 × 10^12^	0.42	13,842.7	5.82 × 10^9^	2.1
R36	2.43 × 10^12^	0.31	14,131.4	2.61 × 10^9^	0.9
R37	1.98 × 10^12^	0.39	13,496.6	5.50 × 10^9^	2.0
R38	9.25 × 10^12^	0.44	16,624	4.96 × 10^9^	1.8
R39	6.00 × 10^11^	0.43	13,791.4	1.85 × 10^9^	0.7
R40	1.33 × 10^10^	1.43	15,998.7	8.14 × 10^9^	2.9
R41	1.73 × 10^11^	0.53	13,585.9	1.15 × 10^9^	0.4
R42	1.86 × 10^14^	−0.17	15,912.6	2.75 × 10^9^	1.0
R43	1.09 × 10^12^	0.40	13,427.9	3.32 × 10^9^	1.2
R44	1.04 × 10^12^	0.47	15,255.5	1.63 × 10^9^	0.6
***β* scission class rate rule**	**8.47 × 10^9^**	**1.11**	**13,585.5**	**2.78 × 10^9^**	**12.8 ^#^**

*^e^ k_ave_* is the average rate constants at 800 K for class. ^#^ The uncertainty factor values of the high-pressure limit rule at 800 K for class in our work (The ratio of the largest rate constant to the smallest rate constant at 800 K for class).

**Table 5:** Calculated rate constants, ratios, and uncertainty factor in *β*-scission class at different pressures.

		Modified Arrhenius Parameters			*T* = 800 K	
Reaction	Pressure (atm)	*A* (s^−1^)	*n*	*E* (cal mol^−1^)	*k* (s^−1^)	*k*/*k_ave_^e^*
R1	0.01	1.99 × 10^12^	−1.10	8262.1	6.9 × 10^6^	0.5
	0.1	2.05 × 10^12^	−0.89	9158.4	1.7 × 10^7^	0.4
	1	6.57 × 10^15^	−1.66	11,742.7	6.2 × 10^7^	0.4
	10	2.81 × 10^24^	−3.94	17,150.0	2.1 × 10^8^	0.4
	100	1.18 × 10^28^	−4.75	20,526.9	4.7 × 10^8^	0.4
R2	0.01	5.96 × 10^10^	−0.66	6625.0	1.1 × 10^7^	0.8
	0.1	1.25 × 10^29^	−5.87	16,057.4	4.6 × 10^7^	1.2
	1	1.48 × 10^13^	−0.77	10,088.1	1.5 × 10^8^	1.0
	10	1.00 × 10^20^	−2.54	14,480.4	4.7 × 10^8^	1.0
	100	8.13 × 10^21^	−2.88	16,729.3	9.2 × 10^8^	0.7
R3	0.01	1.11 × 10^12^	−1.03	6829.8	1.5 × 10^7^	1.1
	0.1	6.54 × 10^37^	−8.51	19,359.7	6.8 × 10^7^	1.8
	1	1.74 × 10^44^	−10.13	23,253.2	3.0 × 10^8^	2.1
	10	6.07 × 10^18^	−2.19	13,287.4	6.2 × 10^8^	1.3
	100	5.04 × 10^21^	−2.86	15,842.2	1.2 × 10^9^	1.0
R4	0.01	1.05 × 10^12^	−1.01	7003.7	1.5 × 10^7^	1.1
	0.1	2.60 × 10^30^	−6.26	16,431.0	5.5 × 10^7^	1.4
	1	2.81 × 10^13^	−0.87	9953.5	1.6 × 10^8^	1.1
	10	3.57 × 10^19^	−2.47	13,813.7	4.1 × 10^8^	0.9
	100	1.66 × 10^22^	−3.13	15,871.5	6.4 × 10^8^	0.5
R5	0.01	1.65 × 10^11^	−0.79	6515.2	1.4 × 10^7^	1.0
	0.1	3.02 × 10^34^	−7.50	18,035.6	6.0 × 10^7^	1.5
	1	5.24 × 10^21^	−3.35	13,502.0	2.0 × 10^8^	1.4
	10	6.29 × 10^18^	−2.19	13,553.5	5.5 × 10^8^	1.2
	100	6.48 × 10^20^	−2.57	15,823.8	1.1 × 10^9^	0.9
R6	0.01	1.18 × 10^12^	−1.05	6650.9	1.6 × 10^7^	1.2
	0.1	3.54 × 10^42^	−9.96	21,053.6	7.8 × 10^7^	2.0
	1	2.01 × 10^47^	−11.05	24,331.0	3.8 × 10^8^	2.6
	10	8.61 × 10^18^	−2.22	13,030.2	8.3 × 10^8^	1.8
	100	2.49 × 10^23^	−3.32	16,399.4	1.9 × 10^9^	1.5
R7	0.01	4.21 × 10^12^	−1.21	7051.3	1.6 × 10^7^	1.1
	0.1	3.44 × 10^7^	0.63	5985.7	5.3 × 10^7^	1.4
	1	4.77 × 10^−67^	23.12	−25,993.3	7.7 × 10^7^	0.5
	10	4.41 × 10^−43^	16.20	−14,049.8	3.3 × 10^8^	0.7
	100	4.84 × 10^−16^	8.37	−404.5	1.2 × 10^9^	1.0
R8	0.01	3.33 × 10^11^	−0.87	7033.6	1.2 × 10^7^	0.9
	0.1	7.94 × 10^38^	−8.81	20,325.8	5.9 × 10^7^	1.5
	1	5.62 × 10^48^	−11.45	25,781.3	3.0 × 10^8^	2.0
	10	7.09 × 10^22^	−3.35	15,688.8	6.9 × 10^8^	1.5
	100	4.55 × 10^28^	−4.84	19,707.2	1.6 × 10^9^	1.3
R9	0.01	2.64 × 10^11^	−0.84	6999.6	1.2 × 10^7^	0.9
	0.1	2.84 × 10^28^	−5.66	15,908.9	4.6 × 10^7^	1.2
	1	6.71 × 10^13^	−0.96	10,556.9	1.4 × 10^8^	1.0
	10	3.60 × 10^19^	−2.40	14,479.2	4.2 × 10^8^	0.9
	100	9.10 × 10^21^	−2.92	16,844.3	7.7 × 10^8^	0.6
R10	0.01	1.36 × 10^12^	−1.04	7226.5	1.4 × 10^7^	1.0
	0.1	5.46 × 10^33^	−7.25	18,099.7	5.5 × 10^7^	1.4
	1	3.54 × 10^13^	−0.86	10,347.0	1.7 × 10^8^	1.1
	10	7.96 × 10^20^	−2.79	14,975.2	5.0 × 10^8^	1.1
	100	1.13 × 10^23^	−3.22	17,261.3	9.5 × 10^8^	0.8
R11	0.01	9.06 × 10^10^	−0.70	6777.2	1.2 × 10^7^	0.9
	0.1	5.82 × 10^10^	−0.47	7375.1	2.4 × 10^7^	0.6
	1	3.62 × 10^14^	−1.34	10,068.0	8.1 × 10^7^	0.6
	10	7.72 × 10^21^	−3.26	14,810.5	2.4 × 10^8^	0.5
	100	4.46 × 10^24^	−3.83	17,659.8	5.0 × 10^8^	0.4
R12	0.01	1.07 × 10^10^	−0.41	6594.7	1.1 × 10^7^	0.8
	0.1	6.71 × 10^13^	−1.35	9102.0	2.6 × 10^7^	0.7
	1	4.32 × 10^16^	−1.91	11,204.9	1.0 × 10^8^	0.7
	10	7.66 × 10^22^	−3.49	15,299.8	3.8 × 10^8^	0.8
	100	4.45 × 10^29^	−5.21	20,083.2	1.1 × 10^9^	0.8
R13	0.01	2.47 × 10^0^	2.45	1496.2	1.3 × 10^7^	0.9
	0.1	1.13 × 10^8^	0.37	5701.2	3.8 × 10^7^	1.0
	1	9.51 × 10^13^	−1.15	8877.3	1.6 × 10^8^	1.1
	10	1.85 × 10^18^	−2.17	11,771.2	5.8 × 10^8^	1.2
	100	8.37 × 10^24^	−3.88	16,277.2	1.6 × 10^9^	1.3
R14	0.01	2.60 × 10^13^	−1.42	8856.5	7.2 × 10^6^	0.5
	0.1	7.88 × 10^17^	−2.56	11,783.8	1.8 × 10^7^	0.5
	1	3.58 × 10^17^	−2.18	12,665.9	6.0 × 10^7^	0.4
	10	4.24 × 10^25^	−4.29	17,845.4	2.0 × 10^8^	0.4
	100	6.83 × 10^29^	−5.25	21,550.1	4.9 × 10^8^	0.4
R15	0.01	5.39 × 10^7^	0.26	5428.8	1.0 × 10^7^	0.7
	0.1	1.90 × 10^12^	−0.88	8313.7	2.9 × 10^7^	0.8
	1	4.87 × 10^15^	−1.62	10,489.8	1.3 × 10^8^	0.9
	10	7.14 × 10^20^	−2.88	13,890.4	5.0 × 10^8^	1.1
	100	2.95 × 10^28^	−4.86	18,946.0	1.5 × 10^9^	1.2
R16	0.01	1.84 × 10^9^	−0.18	6215.5	1.1 × 10^7^	0.8
	0.1	1.45 × 10^14^	−1.45	9211.8	2.8 × 10^7^	0.7
	1	1.29 × 10^17^	−2.05	11,335.5	1.1 × 10^8^	0.8
	10	5.61 × 10^22^	−3.44	15,100.8	4.3 × 10^8^	0.9
	100	6.95 × 10^29^	−5.26	20,023.1	1.3 × 10^9^	1.0
R17	0.01	2.25 × 10^6^	0.64	4682.3	8.6 × 10^6^	0.6
	0.1	5.97 × 10^8^	0.16	6662.7	2.6 × 10^7^	0.7
	1	2.31 × 10^11^	−0.35	8454.8	1.1 × 10^8^	0.8
	10	8.94 × 10^17^	−2.04	12,490.5	4.2 × 10^8^	0.9
	100	3.25 × 10^25^	−4.01	17,488.3	1.2 × 10^9^	1.0
R18	0.01	1.87 × 10^8^	0.08	5649.0	9.4 × 10^6^	0.7
	0.1	5.99 × 10^10^	−0.44	7649.2	2.6 × 10^7^	0.7
	1	2.50 × 10^13^	−0.95	9495.1	1.1 × 10^8^	0.8
	10	3.68 × 10^19^	−2.51	13,392.2	4.2 × 10^8^	0.9
	100	8.87 × 10^26^	−4.42	18,359.7	1.2 × 10^9^	1.0
R19	0.01	1.63 × 10^5^	1.01	4078.0	1.1 × 10^7^	0.8
	0.1	1.17 × 10^10^	−0.21	7092.7	3.2 × 10^7^	0.8
	1	2.14 × 10^14^	−1.23	9561.2	1.4 × 10^8^	1.0
	10	1.27 × 10^19^	−2.37	12,701.4	5.6 × 10^8^	1.2
	100	5.15 × 10^26^	−4.36	17,686.6	1.7 × 10^9^	1.4
R20	0.01	3.33 × 10^8^	0.03	5695.6	1.1 × 10^7^	0.8
	0.1	2.80 × 10^12^	−0.95	8256.4	2.8 × 10^7^	0.7
	1	5.24 × 10^15^	−1.65	10,516.9	1.1 × 10^8^	0.8
	10	3.03 × 10^21^	−3.08	14,356.4	4.0 × 10^8^	0.9
	100	1.07 × 10^28^	−4.74	19,031.9	1.1 × 10^9^	0.9
R21	0.01	9.08 × 10^1^	1.95	2409.0	9.3 × 10^6^	0.7
	0.1	5.65 × 10^7^	0.47	5813.9	3.5 × 10^7^	0.9
	1	4.28 × 10^10^	−0.13	7557.5	1.6 × 10^8^	1.1
	10	1.19 × 10^15^	−1.19	10,382.0	6.3 × 10^8^	1.3
	100	7.69 × 10^22^	−3.24	15,314.7	2.0 × 10^9^	1.6
R22	0.01	5.41 × 10^4^	1.14	4218.3	7.8 × 10^6^	0.6
	0.1	1.18 × 10^9^	0.10	7015.4	2.8 × 10^7^	0.7
	1	3.71 × 10^11^	−0.37	8628.7	1.4 × 10^8^	1.0
	10	4.60 × 10^16^	−1.61	11,796.9	5.9 × 10^8^	1.3
	100	2.63 × 10^25^	−3.93	17,215.9	2.1 × 10^9^	1.7
R23	0.01	6.70 × 10^8^	−0.10	5765.5	9.2 × 10^6^	0.7
	0.1	1.24 × 10^10^	−0.26	7201.3	2.4 × 10^7^	0.6
	1	2.19 × 10^12^	−0.66	8905.8	9.6 × 10^7^	0.7
	10	1.45 × 10^19^	−2.43	13,136.9	3.4 × 10^8^	0.7
	100	9.74 × 10^24^	−3.90	17,358.5	8.6 × 10^8^	0.7
R24	0.01	1.66 × 10^11^	−0.80	7216.6	8.2 × 10^6^	0.6
	0.1	3.62 × 10^12^	−1.00	8681.2	2.0 × 10^7^	0.5
	1	1.35 × 10^14^	−1.20	10,249.4	7.2 × 10^7^	0.5
	10	4.85 × 10^21^	−3.17	14,987.9	2.4 × 10^8^	0.5
	100	8.59 × 10^25^	−4.17	18,547.1	5.6 × 10^8^	0.5
R25	0.01	2.34 × 10^8^	0.04	5540.1	9.2 × 10^6^	0.7
	0.1	9.09 × 10^9^	−0.21	7108.2	2.5 × 10^7^	0.6
	1	1.55 × 10^12^	−0.62	8769.5	1.0 × 10^8^	0.7
	10	6.96 × 10^18^	−2.33	12,888.1	3.5 × 10^8^	0.8
	100	7.32 × 10^24^	−3.86	17,180.7	9.1 × 10^8^	0.7
R26	0.01	2.35 × 10^12^	−1.13	8199.2	6.9 × 10^6^	0.5
	0.1	2.13 × 10^14^	−1.50	10,006.9	1.7 × 10^7^	0.4
	1	4.64 × 10^15^	−1.63	11,580.4	6.1 × 10^7^	0.4
	10	8.18 × 10^23^	−3.79	16,759.2	2.1 × 10^8^	0.4
	100	1.55 × 10^28^	−4.79	20,430.8	5.0 × 10^8^	0.4
R27	0.01	6.87 × 10^10^	−0.67	7097.7	8.8 × 10^6^	0.6
	0.1	2.88 × 10^13^	−1.23	9126.3	2.4 × 10^7^	0.6
	1	1.98 × 10^15^	−1.51	10,662.6	1.0 × 10^8^	0.7
	10	4.02 × 10^21^	−3.10	14,657.0	3.9 × 10^8^	0.8
	100	6.16 × 10^28^	−4.96	19,560.0	1.1 × 10^9^	0.9
R28	0.01	2.03 × 10^11^	−0.80	7747.2	7.4 × 10^6^	0.5
	0.1	8.46 × 10^13^	−1.34	9817.3	2.3 × 10^7^	0.6
	1	9.23 × 10^15^	−1.66	11,416.7	1.1 × 10^8^	0.7
	10	1.16 × 10^22^	−3.18	15,260.7	4.6 × 10^8^	1.0
	100	7.73 × 10^30^	−5.50	20,920.8	1.6 × 10^9^	1.3
R29	0.01	2.64 × 10^12^	−1.14	8058.9	8.3 × 10^6^	0.6
	0.1	7.61 × 10^15^	−1.97	10,446.3	2.1 × 10^7^	0.5
	1	3.06 × 10^16^	−1.87	11,604.9	7.7 × 10^7^	0.5
	10	7.65 × 10^23^	−3.79	16,287.9	2.7 × 10^8^	0.6
	100	1.78 × 10^29^	−5.11	20,420.3	6.8 × 10^8^	0.5
R30	0.01	1.12 × 10^13^	−1.32	8531.6	7.9 × 10^6^	0.6
	0.1	2.44 × 10^17^	−2.41	11,325.2	1.9 × 10^7^	0.5
	1	1.90 × 10^17^	−2.11	12,232.9	6.6 × 10^7^	0.5
	10	1.07 × 10^25^	−4.13	17,166.4	2.3 × 10^8^	0.5
	100	2.51 × 10^29^	−5.16	20,859.7	5.3 × 10^8^	0.4
R31	0.01	5.81 × 10^2^	1.73	2660.4	1.1 × 10^7^	0.8
	0.1	5.18 × 10^8^	0.18	6095.2	3.8 × 10^7^	1.0
	1	3.37 × 10^12^	−0.70	8305.1	1.7 × 10^8^	1.1
	10	1.56 × 10^17^	−1.83	11,349.6	6.1 × 10^8^	1.3
	100	1.94 × 10^24^	−3.67	16,046.1	1.8 × 10^9^	1.4
R32	0.01	5.16 × 10^11^	−0.93	7533.6	9.1 × 10^6^	0.7
	0.1	5.34 × 10^13^	−1.32	9278.3	2.3 × 10^7^	0.6
	1	5.11 × 10^15^	−1.63	11,035.1	9.3 × 10^7^	0.6
	10	8.96 × 10^22^	−3.50	15,647.4	3.4 × 10^8^	0.7
	100	1.26 × 10^29^	−5.04	20,177.3	9.1 × 10^8^	0.7
R33	0.01	6.72 × 10^0^	2.32	1775.7	1.2 × 10^7^	0.9
	0.1	3.75 × 10^8^	0.23	5997.9	4.1 × 10^7^	1.1
	1	6.42 × 10^13^	−1.08	8835.2	1.8 × 10^8^	1.3
	10	9.82 × 10^17^	−2.06	11,661.9	6.8 × 10^8^	1.5
	100	1.01 × 10^25^	−3.87	16,334.4	2.1 × 10^9^	1.7
R34	0.01	1.83 × 10^12^	−1.07	8069.1	9.0 × 10^6^	0.7
	0.1	4.35 × 10^34^	−7.48	19,352.7	4.2 × 10^7^	1.1
	1	1.55 × 10^50^	−11.83	27,391.5	2.3 × 10^8^	1.6
	10	2.15 × 10^27^	−4.61	18,838.3	6.2 × 10^8^	1.3
	100	1.40 × 10^37^	−7.28	24,814.8	1.7 × 10^9^	1.4
R35	0.01	1.56 × 10^0^	2.51	1477.3	1.2 × 10^7^	0.9
	0.1	2.45 × 10^8^	0.29	5897.7	4.2 × 10^7^	1.1
	1	6.29 × 10^13^	−1.07	8803.5	1.9 × 10^8^	1.3
	10	7.40 × 10^17^	−2.02	11,560.8	7.1 × 10^8^	1.5
	100	7.65 × 10^24^	−3.83	16,221.7	2.2 × 10^9^	1.7
R36	0.01	1.38 × 10^6^	0.74	4434.7	1.2 × 10^7^	0.9
	0.1	1.44 × 10^11^	−0.55	7472.1	3.2 × 10^7^	0.8
	1	5.96 × 10^14^	−1.37	9734.3	1.4 × 10^8^	0.9
	10	1.62 × 10^20^	−2.72	13,314.0	4.9 × 10^8^	1.0
	100	5.73 × 10^26^	−4.39	17,893.3	1.3 × 10^9^	1.1
R37	0.01	3.19 × 10^−2^	3.02	650.4	1.2 × 10^7^	0.9
	0.1	6.47 × 10^7^	0.46	5539.3	4.4 × 10^7^	1.1
	1	3.00 × 10^13^	−0.98	8534.6	2.0 × 10^8^	1.3
	10	2.62 × 10^17^	−1.89	11,200.8	7.3 × 10^8^	1.6
	100	1.98 × 10^24^	−3.67	15,768.8	2.2 × 10^9^	1.8
R38	0.01	2.98 × 10^11^	−0.84	7443.0	1.0 × 10^7^	0.7
	0.1	1.13 × 10^40^	−9.13	21,293.2	5.3 × 10^7^	1.4
	1	1.08 × 10^53^	−12.70	28,151.8	3.1 × 10^8^	2.1
	10	1.04 × 10^28^	−4.84	18,452.6	8.3 × 10^8^	1.8
	100	1.36 × 10^39^	−7.91	24,879.1	2.4 × 10^9^	1.9
R39	0.01	9.00 × 10^7^	0.20	5231.3	1.3 × 10^7^	0.9
	0.1	5.74 × 10^11^	−0.75	7668.5	3.1 × 10^7^	0.8
	1	3.11 × 10^15^	−1.60	10,090.1	1.2 × 10^8^	0.9
	10	1.52 × 10^21^	−3.02	13,889.6	4.2 × 10^8^	0.9
	100	7.59 × 10^26^	−4.44	18,121.7	1.1 × 10^9^	0.9
R40	0.01	2.76 × 10^8^	0.08	6012.2	1.1 × 10^7^	0.8
	0.1	3.06 × 10^12^	−0.89	8635.5	3.4 × 10^7^	0.9
	1	1.37 × 10^17^	−2.00	11,403.5	1.6 × 10^8^	1.1
	10	5.27 × 10^22^	−3.36	15,108.0	6.8 × 10^8^	1.5
	100	1.21 × 10^31^	−5.54	20,636.8	2.3 × 10^9^	1.9
R41	0.01	2.69 × 10^10^	−0.53	6504.2	1.3 × 10^7^	0.9
	0.1	5.85 × 10^13^	−1.36	8680.1	2.8 × 10^7^	0.7
	1	1.14 × 10^16^	−1.78	10,614.9	9.9 × 10^7^	0.7
	10	2.25 × 10^22^	−3.37	14,822.9	3.2 × 10^8^	0.7
	100	4.55 × 10^26^	−4.39	18,443.6	7.5 × 10^8^	0.6
R42	0.01	9.98 × 10^28^	−5.16	21,464.5	1.4 × 10^8^	10.1
	0.1	1.01 × 10^22^	−2.90	19,477.2	1.8 × 10^8^	4.7
	1	1.21 × 10^15^	−0.77	16,789.5	1.8 × 10^8^	1.3
	10	1.96 × 10^13^	−0.22	16,057.5	1.8 × 10^8^	0.4
	100	1.19 × 10^13^	−0.15	15,968.0	1.8 × 10^8^	0.1
R43	0.01	1.92 × 10^1^	2.19	1916.8	1.3 × 10^7^	0.3
	0.1	4.53 × 10^8^	0.20	5964.1	4.0 × 10^7^	1.3
	1	1.80 × 10^14^	−1.23	9024.6	1.7 × 10^8^	2.3
	10	7.77 × 10^18^	−2.34	12,143.8	5.9 × 10^8^	3.3
	100	2.31 × 10^25^	−4.00	16,615.7	1.6 × 10^9^	4.3
R44	0.01	3.11 × 10^11^	−0.84	7368.0	1.1 × 10^7^	0.8
	0.1	3.17 × 10^15^	−1.85	9912.4	2.6 × 10^7^	0.7
	1	3.94 × 10^17^	−2.20	11,797.4	1.0 × 10^8^	0.7
	10	1.90 × 10^24^	−3.89	16,222.3	3.5 × 10^8^	0.8
	100	1.19 × 10^30^	−5.33	20,629.3	9.3 × 10^8^	0.7
***β*-scission class rate rule**	**0.01**	**3.02 × 10^−10^**	**5.70**	**−579.1**	**1.4 × 10^7^**	**20.0 ^#^**
	**0.1**	**4.78 × 10^9^**	**0.005**	**7720.47**	**3.9 × 10^7^**	**10.9 ^#^**
	**1**	**7.93 × 10^3^**	**1.98**	**5569.14**	**1.4 × 10^8^**	**6.1 ^#^**
	**10**	**3.09 × 10^12^**	**−0.35**	**10,382.5**	**4.6 × 10^8^**	**4.5 ^#^**
	**100**	**3.04 × 10^22^**	**−3.08**	**16,288.8**	**1.3 × 10^9^**	**13.2 ^#^**

*^e^ k_ave_* is the average rate constants at 800 K for class. ^#^ The uncertainty factor values of the pressure-dependent rate rule at 800 K for class in our work (The ratios of the largest rate constant to the smallest rate constant at 800 K for class).

## Data Availability

Data are contained within the article and Supplementary Materials.

## Supplementary Materials

The following supporting information can be downloaded at: https://www.mdpi.com/article/10.3390/molecules29020544/s1. The spin eigenvalues and the S^2^ computed by the Gaussian 16 package for the species. The representative IRC profiles for reactions, Lennard-Jones parameters, Potential energy profiles of internal rotations for reactions, and the Cartesian coordinates for all reactants, products, and transition states are given in the Supplemental Material-SI. The thermodynamic parameters including the enthalpies, entropies, and heat capacities in the NASA format with 14 parameters are given in the Supplemental Material-SII.
