# Supplementary material for: High-Pressure Limit and Pressure-Dependent Rate Rules for β-Scission Reaction Class of Hydroperoxyl Alkyl Hydroperoxyl Radicals (•P(OOH)2) in Normal-Alkyl Cyclohexanes Combustion
Source: Molecules. 2024 Jan 22;29(2):544. doi: 10.3390/molecules29020544 (PMC10818465; doi:10.3390/molecules29020544)
Supplement: Supplementary file 1 [file molecules-29-00544-s001.zip › Supplemental Material-I.pdf]

Supplemental Material-I

High-pressure Limit and Pressure-dependent Rate Rules for *β*-scission Reaction Class of Hydroperoxyl alkyl hydroperoxyl radicals (•P(OOH)$_2$) in Normal-alkyl Cyclohexanes Combustion

Contents:

1. The spin eigenvalues and the S^2 computed by Gaussian 16 package for the species involved in this work
2. The representative IRC profiles for reactions involved in this work
3. Lennard-Jones parameters used in this work
4. Potential energy profiles of internal rotations for reactions involved in this work
5. Cartesian coordinates for all reactants, transition states and products in the class of *β*-scission reaction

1. The spin eigenvalues and the S^2 computed by Gaussian 16 package for the species involved in this work

Table S1. Comparison between the spin eigenvalues and the S^2 computed by Gaussian 16 package for the species.

Species	Spin eigenvalues	$<S^2>$	Relative error (%)
R1	0.75	0.7538	0.5
TS1	0.75	0.7794	3.9
R2	0.75	0.7547	0.6
TS2	0.75	0.7772	3.6
R3	0.75	0.7538	0.5
TS3	0.75	0.7802	4.0
R4	0.75	0.7538	0.5
TS4	0.75	0.7789	3.9
R5	0.75	0.7548	0.6
TS5	0.75	0.7769	3.6
R6	0.75	0.7544	0.6
TS6	0.75	0.7798	4.0
R7	0.75	0.7543	0.6
TS7	0.75	0.7809	4.1
R8	0.75	0.754	0.5
TS8	0.75	0.7761	3.5
R9	0.75	0.7547	0.6
TS9	0.75	0.7766	3.5
R10	0.75	0.7545	0.6
TS10	0.75	0.7756	3.4
R11	0.75	0.754	0.5
TS11	0.75	0.7763	3.5
R12	0.75	0.754	0.5

TS12	0.75	0.7769	3.6
R13	0.75	0.7546	0.6
TS13	0.75	0.7755	3.4
R14	0.75	0.7541	0.5
TS14	0.75	0.782	4.3
R15	0.75	0.754	0.5
TS15	0.75	0.7826	4.3
R16	0.75	0.754	0.5
TS16	0.75	0.7825	4.3
R17	0.75	0.7543	0.6
TS17	0.75	0.7784	3.8
R18	0.75	0.7543	0.6
TS18	0.75	0.7809	4.1
R19	0.75	0.7544	0.6
TS19	0.75	0.7805	4.1
R20	0.75	0.7544	0.6
TS20	0.75	0.7805	4.1
R21	0.75	0.7541	0.5
TS21	0.75	0.7845	4.6
R22	0.75	0.7542	0.6
TS22	0.75	0.7767	3.6
R23	0.75	0.7545	0.6
TS23	0.75	0.7782	3.8
R24	0.75	0.7543	0.6
TS24	0.75	0.7781	3.7
R25	0.75	0.7542	0.6
TS25	0.75	0.7782	3.8
R26	0.75	0.7544	0.6
TS26	0.75	0.7784	3.8
R27	0.75	0.7541	0.5
TS27	0.75	0.7791	3.9
R28	0.75	0.7542	0.6
TS28	0.75	0.7741	3.2
R29	0.75	0.7542	0.6
TS29	0.75	0.7756	3.4
R30	0.75	0.7544	0.6
TS30	0.75	0.7749	3.3
R31	0.75	0.7541	0.5
TS31	0.75	0.775	3.3
R32	0.75	0.7541	0.5
TS32	0.75	0.7758	3.4
R33	0.75	0.7541	0.5
TS33	0.75	0.7796	3.9
R34	0.75	0.7542	0.6
TS34	0.75	0.7735	3.1
R35	0.75	0.7541	0.5
TS35	0.75	0.7755	3.4
R36	0.75	0.7544	0.6
TS36	0.75	0.7749	3.3

R37	0.75	0.7541	0.5
TS37	0.75	0.7749	3.3
R38	0.75	0.7541	0.5
TS38	0.75	0.7758	3.4
R39	0.75	0.7541	0.5
TS39	0.75	0.7796	3.9
R40	0.75	0.7542	0.6
TS40	0.75	0.7736	3.1
R41	0.75	0.7541	0.5
TS41	0.75	0.7755	3.4
R42	0.75	0.7544	0.6
TS42	0.75	0.7749	3.3
R43	0.75	0.7541	0.5
TS43	0.75	0.7749	3.3
R44	0.75	0.7541	0.5
TS44	0.75	0.7758	3.4

2. The representative IRC profiles for reactions involved in this work

Figure S1. The representative IRC profiles for reactions of R1(a), R5(b), R17(c), and R23(d).

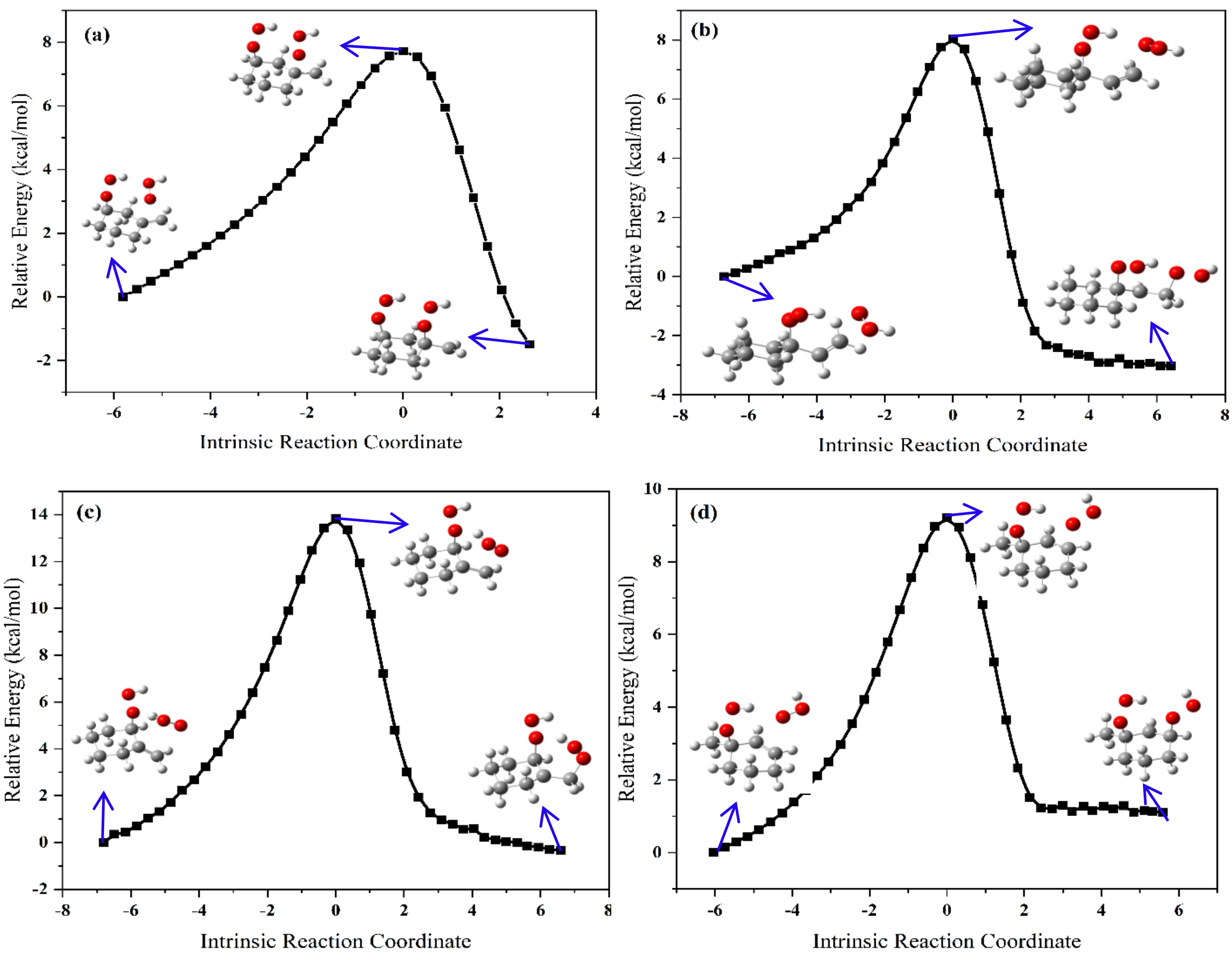

Table S2. Lennard-Jones parameters σ (Å) and ε (K) used in this work.

Reactant[a]	σ	ε
$C_7H_{11}OOHOOH$	6.72	707.81
$C_8H_{13}OOHOOH$	6.91	742.88
$C_9H_{15}OOHOOH$	7.09	776.80
$C_{10}H_{17}OOHOOH$	7.26	809.67
Ar	3.47	114

[a]Represents the reactants in the class of β-scission reaction.

[1] Wang, H.; Frenklach, M. Transport properties of polycyclic aromatic hydrocarbons for flame modeling. Combust. Flame **1994**, 96, 163-170.

4. Potential energy profiles of internal rotations for reactions involved in this work

In our study, one-dimensional (1-D) hindered internal rotors are used to treat low-frequency vibrations corresponding to torsions about a single bond for reactants, transition states and products. When dealing with the hindrance potentials of transition states, 1-D hindered rotor scans are implemented by freezing the atoms involved in the reaction centers. Since cyclohexane has a relatively stable cycle structure, the anharmonic correction corresponding to the internal rotations of the single bond on the cycle of the reactants, transition states, and products in this work are all not considered. Here, we only choose the R21 reaction as an example, and the potential energy profiles for internal rotations about C-C, C-OO and CO-OH single bonds of the reactant, the transition state, and the product in R21 are shown in Figure S2(a), Figure S2(b), and Figure S2(c).

Figure S2(a). Potential energy profiles for internal rotations of the reactant in R21 at B3LYP/CBSB7 level.

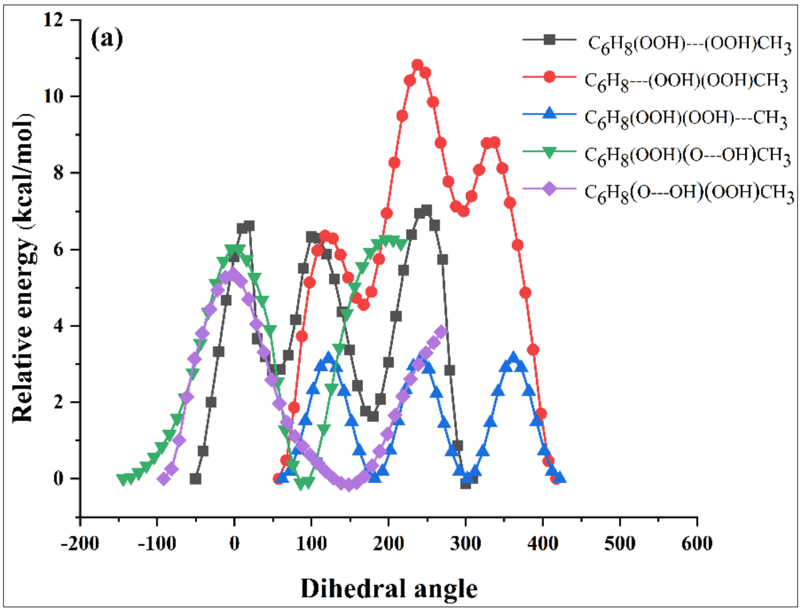

For the reactant, it can be seen from Figure S2(a) that the calculated rotational profile of $C_6H_8(OOH)$---$(OOH)CH_3$ group in reactant shows an asymmetrical three-fold potential barrier of 7.0 kcal mol^{-1}. The rotation of the C_6H_8---$(OOH)(OOH)CH_3$ group shows an asymmetrical three-fold potential barrier of 10.8 kcal mol^{-1}. The rotations of the $C_6H_8(OOH)(OOH)$---CH_3 group shows a symmetrical three-fold potential barrier of 3.1 kcal mol^{-1}. The rotations of the

$C_6H_8(OOH)(O{-}{-}{-}OH)CH_3$ group and the $C_6H_8(O{-}{-}{-}OH)(OOH)CH_3$ group in reactant show asymmetrical two-fold potential barriers of 6.3 and 5.4 kcal mol^{-1}, respectively.

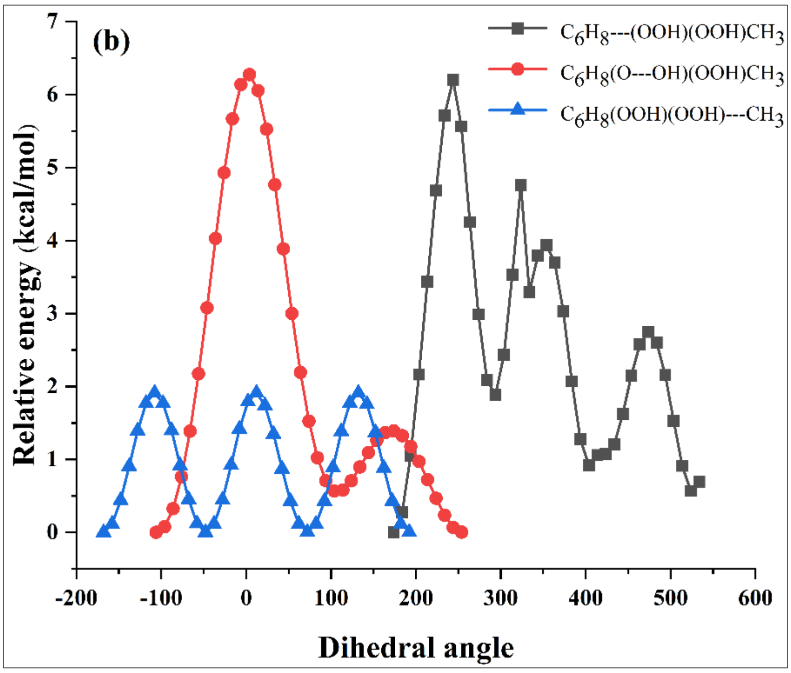

For the transition state, there are only three potential energy profiles for internal rotations about single bonds because the anharmonic correction corresponding to the single bonds involved in the reaction centers are not considered. It can be seen that from Figure S2(b) that, the calculated rotational profile of $C_6H_8{-}{-}{-}(OOH)(OOH)CH_3$ group in transition state shows an asymmetrical three-fold potential barriers of 6.2 kcal mol^{-1}, and the rotation of $C_6H_8(O{-}{-}{-}OH)(OOH)CH_3$ group in transition state shows an asymmetrical two-fold potential barriers of 6.3 kcal mol^{-1}. The rotation of the $C_6H_8(OOH)(OOH){-}{-}{-}CH_3$ group in transition state shows a symmetry three-fold barrier of 1.9 kcal mol^{-1}.

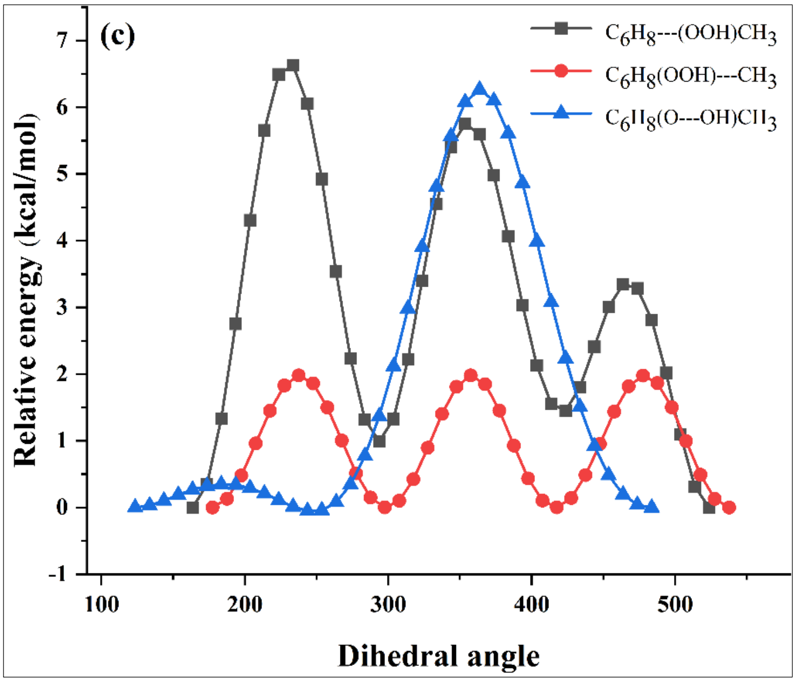

From Figure S2(c), it can be seen that, the calculated rotational profile of C_6H_8---(OOH)CH_3 group shows an asymmetrical three-fold with a barrier of 6.6 kcal mol^{-1}. The calculated rotational profile of C_6H_8(OOH)---CH_3 group shows a symmetrical three-fold with a barrier of 2.0 kcal mol^{-1}. The C_6H_8(O---OH)CH_3 group in product show an asymmetrical two-fold potential barrier of 6.3 kcal mol^{-1}.

5. Cartesian coordinates for all reactants, transition states and products in the class of *β*-scission reaction

HO_2

O	0.05528900	-0.60941600	0.00000000
H	-0.88463000	-0.87113300	0.00000000
O	0.05528900	0.71830800	0.00000000

R1

C	-1.25553900	0.55128400	0.73206500
C	0.04495700	-0.09820000	1.21924900
C	1.21602600	-0.06664000	0.21041200
C	1.39875700	1.36764600	-0.35461300
C	0.09357500	1.96505500	-0.89929900
C	-1.02247000	1.95074200	0.15355900
H	0.35781700	0.43934500	2.12097700
H	-0.14065600	-1.12852600	1.52199500
H	-1.96758700	0.59443000	1.56589300
H	1.79417700	1.99300600	0.45350600
H	2.16233700	1.33144300	-1.13551900
H	0.27343600	2.99336800	-1.22825900
H	-0.22427200	1.39606900	-1.77485800
H	-0.76027600	2.60729900	0.99153400
H	-1.95947600	2.32827700	-0.26522600
C	2.47442000	-0.57220400	0.83292800
H	3.17743300	-1.12638900	0.22616600
H	2.72066500	-0.34581300	1.86309600
O	0.91300800	-0.82556100	-0.98168100
O	0.61343800	-2.19935100	-0.62961900
H	-0.35864600	-2.16580800	-0.58469200
O	-1.88303400	-0.16376600	-0.34490700
O	-2.11595000	-1.53894800	0.10101400
H	-2.99299800	-1.68179700	-0.27886400

TS1

C	-1.28717300	0.60856900	0.68577600
C	-0.00492700	0.01114400	1.28859200
C	1.26822800	0.15861400	0.47781100
C	1.40624400	1.41549500	-0.36066600
C	0.09656400	1.88020100	-1.01011200

C	-1.03746200	1.96295800	0.01865500
H	0.16741600	0.52157400	2.24505000
H	-0.17312000	-1.03907800	1.53338900
H	-2.02723700	0.70757400	1.48918600
H	1.77774300	2.20474600	0.30757400
H	2.18220800	1.25699100	-1.11493300
H	0.25202400	2.85796200	-1.47568300
H	-0.18577600	1.18172100	-1.79892200
H	-0.79087600	2.69131500	0.80076600
H	-1.96516400	2.29698700	-0.45258100
C	2.41883300	-0.47502500	0.92970300
H	3.38430200	-0.25373500	0.49091700
H	2.36536800	-1.27669800	1.65753000
O	0.99829300	-1.04086400	-1.04086700
O	0.36994300	-2.23746300	-0.67223300
H	1.07504100	-2.89572700	-0.73858800
O	-1.86799700	-0.18881600	-0.34997700
O	-2.21762300	-1.48462700	0.19389800
H	-1.48470700	-2.01120600	-0.16882600

P1

C	-0.86953700	-0.21158200	0.76095800
C	0.49990600	-0.84996000	1.04327100
C	1.55900900	-0.42688600	0.05008300
C	1.65808700	1.06591100	-0.15744800
C	0.28380900	1.66874200	-0.50694100
C	-0.77029300	1.30198100	0.54549900
H	0.79713700	-0.52645300	2.04974700
H	0.39475000	-1.93661200	1.06619900
H	-1.55835400	-0.43468000	1.58324300
H	2.01889600	1.53458500	0.76980200
H	2.39034000	1.29408300	-0.93621900
H	0.36192000	2.75656900	-0.59584700
H	-0.03047400	1.28954800	-1.48479100
H	-0.51650100	1.76720600	1.50614200
H	-1.75561900	1.68142700	0.26461200
C	2.32758400	-1.30350700	-0.59323400
H	3.08772900	-0.98145000	-1.29741600
H	2.22355100	-2.37211600	-0.43786200
O	-1.35121100	-0.89322100	-0.40752800
O	-2.75110400	-0.52778000	-0.57581400
H	-2.72623400	-0.17228900	-1.47400000

R2

C	1.38338500	-1.00094000	-0.17488800

C	1.84891500	-2.40802200	-0.11500000
H	1.33908700	-3.14415700	0.49428600
H	2.79811400	-2.67846300	-0.56023200
O	2.32382400	-0.25448400	0.67917200
O	2.65898100	1.01776600	0.04468500
H	3.60736100	0.89784500	-0.09433100
H	1.50217300	-0.58115200	-1.17731300
C	-0.06365000	-0.80471400	0.30630200
C	-0.40615700	0.68180900	0.49828900
C	-1.07278800	-1.48675600	-0.63899800
H	-0.14656200	-1.26956400	1.29850300
C	-1.85177800	0.89854700	0.95007900
H	0.28858700	1.11591300	1.21889000
C	-2.51901300	-1.27869700	-0.16323400
H	-0.95621800	-1.06572300	-1.64421900
H	-0.85180100	-2.55575000	-0.71759400
C	-2.84829900	0.21084600	0.00780200
H	-2.03835300	1.97464900	1.00055500
H	-1.96282800	0.50433200	1.96856000
H	-3.21102100	-1.74068300	-0.87437300
H	-2.66314500	-1.79738700	0.79391800
H	-3.86855700	0.33399800	0.38388600
H	-2.80706600	0.70516000	-0.96832900
O	-0.17097000	1.31751300	-0.77590500
O	0.30037900	2.66289700	-0.53782900
H	1.24882400	2.47901100	-0.42530400

TS2

C	-1.16253500	1.22188300	-0.26714700
C	-1.73924500	2.46408800	-0.05709600
H	-1.41384500	3.10625900	0.75424000
H	-2.59886000	2.78561500	-0.63184100
O	-2.38310900	0.15328700	0.77642500
O	-2.92394800	-0.84751300	-0.04985700
H	-3.84884600	-0.58276500	-0.15116800
H	-1.42188900	0.68996500	-1.17535100
C	0.19729600	0.86879800	0.29489200
C	0.38054400	-0.65058400	0.49015300
C	1.30241000	1.44860000	-0.62447800
H	0.29985300	1.33050600	1.28536300
C	1.79456700	-1.02807100	0.93477700
H	-0.35394400	-0.99878500	1.21416000
C	2.70868100	1.07136400	-0.13657400
H	1.15486200	1.06358800	-1.63973300
H	1.19500100	2.53614400	-0.67970400

C	2.86619200	-0.44799700	0.00437500
H	1.85736800	-2.11884400	0.97636300
H	1.94774000	-0.65935300	1.95760900
H	3.45498300	1.46966700	-0.83128100
H	2.89780200	1.55058300	0.83322900
H	3.86454100	-0.69487000	0.37836600
H	2.77036500	-0.91641600	-0.98083800
O	0.06277400	-1.25950800	-0.77895700
O	-0.55633700	-2.54394400	-0.53155000
H	-1.48762400	-2.26835500	-0.47131000

P2

C	-1.36640000	0.71591000	0.94894800
C	0.12178100	0.53903300	0.62308500
C	0.47796400	-0.94661100	0.43089800
C	-0.41937000	-1.59522900	-0.64998300
C	-1.90954100	-1.41285800	-0.32699400
C	-2.26683100	0.06425900	-0.11058000
H	0.74282800	0.96683100	1.41762400
H	-1.55812100	0.26996100	1.93236700
H	-1.58092400	1.78322300	1.04267500
H	-0.18542400	-1.14587300	-1.62007300
H	-0.17766100	-2.66018000	-0.72839000
H	-2.52077800	-1.83526100	-1.13088200
H	-2.15311000	-1.98080100	0.58069300
H	-2.15221400	0.60194600	-1.05846000
H	-3.31711300	0.16355900	0.18071600
H	0.20162200	-1.41762500	1.38689300
C	1.93879500	-1.26215200	0.21587700
H	2.16292000	-2.32738900	0.26968200
C	2.93891800	-0.41698800	-0.01571000
H	3.95297100	-0.78281100	-0.13572100
H	2.78451100	0.65042800	-0.10921000
O	0.46504800	1.20176700	-0.60608100
O	0.40403700	2.63617200	-0.35614700
H	-0.24409400	2.89829500	-1.02334000

R3

C	1.98138900	-0.71325200	0.08203400
C	2.96174800	-1.70560100	0.59651400
H	3.48444600	-1.50736100	1.52587300
H	3.08682600	-2.67020500	0.12213000
O	2.57051700	0.61980100	0.07782200
O	3.54811500	0.68815500	-0.98645300
H	4.36333200	0.49064800	-0.50301300

H	1.69555100	-0.93926600	-0.94705400
C	0.73038000	-0.56758500	0.97747000
H	0.53227500	-1.52493400	1.46643200
H	0.97798400	0.14023700	1.77483300
C	-0.55983000	-0.11655000	0.25132600
C	-1.14316100	-1.24541700	-0.62352700
C	-1.60244900	0.36462400	1.27728700
C	-2.48586200	-0.87138200	-1.26852400
H	-1.27704800	-2.12307600	0.01984200
H	-0.41943300	-1.52187900	-1.39611000
C	-2.93973500	0.74057300	0.62642800
H	-1.75547600	-0.43705100	2.01095900
H	-1.18712900	1.22032400	1.81335700
C	-3.50356800	-0.40891200	-0.21821400
H	-2.87273500	-1.73267300	-1.82286000
H	-2.32191700	-0.07086600	-1.99550300
H	-3.65471000	1.02985400	1.40327600
H	-2.78740300	1.61881200	-0.00837400
H	-4.43358500	-0.10026400	-0.70608300
H	-3.75986800	-1.25241100	0.43740600
O	-0.27575700	0.91608900	-0.71359600
O	0.22161500	2.10061200	-0.04135000
H	1.17949000	1.92386100	-0.09125300

TS3

C	1.83340000	1.06363500	-0.12578500
C	2.83352700	1.93227900	0.29767700
H	3.45075100	2.45975600	-0.41899500
H	3.08740500	2.02894300	1.34791000
O	2.84304400	-0.47591700	-0.64788900
O	3.29346700	-1.18951300	0.47239900
H	4.21760300	-0.91072400	0.54472800
C	0.81682500	0.49497700	0.83683800
H	1.25021000	-0.38919600	1.31426500
H	0.62923500	1.21385900	1.64067400
C	-0.53384400	0.12109800	0.18457900
C	-1.37766400	-0.73519400	1.14431400
C	-1.31735500	1.37346900	-0.25882600
C	-2.76401500	-1.06443500	0.57631900
H	-1.48116300	-0.18635900	2.08908800
H	-0.82705100	-1.65276000	1.36047900
C	-2.70782300	1.04372400	-0.82064100
H	-1.41949700	2.02247100	0.61906800
H	-0.73211600	1.92954400	-0.99775700
C	-3.52632900	0.20241100	0.16700000

H	-3.33684300	-1.63166800	1.31691800
H	-2.64160800	-1.71392400	-0.29591500
H	-3.23334800	1.97472100	-1.05724300
H	-2.59260700	0.49417400	-1.75935300
H	-4.49232700	-0.06225400	-0.27472800
H	-3.74563400	0.80152200	1.06144600
H	1.51413900	1.14693000	-1.15924600
O	-0.31168600	-0.57325300	-1.06217100
O	0.36486300	-1.83220700	-0.81533900
H	1.29500600	-1.54971300	-0.91618700

P3

C	-1.63329900	-1.39536900	-0.92924000
C	-0.20626200	-1.27145000	-0.37568700
C	0.11224300	0.12635000	0.18626800
C	-0.97366200	0.57780600	1.17864400
C	-2.39376300	0.46301800	0.60801300
C	-2.68287500	-0.95487000	0.09894000
H	-0.06181100	-1.98659800	0.44281700
H	0.52958900	-1.52237500	-1.14308800
H	-1.72444900	-0.77881900	-1.82898800
H	-1.81322400	-2.43036400	-1.23698600
H	-0.89004700	-0.04628900	2.07606700
H	-0.76117300	1.60363200	1.49041100
H	-3.12123900	0.75148800	1.37339900
H	-2.51432600	1.17169100	-0.21989000
H	-2.67675700	-1.65100900	0.94829900
H	-3.68555800	-1.00475600	-0.33740800
C	1.51125300	0.18800700	0.83828100
H	1.65307700	1.21410300	1.19527700
H	1.51667000	-0.46362500	1.71798100
C	2.64429500	-0.17109800	-0.08032200
H	2.70260800	0.40885300	-0.99778800
C	3.55560300	-1.10928000	0.16154300
H	3.53167600	-1.70956800	1.06675600
H	4.36433100	-1.30862300	-0.53286600
O	0.09092100	0.95223900	-1.00780500
O	0.38517100	2.33321100	-0.65038600
H	-0.45930000	2.74996300	-0.86710300

R4

C	2.50144500	0.79160900	0.30757000
C	3.21232900	2.09355000	0.18769700
H	3.34172900	2.54144900	-0.79151800
H	3.56207000	2.63060600	1.05910400

O	3.14458700	-0.21218100	-0.53010900
O	4.45808700	-0.48569400	0.02713000
H	5.02016500	0.02909000	-0.56879400
H	2.52736500	0.42477300	1.33749400
C	1.04496600	0.87832500	-0.19501800
H	0.64951900	1.81636400	0.20174700
H	1.06118700	0.97487100	-1.28642400
C	0.08955800	-0.26024500	0.20609500
H	0.10571500	-0.39327300	1.29481900
C	-1.36768200	-0.03206600	-0.25663000
C	-2.27175300	-1.18563600	0.22495900
C	-1.95028500	1.32083500	0.20005200
H	-1.35850000	-0.04391800	-1.35607300
C	-3.72734300	-1.01091000	-0.22598500
H	-2.23731100	-1.22132000	1.32271500
H	-1.86961800	-2.13471500	-0.13063000
C	-3.41336700	1.49550200	-0.23828100
H	-1.88958400	1.38527400	1.29537300
H	-1.36397800	2.15405100	-0.19655900
C	-4.30097600	0.33615700	0.22955000
H	-4.33613300	-1.83461300	0.16036800
H	-3.77835400	-1.07508000	-1.32072200
H	-3.79838700	2.44833700	0.13946700
H	-3.45121400	1.55783400	-1.33354100
H	-5.32133700	0.46659000	-0.14537000
H	-4.36643400	0.34924700	1.32536500
O	0.46747500	-1.50718700	-0.38864900
O	1.47023000	-2.14887400	0.43440300
H	2.27871600	-1.80680200	0.01093400

TS4

C	-2.49283800	0.75491300	-0.71968000
C	-3.35950900	1.82727600	-0.86218000
H	-3.12839700	2.79592000	-0.43256700
H	-4.33342900	1.69945200	-1.31837700
O	-3.17378600	-0.08870000	0.87265200
O	-4.39850300	-0.71992400	0.62443100
H	-5.03331700	-0.11864300	1.03955700
H	-2.68125700	-0.12701500	-1.32151500
C	-1.06650800	0.97358500	-0.25477700
C	-0.07296900	-0.16575500	-0.54744000
H	-1.07770800	1.17389800	0.82172200
H	-0.24085400	-0.54323400	-1.56416500
C	1.40195000	0.27334000	-0.43348300
C	1.80936500	0.69816400	0.99226200

C	2.34753800	-0.82108800	-0.96367300
H	1.50589100	1.14989100	-1.08973400
C	3.28349300	1.12459700	1.05895000
H	1.63897700	-0.14272500	1.67264000
H	1.17521000	1.51832500	1.34360400
C	3.82020500	-0.39445600	-0.89246600
H	2.19527600	-1.73073200	-0.37431800
H	2.07854000	-1.06945700	-1.99652100
C	4.21653200	0.02848800	0.52821600
H	3.54809000	1.38285300	2.08948100
H	3.42483900	2.03695700	0.46404300
H	4.46123100	-1.21167000	-1.23852700
H	3.99069200	0.44579900	-1.57886300
H	5.25582700	0.37259400	0.54713400
H	4.16252800	-0.84341700	1.19242900
H	-0.69348600	1.88400000	-0.73699800
O	-0.22510500	-1.26555800	0.35352300
O	-1.38877800	-2.03662400	-0.03038300
H	-2.08282500	-1.58072600	0.48481300

P4

C	2.68153100	-1.18741300	-0.84388500
C	1.23800300	-0.69250600	-1.01985600
C	0.61725500	-0.26176300	0.32441700
C	1.50733800	0.78413000	1.02258600
C	2.94977300	0.28796600	1.19563800
C	3.56090800	-0.14271200	-0.14434000
H	1.22303500	0.15956000	-1.70788400
H	0.63434900	-1.47859300	-1.48284900
H	2.67786600	-2.11085900	-0.24968700
H	3.10512200	-1.44860700	-1.81898100
H	1.50597300	1.70365000	0.42745400
H	1.07882600	1.03885500	1.99880900
H	3.56028700	1.07004100	1.65856900
H	2.96037300	-0.56455800	1.88748700
H	3.66261300	0.73629100	-0.79374800
H	4.57088100	-0.53695700	0.00803200
H	0.57547900	-1.14731000	0.97434400
C	-0.84297800	0.22480600	0.20728000
H	-1.16778900	0.58283600	1.19220200
C	-1.81805600	-0.86387800	-0.28867800
H	-1.66739600	-0.97273700	-1.36843900
H	-1.53627400	-1.81518800	0.17620400
C	-3.27007600	-0.59176900	-0.00391300
H	-3.68695800	0.29860200	-0.46336600

C	-4.03982300	-1.35771700	0.76455600
H	-5.08348200	-1.12072700	0.93919200
H	-3.65683000	-2.25383500	1.24520400
O	-0.79484500	1.35526500	-0.67921700
O	-2.02595500	2.11454300	-0.54623400
H	-1.69292300	2.90620800	-0.10177100

R5

C	0.82846800	-1.17748600	0.45121400
H	0.88636200	-2.22220300	0.15991200
C	2.09383500	-0.54154500	0.90522400
H	1.93419600	0.39482000	1.44301300
H	2.68362900	-1.22188700	1.52808300
O	2.86446900	-0.25061400	-0.29697300
O	4.07174100	0.45306000	0.12054800
H	4.74229800	-0.19750700	-0.13020200
O	-0.14967500	0.21055700	-1.25302300
O	0.71222200	1.36308500	-1.10561500
H	1.59445000	0.94923300	-1.15357300
C	-0.40649600	-0.42163600	0.04681700
C	-1.54299800	-1.40659500	-0.28978000
C	-0.86754100	0.61886800	1.08217900
C	-2.85473800	-0.71056200	-0.67539100
H	-1.69811500	-2.03823600	0.59289300
H	-1.20481200	-2.06303900	-1.09768400
C	-2.17341600	1.31456600	0.67417800
H	-1.00457200	0.09730100	2.03754200
H	-0.08189300	1.36143300	1.22480100
C	-3.29057600	0.30348500	0.38933600
H	-3.63273200	-1.46582200	-0.82652100
H	-2.71779800	-0.19750100	-1.63145700
H	-2.47621000	2.00467300	1.46811300
H	-1.98803100	1.91932800	-0.21857600
H	-4.19795700	0.82273200	0.06511300
H	-3.54809300	-0.22659900	1.31638800

TS5

C	-0.89458200	0.82950200	0.84619900
H	-4.55484100	-0.12405900	0.65152200
C	-1.93305100	1.40142400	0.13666200
H	-2.70544800	1.94094000	0.67074600
H	-1.76715500	1.69912200	-0.89219100
O	-3.07552400	-0.01802600	-0.48077300
O	-3.74497000	-0.64979700	0.57002300
H	-0.99282900	0.70305100	1.92030900

C	0.35146000	0.28031700	0.19093400
C	1.37293000	1.42153200	-0.05099600
C	0.98544700	-0.84029800	1.03837400
C	2.70195200	0.91094700	-0.62725000
H	1.54496700	1.92285100	0.90825900
H	0.91940400	2.15917300	-0.71934700
C	2.30406600	-1.34638400	0.44386900
H	1.15888600	-0.43757400	2.04417100
H	0.26721500	-1.65583300	1.13082900
C	3.30596300	-0.20337000	0.23661800
H	3.39976900	1.75040800	-0.71304400
H	2.52750100	0.53399700	-1.63878700
H	2.72891000	-2.11049000	1.10250600
H	2.09588200	-1.83268500	-0.51387700
H	4.22246000	-0.58142500	-0.22712200
H	3.59666800	0.20802200	1.21283600
O	0.04952600	-0.16995800	-1.14649300
O	-0.72560600	-1.39411300	-1.09794300
H	-1.63391400	-1.04237100	-0.98178900

P5

C	1.68741400	0.91770500	-0.99164300
C	0.22295100	1.23865000	-0.65599600
C	-0.50458600	0.07019000	0.03011100
C	0.29744500	-0.43369000	1.24811200
C	1.75472000	-0.76294800	0.89837400
C	2.45898000	0.43093600	0.24185800
H	0.18565300	2.09446200	0.02614400
H	-0.32687900	1.52037200	-1.55823400
H	1.71759100	0.14702400	-1.76711900
H	2.16289400	1.81032100	-1.41088500
H	0.26139800	0.35057200	2.01311800
H	-0.20531000	-1.30953000	1.66363200
H	2.28530800	-1.06710500	1.80634100
H	1.77361700	-1.61947600	0.21748000
H	2.53896700	1.25055500	0.96875600
H	3.48249000	0.16149100	-0.03758400
C	-1.93195400	0.37237900	0.42730100
H	-2.48044300	-0.50266600	0.76701800
C	-2.54116800	1.55369200	0.41778600
H	-3.57280800	1.65063000	0.73790400
H	-2.04919700	2.46602400	0.10032500
O	-0.51236000	-0.94161800	-1.02105700
O	-1.05862800	-2.18705200	-0.50325500
H	-1.86818700	-2.24479600	-1.02782100

R6

C	1.20148500	-0.91920600	-0.29367900
H	0.99313200	-1.36938500	-1.25992300
C	2.55076900	-1.14077400	0.28919700
H	2.58874600	-0.88288400	1.35085300
H	2.89437400	-2.17094300	0.14931500
O	3.46325200	-0.25894900	-0.42618000
O	4.77144500	-0.38259300	0.20541000
H	5.27284800	-0.78186000	-0.51919300
C	0.29886500	0.17669200	0.17288200
H	0.35635100	0.30242000	1.26004700
O	0.76018600	1.42636000	-0.41817700
O	1.86397000	1.93318200	0.36607000
H	2.61975500	1.47171700	-0.04353000
C	-1.16311500	0.01109300	-0.26498700
C	-1.75366500	-1.31111400	0.26251300
C	-2.03236200	1.20140000	0.17970400
H	-1.16941700	-0.02578600	-1.36398700
C	-3.21954300	-1.48950400	-0.16008500
H	-1.68973900	-1.31448900	1.35915300
H	-1.15943500	-2.16232000	-0.08478000
C	-3.49667900	1.02660000	-0.24616300
H	-1.98097100	1.28511100	1.27395000
H	-1.62132000	2.12811100	-0.22344100
C	-4.08402100	-0.29528600	0.26609600
H	-3.61698400	-2.41780000	0.26282600
H	-3.26753700	-1.59916500	-1.25118300
H	-4.09154500	1.87149900	0.11495500
H	-3.55931600	1.04988600	-1.34189700
H	-5.10960400	-0.42328200	-0.09499200
H	-4.14056800	-0.26423700	1.36197600

TS6

C	1.25251600	-0.89672500	0.09355100
H	4.99704500	-1.11792900	0.70152400
C	2.31945800	-1.30926500	-0.68609800
H	2.86249500	-2.20560900	-0.41134500
H	2.33512100	-1.05906300	-1.74148300
O	3.75233400	-0.12024900	-0.27115300
O	4.33609400	-0.45527400	0.95382600
C	0.32021200	0.21900800	-0.27928800
H	0.31213300	0.37768700	-1.36332500
C	-1.11179700	0.02474800	0.24136300
C	-2.01411000	1.21756100	-0.12244800

C	-1.72231600	-1.29148600	-0.27774800
H	-1.04942400	-0.03593400	1.33751700
C	-3.44834100	1.02051300	0.38763400
H	-2.03012700	1.32628200	-1.21555800
H	-1.58504400	2.13798300	0.27654200
C	-3.15882400	-1.49094400	0.22786900
H	-1.72352000	-1.27284900	-1.37609400
H	-1.10201200	-2.14441200	0.01645600
C	-4.05568700	-0.29473900	-0.11857900
H	-4.07033200	1.86805500	0.08281900
H	-3.44422100	1.01849400	1.48535400
H	-3.57477700	-2.41296600	-0.19095200
H	-3.14017300	-1.62476700	1.31713600
H	-5.05674800	-0.43957300	0.30029200
H	-4.17824500	-0.23973900	-1.20812800
H	1.18890300	-1.25134000	1.11896400
O	0.81458300	1.43236100	0.33238200
O	1.88750000	1.95261500	-0.48187400
H	2.64817600	1.40801500	-0.19272300

P6

C	-2.25544000	1.34847300	-0.49065900
C	-0.75280700	1.04004600	-0.55878300
C	-0.32397000	0.07578800	0.56331400
C	-1.17215400	-1.20880100	0.53412700
C	-2.67552900	-0.90188200	0.59419100
C	-3.09958400	0.06701100	-0.51800400
H	-0.51671400	0.58547700	-1.52718000
H	-0.17154800	1.96420700	-0.49884500
H	-2.47105500	1.90258000	0.43261300
H	-2.53764600	2.00608800	-1.31914700
H	-0.94733000	-1.75943600	-0.38601500
H	-0.88858500	-1.85935600	1.36958500
H	-3.24921100	-1.83207300	0.52775500
H	-2.91563700	-0.45813300	1.56938000
H	-2.97571500	-0.42493900	-1.49137600
H	-4.16315300	0.30934000	-0.42382200
H	-0.50807700	0.57635700	1.52493700
C	1.19128600	-0.23723600	0.55072900
H	1.38272800	-1.01455700	1.30369800
C	2.04408000	0.96530500	0.86837300
H	1.88875900	1.37560400	1.86494200
C	2.92971800	1.52160300	0.05057200
H	3.11069300	1.11862000	-0.93841900
H	3.50734500	2.38751400	0.35318700

O	1.47365000	-0.79840000	-0.73428100
O	2.73956500	-1.51355800	-0.64724100
H	2.43582100	-2.40347800	-0.87227400

R7

C	2.04040900	-1.07493400	0.56369800
H	2.05039200	-2.12404700	0.28315200
C	3.32812500	-0.45683100	0.97624200
H	3.17955800	0.47799200	1.52361000
H	3.93810400	-1.14004700	1.57570300
O	4.05694700	-0.15425600	-0.24808000
O	5.27387500	0.55132600	0.13513000
H	5.93883200	-0.09470900	-0.14116000
C	0.84658100	-0.25876300	0.18561400
H	0.73565600	0.59911600	0.85542700
O	1.03846500	0.28004600	-1.15372100
O	1.88186600	1.45093100	-1.06842400
H	2.77070000	1.05081600	-1.10993500
C	-0.43913900	-1.08398900	0.11235600
H	-0.54538200	-1.62838400	1.05888300
H	-0.30198300	-1.84136700	-0.66879700
C	-1.71954100	-0.28270900	-0.17510600
C	-2.88241200	-1.23184700	-0.51979200
C	-2.11894200	0.64170400	0.99048900
H	-1.52434400	0.34430600	-1.05326600
C	-4.18637600	-0.47544300	-0.80936400
H	-3.04348400	-1.92147200	0.32102600
H	-2.60922100	-1.85124200	-1.38125200
C	-3.42130900	1.40430400	0.70429700
H	-2.24732400	0.03446500	1.89803400
H	-1.31844200	1.35705000	1.20313300
C	-4.57087100	0.45119100	0.35173900
H	-4.99470700	-1.18531800	-1.01342000
H	-4.05927100	0.12250500	-1.72082500
H	-3.69031000	2.02131300	1.56788000
H	-3.25532600	2.09525200	-0.13189600
H	-5.47247900	1.01974900	0.10152300
H	-4.81934100	-0.15645200	1.23190700

TS7

C	-2.10971600	-1.13781900	-0.02775000
H	-5.78891200	-0.32328600	-0.48119900
C	-3.19675400	-1.15784700	0.82932600
H	-3.98555700	-1.88310200	0.66925600
O	-4.25924500	0.35121000	0.35265100

O	-4.98422300	0.10134800	-0.81561300
H	-3.07273000	-0.82165400	1.85323900
C	-0.87430200	-0.31447100	0.19971500
H	-0.74403400	-0.09767100	1.26519100
C	0.38111400	-0.97183100	-0.38333800
H	0.34585800	-0.86391800	-1.47468000
H	0.30970600	-2.04671200	-0.17813200
C	1.72737800	-0.46066000	0.15959000
C	2.86869100	-1.39542700	-0.28703500
C	2.04623100	0.99379200	-0.23677700
H	1.67807900	-0.50551100	1.25827200
C	4.23709400	-0.94412600	0.24223600
H	2.89611700	-1.42009300	-1.38515600
H	2.66012800	-2.42035200	0.04106800
C	3.41300400	1.44794100	0.29593500
H	2.04045200	1.06618000	-1.33287500
H	1.25885400	1.66333000	0.11288600
C	4.54182800	0.50843500	-0.14731100
H	5.02101000	-1.61192700	-0.13006000
H	4.24679000	-1.03343500	1.33643600
H	3.61801200	2.47083800	-0.03593700
H	3.38079000	1.48011600	1.39310500
H	5.49596500	0.82503500	0.28671900
H	4.65790600	0.57272600	-1.23712400
H	-2.21137500	-1.58859400	-1.01141600
O	-1.03939100	0.94930000	-0.48358700
O	-1.87244800	1.80371000	0.33061800
H	-2.77200500	1.48555700	0.11030900
P7			
C	2.74750700	1.24610900	0.63903000
C	1.39068300	0.62438900	1.00253600
C	0.81537900	-0.22573900	-0.14560100
C	1.84052100	-1.28976300	-0.58026200
C	3.19838700	-0.67499800	-0.94756800
C	3.75881000	0.18031900	0.19655200
H	1.51141400	-0.01316700	1.89004100
H	0.68989700	1.41783300	1.28082400
H	2.60433400	1.96842500	-0.17464400
H	3.13945300	1.81179500	1.49061800
H	1.97893300	-2.00962200	0.23885300
H	1.44416600	-1.85806000	-1.42884500
H	3.90793600	-1.46493500	-1.21485000
H	3.07864000	-0.04848300	-1.84069500
H	3.99641100	-0.46851800	1.04986300

H	4.69912100	0.65193600	-0.10699700
H	0.63491100	0.43667400	-1.00103700
C	-0.52451000	-0.88815900	0.21704900
H	-0.79819000	-1.61930700	-0.55186600
H	-0.42102100	-1.45054900	1.15298500
C	-1.71166900	0.07456300	0.36942600
H	-1.44578700	0.90805300	1.02809000
C	-2.93467100	-0.62213000	0.92062800
H	-2.94147400	-0.76824700	1.99888700
C	-3.95231300	-1.06101800	0.18525800
H	-4.80012300	-1.56588100	0.63284300
H	-3.95843200	-0.94234000	-0.89376500
O	-1.94251400	0.61121400	-0.93868300
O	-2.73909100	1.81203900	-0.80262500
H	-3.63609500	1.44692000	-0.76213000

R8

C	2.74470700	1.04580800	-0.80238300
C	1.32239600	1.40057800	-0.34227000
C	0.50928700	0.16108100	0.12493300
C	1.30906400	-0.63637700	1.17693100
C	2.72265400	-0.98974400	0.69938900
C	3.50541100	0.25801900	0.27136500
H	1.36546900	2.09635000	0.50284200
H	0.77124800	1.90173600	-1.14316000
H	2.68672700	0.45256300	-1.71920400
H	3.27939100	1.96839700	-1.05100700
H	1.36251600	-0.02882700	2.08844200
H	0.75256200	-1.54237400	1.42522500
H	3.25323900	-1.51702600	1.49845800
H	2.64912400	-1.68298600	-0.14412400
H	3.67313500	0.90112200	1.14588600
H	4.49476500	-0.02496300	-0.10124400
C	-0.83455900	0.57582400	0.64991600
H	-0.93043100	0.75959300	1.71533900
C	-2.02899800	0.79454700	-0.21776400
O	0.37759000	-0.59524600	-1.10151300
O	-0.31138600	-1.85099400	-0.84501700
H	-1.20323300	-1.60134900	-1.15123800
O	-2.65687800	-0.47368200	-0.62138700
O	-3.06531000	-1.19783000	0.56690300
H	-2.25661100	-1.69821300	0.76474300
C	-3.07247400	1.71716800	0.39601500
H	-2.64012200	2.70147300	0.59407500

H	-3.91716400	1.83591000	-0.28501300
H	-3.43967000	1.30014300	1.33521700
H	-1.71799200	1.15904100	-1.20393300

TS8

C	-0.77660300	0.68693700	0.62045000
H	-4.25148300	-0.81067600	0.47628500
C	-1.84893400	1.00571600	-0.19887800
H	-1.64471400	1.07902300	-1.26266400
O	-2.70208900	-0.70945500	-0.56196300
O	-3.37459800	-1.21413900	0.55656000
H	-0.90526200	0.74928600	1.69828700
C	0.55843000	0.19769500	0.10840000
C	1.43295000	1.40171900	-0.32875500
C	1.29322800	-0.64154900	1.17222700
C	2.84370700	0.97903700	-0.76524500
H	1.49176700	2.09549700	0.51768300
H	0.92008900	1.92904900	-1.13852700
C	2.69356200	-1.06586100	0.71716400
H	1.36449100	-0.03350400	2.08281600
H	0.68457400	-1.51530700	1.40771900
C	3.54625600	0.14333900	0.31218000
H	3.42915200	1.87471500	-0.99796400
H	2.76853000	0.39601400	-1.68722700
H	3.18266000	-1.62417500	1.52172400
H	2.59674600	-1.75013300	-0.13102000
H	4.52537400	-0.18643800	-0.04922700
H	3.73399600	0.76912400	1.19535600
O	0.38749800	-0.53548700	-1.12290700
O	-0.19766200	-1.83433900	-0.85647400
H	-1.15148800	-1.60522300	-0.81193400
C	-3.02371600	1.78928600	0.31513900
H	-3.87939500	1.69689600	-0.35691900
H	-3.31323100	1.45315000	1.31383000
H	-2.77027700	2.85215600	0.38396200

P8

C	-1.91615300	-1.18593200	-0.96967300
C	-0.39415000	-1.14833200	-0.76313700
C	0.08244200	0.12312100	-0.03973600
C	-0.71208000	0.33782000	1.26527000
C	-2.23034400	0.30886700	1.04631600
C	-2.67328000	-0.98335400	0.34914600
H	-0.08833200	-2.00651700	-0.15542400
H	0.12484600	-1.22911600	-1.72229400
H	-2.20215300	-0.40240500	-1.67713700

H	-2.19246500	-2.14265200	-1.42481000
H	-0.41728800	-0.45346800	1.96451900
H	-0.40684500	1.28754600	1.70980600
H	-2.73837900	0.41605300	2.01016400
H	-2.51937100	1.17088400	0.43718400
H	-2.48446400	-1.83796100	1.01299000
H	-3.75207600	-0.96506100	0.16407400
C	1.56836700	0.16901600	0.23481500
H	1.90229300	1.10740600	0.67384600
C	2.46564300	-0.79014300	0.01644400
H	2.14941600	-1.73794000	-0.41265900
O	-0.25736400	1.16375100	-1.00730000
O	0.02709100	2.47387300	-0.44450700
H	0.78857800	2.72777200	-0.98279500
C	3.92997100	-0.67587000	0.32711700
H	4.53580800	-0.82135400	-0.57430900
H	4.17711900	0.30042400	0.75062400
H	4.24299600	-1.44575400	1.04130000

R9

C	1.35049800	-0.27502500	-0.01906400
C	2.31918600	-1.39021300	0.14565600
H	2.07725500	-2.18515200	0.84431600
O	1.88172800	0.81145700	0.82734100
O	1.68598400	2.10465300	0.17225800
H	2.60114900	2.33875800	-0.03020400
H	1.36292900	0.11534700	-1.04114800
C	-0.08614300	-0.63730000	0.39053000
C	-0.99298500	0.60168300	0.46677800
C	-0.69164900	-1.70630400	-0.54138900
H	-0.04255800	-1.04767800	1.40887700
C	-2.43238600	0.25478600	0.85283200
H	-0.56722000	1.31016400	1.17897400
C	-2.12998500	-2.06067300	-0.13286400
H	-0.68883900	-1.32307500	-1.56828700
H	-0.06633000	-2.60434500	-0.53829100
C	-3.02441100	-0.81453100	-0.07448800
H	-3.02663700	1.17205100	0.82317000
H	-2.44033300	-0.10076400	1.89138100
H	-2.54347800	-2.79137400	-0.83518800
H	-2.11660600	-2.54743700	0.85147900
H	-4.03186300	-1.08451200	0.25686900
H	-3.12258400	-0.39195100	-1.07979600
O	-0.95174900	1.21280500	-0.84015600

O	-1.06443900	2.64467700	-0.68046200
H	-0.13183500	2.85480600	-0.50071200
C	3.70210800	-1.31243900	-0.40023200
H	4.38875300	-0.82481300	0.30837200
H	4.11248200	-2.30655400	-0.60042800
H	3.73812200	-0.73210300	-1.32779100

TS9

C	1.23040000	-0.59620100	-0.08801500
C	2.19824100	-1.53616600	0.23326100
H	2.01686200	-2.20032300	1.07586900
O	1.92846700	0.91030800	0.91956600
O	2.07588100	2.01397200	0.05385600
H	3.03274500	2.07951600	-0.07002300
H	1.34359100	-0.05427300	-1.02113400
C	-0.19895400	-0.73007900	0.38989400
C	-0.92609600	0.62827700	0.46217100
C	-0.96604100	-1.72188000	-0.52120700
H	-0.19251400	-1.13825100	1.40879200
C	-2.40460900	0.49406600	0.83070900
H	-0.40688300	1.26044500	1.18038200
C	-2.44059100	-1.85182800	-0.11207000
H	-0.90579800	-1.36868800	-1.55675900
H	-0.47349000	-2.69874800	-0.49082100
C	-3.14054800	-0.48694800	-0.08946100
H	-2.85666300	1.48868500	0.78792800
H	-2.47485200	0.15477500	1.87261100
H	-2.95239700	-2.53181500	-0.80059000
H	-2.50216100	-2.31115200	0.88356300
H	-4.18108500	-0.59743600	0.23101800
H	-3.16166100	-0.07260000	-1.10290000
O	-0.77703200	1.23304600	-0.83946000
O	-0.68220700	2.66731000	-0.67231400
H	0.27850800	2.75239400	-0.53935900
C	3.54181700	-1.60809000	-0.41050100
H	4.33478600	-1.36033700	0.30653300
H	3.75647200	-2.62204800	-0.76865000
H	3.62194800	-0.92414400	-1.25832600

P9

C	-1.90937000	0.53095000	0.92658400
C	-0.40443100	0.57254100	0.63385700
C	0.16981300	-0.84567400	0.45770200
C	-0.60006000	-1.61826300	-0.63992200
C	-2.10786700	-1.65572200	-0.35130300
C	-2.68197800	-0.24654600	-0.14889900

H	0.12950100	1.08873600	1.43914400
H	-2.05537800	0.06298400	1.90756600
H	-2.27966000	1.55557300	1.01132500
H	-0.41216500	-1.14051500	-1.60653300
H	-0.20364900	-2.63650400	-0.70882700
H	-2.63296600	-2.16361300	-1.16661100
H	-2.28641900	-2.25224700	0.55320900
H	-2.62686000	0.30095400	-1.09655800
H	-3.74158100	-0.30127000	0.11976200
H	-0.06220900	-1.34810500	1.40961000
C	1.66625500	-0.94810800	0.28066500
H	2.04333800	-1.96931700	0.35543400
C	2.54261700	0.02939800	0.05670900
H	2.18656700	1.04962600	-0.04876600
O	-0.13589000	1.27722000	-0.59145000
O	-0.41432700	2.68740100	-0.35208200
H	-1.09467900	2.84374900	-1.02040100
C	4.02294300	-0.17631100	-0.08607000
H	4.57568900	0.39404800	0.66924400
H	4.29751800	-1.22929000	0.01667300
H	4.37714700	0.17464100	-1.06195700

R10

C	-1.04253400	0.62336300	-0.34798200
H	-0.87385900	1.05684300	-1.33059600
C	-2.40005800	0.79321700	0.24493100
H	-2.39414200	0.51899800	1.30463100
O	-3.22425700	-0.21148600	-0.43823800
O	-4.49573400	-0.32039500	0.27591400
H	-5.10424800	-0.02145900	-0.41366500
C	-0.05436900	-0.38212400	0.14780200
H	-0.11412000	-0.49484000	1.23623100
O	-0.39176300	-1.67679800	-0.42706100
O	-1.46125200	-2.26489500	0.34705200
H	-2.24399400	-1.84595600	-0.05854900
C	-2.98687200	2.18459800	0.04865000
H	-4.00544100	2.22359400	0.43859000
H	-2.38959900	2.92706700	0.58356900
H	-3.00012400	2.44806800	-1.01238000
C	1.39377900	-0.09258400	-0.27465600
C	1.85725900	1.28401900	0.24023000
C	2.35866800	-1.19480500	0.19890500
H	1.41204700	-0.07078800	-1.37391400
C	3.30795100	1.58630300	-0.16402300

H	1.77625100	1.29751700	1.33572400
H	1.19525200	2.07365000	-0.12918400
C	3.80806900	-0.89587300	-0.20889900
H	2.29828500	-1.26889900	1.29340400
H	2.03775600	-2.15954300	-0.19673900
C	4.26798800	0.47950700	0.29235700
H	3.61492300	2.55202100	0.25044300
H	3.36300900	1.68472400	-1.25587100
H	4.47002500	-1.67975600	0.17259300
H	3.88919100	-0.92785500	-1.30322200
H	5.28382900	0.69312900	-0.05561500
H	4.30972300	0.46834400	1.38930600

TS10

C	-1.09560600	0.59882700	0.18111600
H	-4.82335600	0.32119600	0.79296800
C	-2.19075500	0.99475700	-0.57975500
H	-2.16676300	0.74643600	-1.63757600
O	-3.45989700	-0.41550800	-0.25469400
O	-4.08491100	-0.27385100	0.99089200
C	-0.07574800	-0.41196500	-0.25465900
H	-0.06940500	-0.51706700	-1.34514300
C	1.34218500	-0.10680600	0.25556100
C	2.34536100	-1.19021600	-0.17934900
C	1.81759500	1.28423600	-0.20589800
H	1.29486600	-0.10521400	1.35417100
C	3.76447800	-0.88357200	0.31901400
H	2.35077000	-1.24472200	-1.27663600
H	2.01122700	-2.16475000	0.17972500
C	3.23893300	1.59397300	0.28727900
H	1.79881300	1.31900400	-1.30376300
H	1.12655000	2.05984000	0.14021900
C	4.23668000	0.50545900	-0.13083300
H	4.45693200	-1.65336200	-0.03610100
H	3.78123100	-0.93419000	1.41546600
H	3.55885600	2.57003600	-0.09170100
H	3.22910100	1.67334500	1.38200500
H	5.22780000	0.72392600	0.27975400
H	4.34266600	0.51407900	-1.22347800
H	-1.05482400	0.90181000	1.22530700
O	-0.43909800	-1.69657900	0.29937400
O	-1.44295900	-2.29505800	-0.54850200
H	-2.25457100	-1.82321500	-0.26661400
C	-3.00972000	2.20005100	-0.21200000
H	-2.48046000	3.11490100	-0.49639900

H	-3.96787600	2.19793900	-0.73665200
H	-3.18704800	2.23951800	0.86502800

P10

C	2.28945800	-1.62653800	-0.54859600
C	0.89591800	-0.98595000	-0.62148100
C	0.66216300	-0.00976700	0.54678300
C	1.77609300	1.05145200	0.60312400
C	3.17115300	0.41299000	0.66831900
C	3.39863600	-0.56740700	-0.49052200
H	0.79351100	-0.44068900	-1.56620200
H	0.12151900	-1.75784800	-0.62166500
H	2.35081000	-2.26197200	0.34496700
H	2.44078800	-2.28684100	-1.40868700
H	1.70507000	1.68566900	-0.28747400
H	1.62195300	1.70489100	1.46971100
H	3.93957100	1.19297600	0.66320300
H	3.27961500	-0.12334100	1.62026600
H	3.41420900	-0.01027100	-1.43615900
H	4.37843100	-1.04660300	-0.39439100
H	0.70395600	-0.58862500	1.48081200
C	-0.74530800	0.63307700	0.53109800
H	-0.77697700	1.39321500	1.32445700
C	-1.85514300	-0.36112000	0.76334100
H	-1.83140900	-0.84388400	1.74028500
C	-2.81906200	-0.66467100	-0.10092900
H	-2.82900400	-0.15630700	-1.06108100
O	-0.85588900	1.31441600	-0.72345700
O	-1.93731000	2.28452900	-0.61820900
H	-1.43756100	3.09779100	-0.77186000
C	-3.92025400	-1.65167000	0.15116000
H	-3.83314400	-2.11495500	1.13712100
H	-4.90183700	-1.16866800	0.08741800
H	-3.91583200	-2.44846800	-0.60117000

R11

C	-1.81210400	0.16616500	0.09835100
C	-2.95587700	0.96502300	0.62410400
H	-3.36808600	0.66123200	1.58299600
O	-2.15578500	-1.25056800	0.07475600
O	-3.12526500	-1.47186700	-0.97559800
H	-3.95458600	-1.38653400	-0.48298500
H	-1.57673300	0.45453100	-0.92946500
C	-0.55020300	0.22956300	0.98631800
H	-0.51884400	1.20169100	1.48602000

H	-0.66528200	-0.51865600	1.77687200
C	0.79721100	0.01861500	0.25412600
C	1.17357100	1.24220600	-0.60690900
C	1.91099500	-0.28689800	1.27264800
C	2.55846500	1.11418100	-1.25837100
H	1.15617700	2.12178100	0.04759300
H	0.41002400	1.39867800	-1.37466900
C	3.29111900	-0.41765900	0.61582600
H	1.92525700	0.52019600	2.01619900
H	1.65182400	-1.20801700	1.79847000
C	3.64454900	0.82212600	-0.21536900
H	2.78872000	2.03598200	-1.80255900
H	2.53287700	0.30605000	-1.99485800
H	4.04812500	-0.58791400	1.38807500
H	3.29108500	-1.30135600	-0.02961200
H	4.61225600	0.68500100	-0.70832200
H	3.75338800	1.68929100	0.45041600
O	0.69507600	-1.03550000	-0.72415600
O	0.41156200	-2.29673500	-0.06746300
H	-0.56278500	-2.28652800	-0.11346600
C	-3.35247600	2.27228300	0.03172700
H	-4.35784100	2.56825000	0.34168700
H	-2.67760700	3.08985600	0.33549300
H	-3.32416800	2.24018500	-1.06291300

TS11

C	1.67986000	0.58597500	0.04986900
C	2.79228500	1.23628300	0.57632800
H	2.96376100	1.18325200	1.64963000
O	2.43010100	-1.06298300	-0.60343500
O	2.71255900	-1.94103500	0.45850000
H	3.66447200	-1.82198000	0.58504600
C	0.54639800	0.11219500	0.92865400
H	0.80410600	-0.87614600	1.32178600
H	0.45055400	0.77045100	1.79816300
C	-0.82140000	0.04105300	0.21175100
C	-1.83456000	-0.74779500	1.05894500
C	-1.36503500	1.44628500	-0.11743500
C	-3.23293600	-0.77760900	0.42913700
H	-1.88168300	-0.28484400	2.05297000
H	-1.45482300	-1.76277000	1.19084800
C	-2.76763900	1.41608000	-0.74159300
H	-1.39146200	2.01437100	0.82012700
H	-0.66541500	1.96297900	-0.78175900
C	-3.75460500	0.63486500	0.13529100

H	-3.92214800	-1.30787100	1.09417600
H	-3.18679200	-1.35010100	-0.50239000
H	-3.11970600	2.44183500	-0.89305500
H	-2.70886400	0.94900000	-1.72893600
H	-4.73303200	0.58401100	-0.35309500
H	-3.90551300	1.17246400	1.08152300
H	1.42243000	0.81148100	-0.98063800
O	-0.67072400	-0.55636100	-1.09452200
O	-0.22200800	-1.92929300	-0.96355700
H	0.74719600	-1.79800700	-1.00303400
C	3.84364200	1.89112800	-0.25327300
H	4.80495700	1.36875900	-0.16147000
H	3.57228400	1.90896800	-1.31027800
H	4.02419600	2.92076300	0.07846000

P11

C	-1.77079200	-1.61506400	-0.88046100
C	-0.42090900	-1.24039500	-0.25151600
C	-0.35201600	0.22285400	0.22293400
C	-1.56090500	0.56708600	1.11076400
C	-2.90435600	0.20162100	0.46483400
C	-2.94476800	-1.27351100	0.04583800
H	-0.23193200	-1.87082300	0.62551700
H	0.39723500	-1.42479800	-0.95137400
H	-1.88704200	-1.07880500	-1.82750900
H	-1.77429300	-2.68311300	-1.12071700
H	-1.45066600	0.02238700	2.05580600
H	-1.52463100	1.63123100	1.35763700
H	-3.71939300	0.42513900	1.16060900
H	-3.06822700	0.82879200	-0.41947400
H	-2.89737200	-1.90452500	0.94344700
H	-3.89539200	-1.50283700	-0.44619600
C	0.97312900	0.53791700	0.95202400
H	0.93170700	1.59200300	1.24815000
H	1.01118000	-0.05293900	1.87310200
C	2.21022600	0.30175900	0.13281100
H	2.25904400	0.84148000	-0.81063800
C	3.22430300	-0.48626100	0.48668400
H	3.16649500	-1.02269100	1.43404900
O	-0.41141000	0.95640000	-1.02877200
O	-0.34896900	2.38644400	-0.75944900
H	-1.22984800	2.65464600	-1.05252500
C	4.47439100	-0.70093000	-0.31633900
H	4.45101800	-0.13477700	-1.25052100
H	5.36441600	-0.39363300	0.24468300

H	4.61091700	-1.75994300	-0.56370900

R12

C	-2.88548000	-1.35259300	-0.76205700
C	-1.44563700	-1.47667700	-0.24055400
C	-0.82211800	-0.10766100	0.15033500
C	-1.75760900	0.64791800	1.11804800
C	-3.18808900	0.77058700	0.57969700
C	-3.78185700	-0.59945900	0.22865900
H	-1.42513200	-2.10852300	0.65415900
H	-0.80472100	-1.95527700	-0.98652900
H	-2.87493700	-0.82512400	-1.71993800
H	-3.28322000	-2.35479800	-0.95250300
H	-1.76138000	0.10639000	2.07176600
H	-1.33615100	1.63669400	1.31011200
H	-3.81209100	1.27736300	1.32257100
H	-3.17814000	1.40456900	-0.31220300
H	-3.89332900	-1.19439300	1.14524800
H	-4.78628800	-0.48089800	-0.18946000
C	0.54612400	-0.29645400	0.73831800
H	0.62776900	-0.39009600	1.81662200
C	1.78804700	-0.42055400	-0.07956900
O	-0.75058000	0.56889400	-1.12673300
O	-0.24124000	1.92054700	-0.94987200
H	0.68447300	1.77128100	-1.21829400
O	2.25678700	0.88751700	-0.56655100
O	2.52333200	1.74794600	0.57046100
H	1.64576200	2.13849100	0.71562400
C	2.91793800	-1.16001700	0.63619600
H	2.52467800	-2.12741600	0.96818000
H	3.17396900	-0.59568500	1.53757600
H	1.56420400	-0.89662300	-1.04259800
C	4.16076600	-1.36400200	-0.23210200
H	4.55630000	-0.40612600	-0.57372900
H	4.94575500	-1.87701500	0.32896900
H	3.93358100	-1.96856500	-1.11584000

TS12

C	0.50437000	-0.40966000	0.74259200
H	3.76776600	1.54072700	0.54553700
C	1.64111500	-0.67237000	-0.00570400
H	1.49519700	-0.86526600	-1.06498900
O	2.28499800	1.11673500	-0.50953300
O	2.83495200	1.80059500	0.57989500
H	0.59045700	-0.35969200	1.82549700

C	-0.85765700	-0.14345100	0.14392400
C	-1.55877100	-1.48571500	-0.19255700
C	-1.73391600	0.69596500	1.09460800
C	-2.99143000	-1.28971800	-0.71060800
H	-1.56685900	-2.09511900	0.71839800
H	-0.95176400	-2.02059600	-0.92892000
C	-3.15559500	0.89313300	0.55795100
H	-1.76768200	0.17571100	2.06007700
H	-1.24794000	1.65859800	1.25734200
C	-3.83571300	-0.44799100	0.25443400
H	-3.45207800	-2.27079600	-0.86703700
H	-2.95025900	-0.79527600	-1.68500500
H	-3.74259000	1.46141500	1.28647200
H	-3.10819600	1.49757500	-0.35276700
H	-4.83193300	-0.28164500	-0.16737400
H	-3.98204900	-1.00281700	1.19124400
O	-0.72709500	0.48073000	-1.15046700
O	-0.32353600	1.86465300	-1.00212700
H	0.64804800	1.76690200	-0.89768600
C	2.88471600	-1.24689800	0.62669100
H	3.17439100	-0.60313000	1.46541000
H	2.62689900	-2.21439800	1.07376200
C	4.05257400	-1.43319800	-0.34557700
H	4.93345200	-1.82343400	0.16994400
H	3.79187900	-2.14023000	-1.13825200
H	4.32055900	-0.49082900	-0.82832400

P12

C	-2.15721200	-1.35625100	-0.97959900
C	-0.66509500	-1.17447900	-0.66213400
C	-0.35444400	0.17426200	0.00973600
C	-1.26531000	0.39526400	1.23515200
C	-2.75383300	0.22035100	0.90638100
C	-3.03141700	-1.14403500	0.26294100
H	-0.34052300	-1.96521200	0.02246500
H	-0.06488000	-1.26688900	-1.57152600
H	-2.44898800	-0.64313100	-1.75581200
H	-2.31660200	-2.35829500	-1.39107300
H	-0.96235800	-0.32434100	2.00476500
H	-1.07622200	1.39267000	1.63787500
H	-3.34384600	0.33820300	1.82103200
H	-3.06423400	1.01704600	0.22330400
H	-2.82642600	-1.93887200	0.99294200
H	-4.08984300	-1.23060200	-0.00281200
C	1.09634200	0.36596300	0.38889300

H	1.31916500	1.36054900	0.77109100
C	2.08047400	-0.52889700	0.32323100
H	1.87812800	-1.53143900	-0.04905800
O	-0.70062000	1.12219400	-1.04680300
O	-0.57502400	2.48312500	-0.55064100
H	0.19803900	2.77521600	-1.05171300
C	3.50623900	-0.26764700	0.72449500
H	3.59648300	0.74878700	1.12088900
H	3.77902200	-0.94687000	1.54263200
C	4.49739100	-0.46880500	-0.43344400
H	5.52607500	-0.30149100	-0.10309300
H	4.43701200	-1.48546800	-0.83325000
H	4.28634200	0.22323700	-1.25302600

R13

C	1.00439900	0.24099000	0.07096700
C	2.24673600	-0.53743300	0.31543300
H	2.21972200	-1.33723100	1.05098300
O	1.15809800	1.46158000	0.88775000
O	0.62415300	2.61980800	0.17220200
H	1.44095900	3.09658000	-0.02519500
H	0.94804000	0.58562000	-0.96578100
C	-0.28214300	-0.51182700	0.44730700
C	-1.51324800	0.40881800	0.43343800
C	-0.50488400	-1.74315100	-0.45377700
H	-0.16862800	-0.85569900	1.48468800
C	-2.80613800	-0.33125300	0.78313200
H	-1.34705600	1.23456500	1.12695300
C	-1.79556500	-2.48893500	-0.08085200
H	-0.56422700	-1.41226300	-1.49708000
H	0.35389600	-2.41787100	-0.38535600
C	-3.01570700	-1.55824400	-0.11372900
H	-3.63953700	0.37018100	0.68906200
H	-2.76036500	-0.63760300	1.83628900
H	-1.94401100	-3.33274900	-0.76210700
H	-1.68851400	-2.91576200	0.92533400
H	-3.91578600	-2.09977600	0.19328800
H	-3.18395300	-1.21772500	-1.14067800
O	-1.58959600	0.96089600	-0.89806800
O	-2.11891600	2.30261800	-0.80838400
H	-1.29674500	2.78058500	-0.60379700
C	3.57381600	-0.09397400	-0.20107700
H	3.99283700	0.66615500	0.47876000
H	3.44086300	0.41987000	-1.16173600

C	4.58885000	-1.23549200	-0.35186100
H	5.55241400	-0.86010500	-0.70533100
H	4.75784400	-1.73816100	0.60486100
H	4.23564700	-1.98482300	-1.06504800

TS13

C	0.93658500	-0.37039900	-0.19248400
C	2.03166400	-1.18540200	0.05814000
H	1.97379200	-1.89381400	0.88351800
O	1.48005100	1.19389800	0.82028100
O	1.41029700	2.32873100	-0.01654000
H	2.33733300	2.53400600	-0.19983500
H	0.93536700	0.20401200	-1.11296200
C	-0.43936700	-0.70518200	0.34070000
C	-1.33735600	0.54042300	0.48242900
C	-1.10893100	-1.77063100	-0.56411600
H	-0.33188900	-1.13140900	1.34644500
C	-2.76453200	0.19944700	0.91456400
H	-0.87324800	1.22237300	1.19310000
C	-2.53118600	-2.10831600	-0.09298200
H	-1.14650100	-1.38899500	-1.59055900
H	-0.48898500	-2.67232200	-0.58403500
C	-3.40622400	-0.85176300	0.00127800
H	-3.34758900	1.12434300	0.92044600
H	-2.73730700	-0.16881400	1.94859400
H	-2.97999000	-2.83527900	-0.77743600
H	-2.48200600	-2.59462000	0.89044900
H	-4.40536000	-1.10956900	0.36580700
H	-3.53275700	-0.42147800	-0.99770100
O	-1.33466400	1.18525200	-0.80818500
O	-1.43648100	2.61466200	-0.60800500
H	-0.49249800	2.83169800	-0.50951200
C	3.35230000	-1.06096500	-0.63015600
H	3.59379300	-2.01399200	-1.12163300
H	3.28493200	-0.31178000	-1.42529300
C	4.49785800	-0.70465900	0.33634900
H	4.61510700	-1.47240500	1.10637100
H	5.44728700	-0.61959300	-0.19892400
H	4.29203000	0.24196900	0.84042700

P13

C	-2.21959300	0.64294800	0.99999500
C	-0.74405500	0.57922400	0.58645300
C	-0.27744900	-0.87694500	0.40316100
C	-1.18174800	-1.62110700	-0.60818700
C	-2.66012100	-1.55359700	-0.19830700

C	-3.12533500	-0.10584600	0.01154900
H	-0.11423200	1.07685400	1.33190600
H	-2.31431200	0.20743000	2.00200800
H	-2.51579100	1.69128100	1.08601700
H	-1.04377600	-1.17746800	-1.59915300
H	-0.85768000	-2.66432200	-0.68138500
H	-3.28143300	-2.04365000	-0.95475600
H	-2.80129800	-2.11743500	0.73336200
H	-3.11367500	0.41612500	-0.95190800
H	-4.16066700	-0.08552500	0.36595300
H	-0.46189500	-1.34287100	1.38359500
C	1.18991800	-1.08032100	0.10892200
H	1.50477800	-2.12317700	0.17259100
C	2.10726400	-0.16701500	-0.20549300
H	1.81410500	0.87513100	-0.29938200
O	-0.53235000	1.23742700	-0.67521400
O	-0.69843500	2.66788000	-0.45240100
H	-1.41845300	2.85501300	-1.06926700
C	3.55923800	-0.46895100	-0.45502200
H	3.72726300	-1.54997100	-0.40415100
H	3.81962000	-0.15950700	-1.47563300
C	4.49579200	0.25048700	0.52967900
H	5.54455400	0.04195600	0.30020400
H	4.35362100	1.33443800	0.48745700
H	4.30207500	-0.07002200	1.55695800

R14

C	-1.68887300	0.21286000	0.72950000
C	-0.26420600	-0.06495600	1.22223000
C	0.86023100	0.28543400	0.22567600
C	0.65324100	1.71704500	-0.33337100
C	-0.75922200	1.94389500	-0.89048200
C	-1.83800500	1.62481800	0.15322700
H	-0.11209800	0.53290000	2.12755700
H	-0.16894900	-1.10894000	1.52134200
H	-2.39086200	0.06141600	1.55922500
H	0.85483300	2.41910100	0.48330200
H	1.40433800	1.90077700	-1.10573300
H	-0.86142700	2.98423400	-1.21513000
H	-0.90568900	1.31444200	-1.77012100
H	-1.76866900	2.32525000	0.99383200
H	-2.83932300	1.73649800	-0.27210000
C	2.20866900	0.12284100	0.86204400
H	2.26798200	0.10213900	1.94517800

O	0.76884700	-0.52071000	-0.97825300
O	0.85044400	-1.92752700	-0.63774300
H	-0.09454400	-2.15793200	-0.59063700
O	-2.09742400	-0.64167300	-0.35183600
O	-1.95235000	-2.03033900	0.08980400
H	-2.75671700	-2.40309400	-0.29480900
C	3.42451200	-0.16919700	0.05532100
H	4.33786300	0.07949200	0.60206900
H	3.47328100	-1.23552800	-0.20997200
H	3.42577300	0.37480100	-0.89464700

TS14

C	-1.56536100	0.56773200	0.74749000
C	-0.24161600	-0.01808200	1.25900400
C	0.98562100	0.17762400	0.38155400
C	1.04915700	1.45649700	-0.43523700
C	-0.30667600	1.91353900	-0.98864400
C	-1.38329200	1.94283900	0.10136900
H	-0.04478100	0.46500300	2.22449900
H	-0.36743700	-1.08120800	1.47661000
H	-2.27279300	0.62398800	1.58305900
H	1.46419600	2.24602100	0.20769600
H	1.75576300	1.32058000	-1.25701500
H	-0.20153600	2.90867600	-1.43117700
H	-0.61644000	1.23304500	-1.78299200
H	-1.10386100	2.64575500	0.89538100
H	-2.34230200	2.27940700	-0.30182700
C	2.16392500	-0.43370100	0.80817400
H	2.06359800	-1.26837200	1.49615500
O	0.62665600	-0.97439400	-1.13182600
O	0.14505900	-2.22766500	-0.75620800
H	-0.80535100	-2.07461600	-0.60981700
O	-2.18537900	-0.21644600	-0.28564900
O	-2.47674900	-1.53906800	0.27039700
H	-3.37934800	-1.65596200	-0.05567300
C	3.51981500	-0.19701300	0.22520200
H	3.62510700	-0.73260400	-0.72828100
H	3.71657900	0.85973600	0.02386500
H	4.30247300	-0.56547000	0.89233200

P14

C	-1.35498100	-0.17825300	0.72758800
C	0.02157500	-0.47997200	1.33984800
C	1.17177200	-0.03542700	0.46038300
C	1.06184400	1.40290900	0.00911300
C	-0.29988400	1.66902000	-0.66378000

C	-1.46652400	1.27228200	0.24894900
H	0.06279100	0.05804300	2.29690400
H	0.08955800	-1.54700800	1.56285900
H	-2.14221900	-0.40048100	1.45660600
H	1.15027800	2.05963600	0.88720700
H	1.87272600	1.67636400	-0.66751900
H	-0.38184800	2.72549300	-0.93769800
H	-0.35261100	1.09297400	-1.59348500
H	-1.48566300	1.92178100	1.13296200
H	-2.42607700	1.40421400	-0.25697300
C	2.15552800	-0.88110900	0.13798500
H	2.08088600	-1.89618500	0.52559600
O	-1.48580600	-1.11570600	-0.35281400
O	-2.86889500	-1.06500400	-0.80672100
H	-2.73435400	-0.83763600	-1.73616500
C	3.37457900	-0.62043800	-0.69908100
H	3.42707300	0.40094300	-1.07773900
H	4.28783400	-0.80817200	-0.12262600
H	3.40578900	-1.29835700	-1.55966700

R15

C	-2.00867900	0.24934800	0.81346600
C	-0.55761600	-0.05991400	1.19864900
C	0.49611600	0.25886300	0.11750500
C	0.28073600	1.69256400	-0.43306700
C	-1.16426800	1.95051200	-0.88294500
C	-2.16804400	1.66189100	0.24128800
H	-0.32341700	0.53858800	2.08583600
H	-0.46424100	-1.10437700	1.49672500
H	-2.64887000	0.11847300	1.69505200
H	0.56006500	2.39319100	0.36161500
H	0.97516500	1.85493800	-1.26124000
H	-1.26630900	2.99156700	-1.20534300
H	-1.39173500	1.32092000	-1.74508500
H	-2.01894800	2.36422900	1.06985300
H	-3.19580800	1.79540600	-0.10781800
C	1.88459700	0.06694800	0.65074400
H	2.02698300	0.05126700	1.72760600
O	0.29330100	-0.55053500	-1.07095300
O	0.36668600	-1.95738300	-0.72902800
H	-0.57714800	-2.16451600	-0.60793600
O	-2.51816300	-0.60019800	-0.22822000
O	-2.37320600	-1.98992100	0.21049200
H	-3.21288000	-2.34502500	-0.11003900

C	3.03729000	-0.26234900	-0.23504800
H	2.99096200	-1.33093500	-0.49819900
H	2.93093300	0.25849800	-1.19439900
C	4.40143700	0.05096700	0.39152400
H	4.53727200	-0.49447300	1.33054200
H	5.21718600	-0.23449400	-0.27795300
H	4.50242400	1.11807100	0.60975400

TS15

C	-1.92755300	0.39894600	0.85595300
C	-0.50694400	-0.05029500	1.22663700
C	0.60663600	0.31104700	0.25541700
C	0.45587200	1.61393400	-0.50971600
C	-0.98585600	1.93286700	-0.92524100
C	-1.95627800	1.80555500	0.25390700
H	-0.27779100	0.42031700	2.19117500
H	-0.49558000	-1.12731300	1.40911000
H	-2.55858100	0.34892800	1.75099600
H	0.83821200	2.42364500	0.12852000
H	1.09823800	1.58599300	-1.39292000
H	-1.03082000	2.94720200	-1.33303400
H	-1.28955300	1.24751400	-1.71779100
H	-1.68505800	2.51010600	1.04933100
H	-2.97893800	2.04422600	-0.05063500
C	1.88007500	-0.17385700	0.54919700
H	1.94297400	-1.03220400	1.21387400
O	0.23958900	-0.82744300	-1.26923400
O	-0.06669100	-2.13764200	-0.90524500
H	-1.01119900	-2.09695900	-0.67208000
O	-2.54897500	-0.41702500	-0.15145400
O	-2.64344700	-1.78076400	0.37262400
H	-3.55435700	-1.98701800	0.12286400
C	3.14352500	0.24146000	-0.14147800
H	3.07985600	-0.06440800	-1.19580900
H	3.22994300	1.33549000	-0.15822100
C	4.40257800	-0.36233300	0.48960800
H	4.36426700	-1.45523700	0.47003700
H	5.30177000	-0.05047400	-0.04749400
H	4.51047900	-0.05077200	1.53284500

P15

C	-1.78046500	-0.25361800	0.73400900
C	-0.37521700	-0.44150900	1.32669000
C	0.72075000	0.10415600	0.43472800
C	0.48330900	1.53282600	0.00159100
C	-0.90366800	1.68733600	-0.65443400

C	-2.02136100	1.18523900	0.26768100
H	-0.36633200	0.09279100	2.28658300
H	-0.21433600	-1.50024800	1.54114900
H	-2.53634200	-0.54672300	1.47112200
H	0.52319100	2.18410200	0.88718500
H	1.26071500	1.88296900	-0.67888000
H	-1.07856000	2.73517300	-0.91774200
H	-0.91766300	1.11641700	-1.58865800
H	-2.08600100	1.82465900	1.15686500
H	-2.99447000	1.23753600	-0.22670300
C	1.76317800	-0.65640200	0.08391000
H	1.78038100	-1.67772000	0.46497300
O	-1.84518000	-1.19185900	-0.35160000
O	-3.23364300	-1.25657700	-0.78711700
H	-3.13100900	-1.01620600	-1.71735700
C	2.95109100	-0.29345300	-0.76399400
H	2.85014500	0.71282100	-1.17726900
H	2.99117300	-0.97501600	-1.62365300
C	4.27883600	-0.39842800	0.00633400
H	4.41951500	-1.40412100	0.41356600
H	5.13021800	-0.17981700	-0.64440800
H	4.30124300	0.30397800	0.84388200

R16

C	-2.46084600	0.21729100	0.75928100
C	-1.01904200	-0.05100300	1.20514100
C	0.06909700	0.28636200	0.16450100
C	-0.16081400	1.70919900	-0.40797400
C	-1.59262600	1.92538900	-0.91803700
C	-2.63314600	1.62032700	0.16778900
H	-0.83640400	0.56034100	2.09567900
H	-0.91095500	-1.09030100	1.51595400
H	-3.13244600	0.07730500	1.61574600
H	0.06789100	2.42366500	0.39055900
H	0.56215400	1.88260100	-1.20905100
H	-1.70838200	2.96061100	-1.25430400
H	-1.76878400	1.28265600	-1.78249500
H	-2.53556600	2.33330600	0.99493200
H	-3.64909900	1.72415000	-0.22345100
C	1.43905800	0.13553000	0.75556800
H	1.53729400	0.13537200	1.83740000
O	-0.06428500	-0.53804600	-1.02338500
O	0.03181700	-1.93954300	-0.66547700
H	-0.91045300	-2.17048900	-0.58079000

O	-2.90620400	-0.65379400	-0.29392500
O	-2.74286700	-2.03565000	0.16287700
H	-3.55917600	-2.41540500	-0.18828200
C	2.63590200	-0.16746300	-0.07870600
H	2.63959000	-1.24218900	-0.32500700
H	2.55170500	0.33389000	-1.05163500
C	3.96831800	0.20147000	0.58968100
H	4.03808400	-0.31152700	1.55645400
H	3.97632400	1.27471500	0.81228500
C	5.18599100	-0.15608600	-0.26636100
H	6.11993800	0.11242000	0.23504300
H	5.21749800	-1.22927400	-0.47829100
H	5.16173600	0.36833900	-1.22676800

TS16

C	-2.36802500	0.40387000	0.82761500
C	-0.95840800	-0.05070700	1.23230200
C	0.17944800	0.30667600	0.28820200
C	0.05219300	1.61103000	-0.47850000
C	-1.37762200	1.93511000	-0.92959300
C	-2.37725300	1.81063400	0.22522500
H	-0.75074800	0.41894900	2.20217800
H	-0.95547100	-1.12778600	1.41475800
H	-3.02058800	0.35615500	1.70721500
H	0.42076800	2.41848300	0.17062800
H	0.71692400	1.58261200	-1.34499500
H	-1.40906400	2.94978700	-1.33781300
H	-1.66386500	1.25111000	-1.72977700
H	-2.12339700	2.51419600	1.02723300
H	-3.39134800	2.05265800	-0.10429300
C	1.44380100	-0.18324700	0.61152600
H	1.48787800	-1.04062300	1.27885600
O	-0.15549800	-0.82866400	-1.24563800
O	-0.47518200	-2.13812000	-0.89006300
H	-1.42478600	-2.09399300	-0.67941700
O	-2.96808200	-0.40984300	-0.19447600
O	-3.08056300	-1.77311600	0.32729700
H	-3.98620100	-1.97558700	0.05618900
C	2.72383500	0.22884400	-0.04870300
H	2.67417600	-0.04568500	-1.11325600
H	2.83194600	1.32232900	-0.03294100
C	3.96811800	-0.41169700	0.58005200
H	3.86415000	-1.50201800	0.54210300
H	4.01315300	-0.14447700	1.64247600
C	5.26951900	0.00367700	-0.10942400

H	5.41783400	1.08703600	-0.05733900
H	6.13684000	-0.47222800	0.35587800
H	5.26275900	-0.27810800	-1.16686400

P16

C	-2.30792500	-0.25007400	0.67729400
C	-0.94215200	-0.43120200	1.35775000
C	0.20667900	0.10803500	0.53080800
C	-0.00409600	1.53271100	0.07158900
C	-1.34812900	1.68087400	-0.66976300
C	-2.52046400	1.18468400	0.18530400
H	-0.99335900	0.11128400	2.31170700
H	-0.79420500	-1.48788900	1.59086800
H	-3.10800100	-0.53821300	1.36828300
H	-0.01860200	2.19161100	0.95231000
H	0.81375500	1.87733400	-0.56272900
H	-1.50698900	2.72653100	-0.95130400
H	-1.30420800	1.10303400	-1.59879500
H	-2.64072700	1.83107700	1.06360400
H	-3.46106000	1.23187400	-0.36891600
C	1.26905600	-0.65461400	0.25131600
H	1.26217500	-1.67246700	0.64144100
O	-2.30403300	-1.19716700	-0.40251600
O	-3.66242300	-1.26700400	-0.92360900
H	-3.50204700	-1.03277100	-1.84723000
C	2.50658400	-0.29690800	-0.52369300
H	2.42388300	0.69786000	-0.97026200
H	2.61559400	-1.00006000	-1.36097400
C	3.78715000	-0.36085700	0.33088400
H	3.86251800	-1.35307800	0.79096800
H	3.69784100	0.35197000	1.15803300
C	5.05772300	-0.06974500	-0.47204200
H	5.94895500	-0.12216900	0.15920200
H	5.18451900	-0.78970000	-1.28670700
H	5.02504500	0.92973100	-0.91719100

R17

C	2.29507300	-1.34110600	-0.33012400
C	0.80375700	-1.66011200	-0.54313900
C	-0.10174700	-0.87367700	0.35169500
C	0.21935100	0.55747400	0.64522600
C	1.71315400	0.83738800	0.80812800
C	2.54535000	0.17098200	-0.29293700
H	0.53832300	-1.43472100	-1.59116400
H	0.61697800	-2.73258800	-0.42015600

H	2.62587000	-1.78574000	0.61637500
H	2.88598900	-1.80993200	-1.12231300
H	-0.34594700	0.90507500	1.51676800
H	1.86132600	1.91954200	0.82107500
H	2.02197600	0.45795800	1.78996600
H	2.28230800	0.61343200	-1.25974700
H	3.60774100	0.37489100	-0.12993100
H	-1.94890500	0.42151200	-1.08519900
C	-1.45645900	-1.38785300	0.73473700
H	-1.67774300	-2.33499800	0.23113900
H	-1.53472400	-1.54632700	1.82048300
O	-2.50974600	-0.45057000	0.49052200
O	-2.61663100	-0.27632900	-0.94648100
O	-0.31159900	1.29405500	-0.51411000
O	-0.29293000	2.71810600	-0.22120700
H	-1.19682200	2.85122200	0.10140800

TS17

C	1.92671600	-1.62970500	-0.46801300
C	0.38516900	-1.64014700	-0.50322800
C	-0.21404000	-0.78471700	0.57447900
C	0.37470500	0.59157400	0.74591000
C	1.90478900	0.59323600	0.74798700
C	2.47772300	-0.19899100	-0.43394400
H	0.04747200	-1.25395600	-1.47764100
H	-0.00122800	-2.66028600	-0.43087000
H	2.26898000	-2.17455600	0.42021300
H	2.31453000	-2.16812400	-1.33766300
H	-0.02259500	1.07109100	1.64688600
H	2.25378800	1.62808000	0.73733000
H	2.23683500	0.14905200	1.69433600
H	2.22198500	0.31493000	-1.36629300
H	3.56990700	-0.21575800	-0.37130900
H	-1.90348900	0.55057900	-1.25066000
C	-1.40880000	-1.10482400	1.19714500
H	-1.79455300	-2.11322100	1.10544600
H	-1.70494800	-0.56928100	2.09209800
O	-2.75546600	-0.18650600	0.22542200
O	-2.51582100	-0.19924400	-1.14369800
O	-0.14286200	1.33078900	-0.40475200
O	0.21877600	2.72886400	-0.26727100
H	-0.60128800	3.09167500	0.09850000

P17

C	-0.41759800	-0.93012600	1.08423800
C	-0.58877100	0.47047200	0.47921900

C	0.74126300	1.04423700	0.03498900
C	1.55603100	0.14191600	-0.86045300
C	1.72475100	-1.25795600	-0.23596800
C	0.36933400	-1.86266500	0.15375100
H	-1.07591400	1.14246800	1.19496900
H	0.11031200	-0.81682000	2.03875700
H	-1.40518200	-1.33983600	1.30602800
H	1.03674400	0.03829400	-1.82062300
H	2.52906700	0.59682900	-1.06472500
H	2.24672800	-1.91448600	-0.93909000
H	2.36005500	-1.17764900	0.65474700
H	-0.22208900	-2.04572900	-0.74918100
H	0.51345500	-2.83266000	0.63959700
C	1.15431400	2.23516300	0.46482200
H	2.12408500	2.63342100	0.18460300
H	0.53950000	2.85149100	1.11231300
O	-1.38528600	0.43411500	-0.72492700
O	-2.71103400	-0.05188000	-0.37391300
H	-3.22214800	0.76055400	-0.49026800

R18

C	1.69114500	-2.05015000	-0.39615900
C	0.23053900	-1.63006500	-0.65836200
C	-0.25681800	-0.59443400	0.30754300
C	0.67286100	0.52781300	0.65939900
C	2.12160900	0.08907400	0.87515300
C	2.61135000	-0.83416000	-0.24555600
H	0.16406200	-1.21657900	-1.67962300
H	-0.42081700	-2.50687100	-0.65319300
H	1.73118500	-2.65200500	0.51995900
H	2.03503300	-2.69348700	-1.21150400
H	0.29454100	1.07871300	1.52687700
H	2.74471500	0.98271200	0.95676900
H	2.17583700	-0.43141500	1.83928400
H	2.63828700	-0.27350400	-1.18594700
H	3.63624500	-1.15722700	-0.03982900
H	-1.23240800	1.37855900	-1.16698500
C	-1.69588500	-0.43471800	0.72521100
H	-1.73736400	-0.36676600	1.82317300
O	-2.18037200	0.88882800	0.38309900
O	-2.13927600	1.03571200	-1.06205000
O	0.58907900	1.44537700	-0.48708400
O	1.23178200	2.70094700	-0.13811000
H	0.46928000	3.22014600	0.15829200

C	-2.66741200	-1.50938900	0.25346700
H	-2.37225700	-2.49608600	0.61979600
H	-3.66339300	-1.28259300	0.63736800
H	-2.71699100	-1.53433400	-0.83546900

TS18

C	-1.49867000	-2.04333000	0.62480200
C	-0.07459000	-1.46991800	0.78694100
C	0.30600300	-0.57633600	-0.35838000
C	-0.71806600	0.45253700	-0.76048700
C	-2.13034000	-0.12057100	-0.89592200
C	-2.52645700	-0.94333200	0.33613700
H	-0.03055400	-0.88311400	1.71771000
H	0.64705600	-2.28034200	0.90403700
H	-1.49919900	-2.76825300	-0.19834200
H	-1.77201900	-2.59398700	1.52978000
H	-0.40821400	0.96376300	-1.67765300
H	-2.82816400	0.70296300	-1.06327200
H	-2.15149900	-0.75132200	-1.79306400
H	-2.60282700	-0.27636600	1.20095800
H	-3.51759200	-1.38182700	0.18573100
H	1.14274100	1.51836800	1.33924300
C	1.59104600	-0.49125200	-0.88842600
H	1.67817800	0.03346100	-1.83602100
O	2.28972500	1.09243900	-0.05386500
O	2.02776800	1.11253300	1.31346800
O	-0.67041200	1.43693900	0.32146700
O	-1.46355800	2.58967100	-0.05877300
H	-0.77370600	3.17684700	-0.40176300
C	2.67827800	-1.48876500	-0.59690700
H	3.62619100	-1.13686100	-1.00377200
H	2.80571900	-1.62747000	0.47778500
H	2.45247800	-2.46270700	-1.04628400

P18

C	1.29964900	0.51740100	1.09924500
C	0.53778000	-0.65553200	0.46803400
C	-0.84385800	-0.24572200	-0.00223600
C	-0.85933400	0.98172100	-0.88489000
C	-0.10347900	2.15571000	-0.22977000
C	1.31587600	1.74794400	0.18405100
H	0.46722000	-1.48908000	1.17643800
H	0.80507200	0.76021500	2.04758300
H	2.31459200	0.19177600	1.33640500
H	-0.36575400	0.73311200	-1.83214500
H	-1.88022400	1.28035700	-1.12868400

H	-0.06943300	3.00220400	-0.92305300
H	-0.66050900	2.49180400	0.65369900
H	1.90489800	1.51871000	-0.70995000
H	1.81768000	2.57914400	0.68933400
C	-1.91100600	-0.94465400	0.40011000
H	-1.72618400	-1.80982400	1.03545000
O	1.19765300	-1.13398300	-0.72676600
O	2.52380000	-1.60663100	-0.35549500
H	2.39802900	-2.55870500	-0.46672300
C	-3.35765800	-0.69591400	0.08834700
H	-3.51922800	0.19186300	-0.52328500
H	-3.78749800	-1.55163300	-0.44484400
H	-3.93811000	-0.58075400	1.01052600

R19

C	-1.67908100	-2.25867700	0.40586900
C	-0.33888100	-1.57790900	0.75161500
C	0.03317200	-0.50372800	-0.22409200
C	-1.05022000	0.42735400	-0.68050100
C	-2.37899300	-0.26702100	-0.98040800
C	-2.78427600	-1.23117200	0.13941500
H	-0.42575500	-1.12556000	1.75460500
H	0.45293600	-2.32540300	0.83337700
H	-1.54271800	-2.88518400	-0.48411300
H	-1.96606800	-2.92839100	1.22202000
H	-0.71075800	1.01640900	-1.53884900
H	-3.14082800	0.49918200	-1.14204600
H	-2.26680000	-0.81501300	-1.92405900
H	-2.98250900	-0.65821700	1.05143400
H	-3.71721700	-1.73766100	-0.12559200
H	0.53346100	1.63695000	1.24873200
C	1.44802700	-0.10073800	-0.55134300
H	1.55473200	-0.04413300	-1.64647800
O	1.66298000	1.29553000	-0.21725900
O	1.49190600	1.45919000	1.21688500
O	-1.21435500	1.37082500	0.43568900
O	-2.04021100	2.48396700	-0.00025600
H	-1.36107200	3.12351600	-0.26212200
C	2.56048500	-0.98806900	0.00838100
H	2.36943900	-2.02072400	-0.30333800
H	2.50802000	-0.96543600	1.09974300
C	3.95177700	-0.55307400	-0.45769400
H	4.15179200	0.47835100	-0.16372300
H	4.72443200	-1.19046900	-0.02066400

H	4.04429500	-0.61610500	-1.54684100

TS19

C	-1.57217400	-2.18880400	0.63820500
C	-0.28323500	-1.38859500	0.92405200
C	0.08770600	-0.49234200	-0.22243100
C	-1.02814800	0.34414900	-0.79127500
C	-2.30872700	-0.45201900	-1.05088200
C	-2.71630400	-1.28042200	0.17378800
H	-0.44169700	-0.76546900	1.81800200
H	0.53621300	-2.06685300	1.16839900
H	-1.36244300	-2.93647200	-0.13645200
H	-1.86273100	-2.73994600	1.53751800
H	-0.69476000	0.86578600	-1.69403700
H	-3.10071200	0.24245700	-1.34029300
H	-2.12579700	-1.11121300	-1.90828100
H	-2.99857000	-0.60191700	0.98529200
H	-3.60194100	-1.87833800	-0.06158100
H	0.38819200	1.75949900	1.44051000
C	1.39841200	-0.22931900	-0.61298200
H	1.51981600	0.26084500	-1.57647600
O	1.72777400	1.49242700	0.18748800
O	1.32828100	1.51751700	1.52165900
O	-1.26147800	1.36040400	0.23570700
O	-2.16569500	2.36236400	-0.29371200
H	-1.53462100	3.03633700	-0.58678600
C	2.58459700	-1.03368700	-0.13900300
H	2.61967400	-0.99167800	0.95361000
H	2.42909200	-2.08750300	-0.40549700
C	3.91294900	-0.54376700	-0.71988800
H	4.74791500	-1.13713100	-0.33919600
H	3.92083600	-0.62116200	-1.81181100
H	4.08001800	0.49990200	-0.44956900

P19

C	1.88151900	0.28923100	1.03018200
C	0.80046900	-0.66963900	0.51341000
C	-0.45843100	0.06780400	0.10078700
C	-0.21646200	1.21464800	-0.85455700
C	0.85923500	2.17879900	-0.31376200
C	2.15066800	1.43313700	0.04466700
H	0.56754300	-1.42566500	1.27213400
H	1.53408900	0.69241000	1.98914000
H	2.79218700	-0.27992500	1.22818600
H	0.13283600	0.80178200	-1.80854700
H	-1.13977200	1.75764700	-1.06271000

H	1.06243900	2.95577800	-1.05763800
H	0.46950100	2.68685600	0.57713500
H	2.59801600	1.02068700	-0.86555500
H	2.88299000	2.12551200	0.47166700
C	-1.63791800	-0.30338200	0.61193900
H	-1.63954900	-1.16047400	1.28579900
O	1.22819900	-1.35257300	-0.68738200
O	2.40983600	-2.13992300	-0.36614000
H	2.03447500	-3.02967000	-0.41226400
C	-2.99535100	0.29473800	0.36830400
H	-2.92413900	1.20243000	-0.23472500
H	-3.42463600	0.59726200	1.33212400
C	-3.95767600	-0.69721800	-0.30856200
H	-4.06751900	-1.60891300	0.28616500
H	-4.95102000	-0.25711900	-0.43179500
H	-3.58804100	-0.98734100	-1.29539000

R20

C	1.73598200	2.40869500	0.39925600
C	0.49857600	1.55173100	0.73608200
C	0.28855700	0.43213100	-0.23687000
C	1.49473100	-0.34417900	-0.67431300
C	2.71958600	0.52378000	-0.96434300
C	2.97512700	1.54170100	0.15229600
H	0.63637800	1.12031400	1.74258600
H	-0.38942800	2.18331200	0.80551400
H	1.52464100	3.00497400	-0.49679500
H	1.91869100	3.11648200	1.21328600
H	1.24957200	-0.97898100	-1.53217800
H	3.58110500	-0.13190700	-1.11085900
H	2.54548600	1.04500800	-1.91376100
H	3.23970900	1.00714500	1.07078200
H	3.83250600	2.17017000	-0.10667600
H	0.06887100	-1.74739900	1.24654100
C	-1.05387200	-0.16272300	-0.57721900
H	-1.13712400	-0.24078200	-1.67294600
O	-1.07905300	-1.57363600	-0.23418200
O	-0.90444700	-1.70334300	1.20281200
O	1.77299500	-1.25015800	0.45028000
O	2.74782000	-2.24268300	0.03111400
H	2.16522500	-2.97001100	-0.23423500
C	-2.28323700	0.56757600	-0.03721500
H	-2.22763100	1.61875800	-0.34402000
H	-2.24906000	0.54896400	1.05596200

C	-3.60378300	-0.03835700	-0.52467000
H	-3.62688100	-1.09718100	-0.25476100
H	-3.63827500	0.00182800	-1.62070200
C	-4.82990900	0.67476400	0.05133700
H	-4.84474600	1.73381400	-0.22626700
H	-5.75744100	0.22420900	-0.31189800
H	-4.84066400	0.61913800	1.14419300

TS20

C	1.73598200	2.40869500	0.39925600
C	0.49857600	1.55173100	0.73608200
C	0.28855700	0.43213100	-0.23687000
C	1.49473100	-0.34417900	-0.67431300
C	2.71958600	0.52378000	-0.96434300
C	2.97512700	1.54170100	0.15229600
H	0.63637800	1.12031400	1.74258600
H	-0.38942800	2.18331200	0.80551400
H	1.52464100	3.00497400	-0.49679500
H	1.91869100	3.11648200	1.21328600
H	1.24957200	-0.97898100	-1.53217800
H	3.58110500	-0.13190700	-1.11085900
H	2.54548600	1.04500800	-1.91376100
H	3.23970900	1.00714500	1.07078200
H	3.83250600	2.17017000	-0.10667600
H	0.06887100	-1.74739900	1.24654100
C	-1.05387200	-0.16272300	-0.57721900
H	-1.13712400	-0.24078200	-1.67294600
O	-1.07905300	-1.57363600	-0.23418200
O	-0.90444700	-1.70334300	1.20281200
O	1.77299500	-1.25015800	0.45028000
O	2.74782000	-2.24268300	0.03111400
H	2.16522500	-2.97001100	-0.23423500
C	-2.28323700	0.56757600	-0.03721500
H	-2.22763100	1.61875800	-0.34402000
H	-2.24906000	0.54896400	1.05596200
C	-3.60378300	-0.03835700	-0.52467000
H	-3.62688100	-1.09718100	-0.25476100
H	-3.63827500	0.00182800	-1.62070200
C	-4.82990900	0.67476400	0.05133700
H	-4.84474600	1.73381400	-0.22626700
H	-5.75744100	0.22420900	-0.31189800
H	-4.84066400	0.61913800	1.14419300

TS20

C	1.67131600	2.31276200	0.64969500
C	0.50101300	1.34898900	0.94120900

C	0.23654000	0.42143500	-0.20974200
C	1.44501900	-0.25778500	-0.79847400
C	2.60742100	0.70101400	-1.06444400
C	2.91832100	1.56518300	0.16377700
H	0.74977900	0.74451300	1.82737700
H	-0.39712500	1.91221000	1.20076200
H	1.35680400	3.03359000	-0.11489800
H	1.89848800	2.88916600	1.55141600
H	1.17182700	-0.81141700	-1.70240200
H	3.47968700	0.11839600	-1.36944200
H	2.32948900	1.33784000	-1.91317800
H	3.29637200	0.92251800	0.96541600
H	3.71560500	2.27543200	-0.07530200
H	0.25177900	-1.86233400	1.43582100
C	-1.03348100	-0.00704000	-0.58872300
H	-1.10086900	-0.50097300	-1.55553600
O	-1.12484800	-1.76300500	0.19822700
O	-0.71096800	-1.74634700	1.52837000
O	1.82135900	-1.24276800	0.21644000
O	2.84193400	-2.11417000	-0.33215400
H	2.30056900	-2.86258900	-0.62399300
C	-2.30759500	0.63245100	-0.09463300
H	-2.33356300	0.56307400	0.99788500
H	-2.29006500	1.70405600	-0.33792700
C	-3.57362300	-0.00257700	-0.68031300
H	-3.55058400	0.08875600	-1.77337900
H	-3.55865800	-1.07162800	-0.45332600
C	-4.85883500	0.63032800	-0.14212000
H	-5.74445800	0.15993800	-0.57733400
H	-4.92647000	0.52111900	0.94463600
H	-4.90499200	1.70008100	-0.37106100
P20			
C	2.39534100	0.28630700	0.98257600
C	1.29107700	-0.67026800	0.51317300
C	0.01861700	0.06998900	0.15041300
C	0.22450800	1.21405600	-0.81660100
C	1.32501600	2.17577600	-0.32350500
C	2.62764900	1.42668800	-0.01636700
H	1.08703600	-1.42348600	1.28293600
H	2.08858900	0.69330300	1.95372100
H	3.31154300	-0.28525200	1.14489100
H	0.53204300	0.79807300	-1.78354500
H	-0.70506600	1.75955900	-0.98701400
H	1.49998000	2.94997200	-1.07740600

H	0.97413100	2.68774300	0.58119900
H	3.03593800	1.01029000	-0.94300400
H	3.37912900	2.11772000	0.37829700
C	-1.14010900	-0.29657800	0.71033300
H	-1.11557600	-1.15179100	1.38588700
O	1.66707000	-1.35790800	-0.70224100
O	2.85877100	-2.14783300	-0.42770000
H	2.47908100	-3.03653500	-0.45439600
C	-2.50402300	0.30426500	0.51947500
H	-2.45444400	1.22301000	-0.07072200
H	-2.90608100	0.58955200	1.50123600
C	-3.49440200	-0.67210600	-0.14501000
H	-3.53295800	-1.59866600	0.43967000
H	-3.10917600	-0.95036800	-1.13177400
C	-4.90426400	-0.09120100	-0.28035900
H	-5.58392800	-0.80400600	-0.75497100
H	-5.32232700	0.16626300	0.69790500
H	-4.90111000	0.81898400	-0.88816100

R21

C	-1.40335500	0.23951400	0.76262200
C	0.00630100	-0.08862200	1.14087800
C	1.14393800	0.23805300	0.22164900
C	0.96691800	1.65149300	-0.37793700
C	-0.45417200	1.91636700	-0.89269100
C	-1.51257800	1.65923200	0.18807800
H	-2.06493100	0.11421800	1.62629900
H	1.22306800	2.36810800	0.41036100
H	1.70049700	1.78181700	-1.17976100
H	-0.52482800	2.95166700	-1.24037400
H	-0.65431700	1.27244300	-1.75114700
H	-1.39294600	2.36878200	1.01464200
H	-2.52008600	1.79598800	-0.21433200
C	2.50339700	0.06075500	0.89384500
H	2.64200600	-0.98062500	1.18973800
H	3.30537100	0.33954300	0.20599700
H	2.57615000	0.68623100	1.78720700
O	1.09116400	-0.59355500	-0.98560200
O	1.02062900	-1.99795500	-0.57033800
H	0.20162100	-0.67369400	2.03126800
H	1.55365400	-2.39433500	-1.27203500
O	-1.89711100	-0.60543100	-0.30477000
O	-1.79431700	-1.98818000	0.11168800
H	-0.91087600	-2.20993100	-0.23434700

TS21

C	-1.10062700	0.47476100	0.89541900
C	0.29814200	0.12078500	1.28841400
C	1.38002600	0.22995200	0.40737300
C	1.24687800	1.10557500	-0.82434700
C	-0.19503900	1.33053600	-1.30567000
C	-1.15804200	1.59201200	-0.14469000
H	-1.68969100	0.74302100	1.78020600
H	1.70800600	2.07055100	-0.57021300
H	1.85255100	0.67479500	-1.62541200
H	-0.21716900	2.17211400	-2.00431100
H	-0.52737200	0.45156300	-1.85727800
H	-0.90074500	2.52864800	0.36519900
H	-2.18520400	1.69207800	-0.50169100
C	2.77657600	0.09790300	0.96605500
H	2.82513200	-0.70358300	1.70563600
H	3.48793100	-0.12410700	0.16942800
H	3.07703900	1.03256100	1.45121000
O	1.32155800	-1.54937100	-0.25114300
O	0.47310300	-1.70233500	-1.34633500
H	0.44259600	-0.38928000	2.23449700
H	-0.38767200	-1.87423400	-0.92561700
O	-1.65775400	-0.77842000	0.38805600
O	-3.07567100	-0.58420100	0.11287000
H	-3.46277200	-1.06865400	0.85544800

P21

C	-0.86832600	-0.38014500	0.57630200
C	0.46815700	-1.03092100	0.35095700
C	1.57714700	-0.36103400	0.01906600
C	1.57242000	1.13711200	-0.19089500
C	0.16325200	1.71241900	-0.36836200
C	-0.78413700	1.14231300	0.68886200
H	-1.34354800	-0.80118700	1.46960000
H	2.07034900	1.61230000	0.66713700
H	2.19521100	1.37892800	-1.06015500
H	0.19038300	2.80481600	-0.32006700
H	-0.21239800	1.44253200	-1.36101000
H	-0.42491600	1.39913800	1.69278600
H	-1.79152300	1.55568100	0.59790200
C	2.90362000	-1.05096600	-0.14912800
H	2.83451400	-2.12024000	0.05872600
H	3.28250300	-0.92186400	-1.16935100
H	3.65572400	-0.61884400	0.52140900
H	0.49890300	-2.11010600	0.46702500

O	-1.66539200	-0.79548700	-0.55550300
O	-3.06400000	-0.53765500	-0.21565500
H	-3.31287600	0.03131700	-0.95554700

R22

C	2.42034900	1.03638200	0.35109600
C	0.94185200	1.39842900	0.53028800
C	1.69585900	-1.06876600	-0.82162300
C	2.61460800	-0.48129300	0.19459500
H	0.57783300	0.98943500	1.47569500
H	0.83123500	2.48650600	0.59285900
H	2.81103700	1.54355800	-0.53952400
H	3.00256100	1.40112200	1.20226700
H	-0.96560800	-0.65606400	1.46948300
H	1.95297000	-2.01132300	-1.29309400
H	2.41521700	-0.96413800	1.16655300
H	3.65553200	-0.71401900	-0.05228600
C	-1.38668500	1.31995100	-0.53608100
H	-1.42840200	2.39303900	-0.32103100
H	-1.93572800	1.13374500	-1.46090000
O	-0.60502200	-1.50865300	-0.16810700
O	-0.34140000	-1.37550700	1.25297000
C	0.08217300	0.88410100	-0.64324000
H	0.45721400	1.39036700	-1.54526400
C	0.28200100	-0.62144100	-0.94068600
H	-0.10339800	-0.84117500	-1.94388400
O	-2.11368500	0.68042600	0.53066700
O	-3.03357100	-0.28644400	-0.03578500
H	-2.42197200	-1.03381200	-0.19493800

TS22

C	2.43296500	-1.07173600	-0.45281300
C	0.95225000	-1.44947900	-0.58681300
C	1.61808200	1.04648900	0.67952900
C	2.62483600	0.44008700	-0.24805100
H	0.51468800	-0.98979800	-1.47762100
H	0.85170900	-2.53236900	-0.70878800
H	2.86031500	-1.60701800	0.40336500
H	2.99206000	-1.39798700	-1.33427800
H	-0.32510200	3.24024000	-0.56728700
H	1.80881700	2.04862000	1.05190600
H	2.54144200	0.95613000	-1.21771400
H	3.63857600	0.65761000	0.10688400
C	-1.28672100	-1.52939500	0.67628900
H	-1.23247900	-2.62345600	0.72033700

H	-1.82423100	-1.17566300	1.56134900
O	-0.56563200	1.38397500	-0.55533400
O	-0.93529800	2.66153400	-0.08923500
C	0.15912200	-0.99694200	0.65009900
H	0.61804500	-1.48164800	1.52920600
C	0.36863500	0.48264500	0.91007700
H	-0.26663600	0.94157400	1.66026700
O	-2.07216300	-1.26490400	-0.47507800
O	-2.83121900	-0.05351900	-0.25562700
H	-2.15772500	0.61706300	-0.49534400

P22

C	2.15526200	-1.07916700	-0.11008000
C	0.63499100	-1.26604300	-0.05222600
C	-0.01837000	-0.21200600	0.86040500
C	0.54688900	1.16813900	0.60325500
C	1.66829900	1.39542000	-0.07909200
C	2.52188500	0.30268600	-0.67009500
H	0.38425600	-2.26971000	0.30638700
H	2.61265000	-1.86631900	-0.71687700
H	1.99927300	2.41962100	-0.23139200
H	3.57993000	0.51908300	-0.48257600
C	-1.54787000	-0.23109500	0.77611900
H	-0.01204200	2.00765300	1.01050800
H	-1.98467100	0.48777900	1.48193500
H	-1.93455600	-1.22972800	1.00919800
H	0.19939000	-0.47681600	1.90724200
H	0.21397000	-1.17197300	-1.05760500
H	2.57055300	-1.17899900	0.90017000
H	2.40943100	0.30920600	-1.76333400
O	-1.91519100	0.12136400	-0.55448500
O	-3.36143200	0.01015600	-0.63452600
H	-3.59171100	0.93043800	-0.82129100

R23

C	-0.91210400	1.47885100	-0.23422100
C	0.41490400	1.71440500	-0.96738900
C	0.29251800	-0.11506900	1.26567700
C	-0.97492000	0.12673100	0.50449500
H	0.51713500	1.00132500	-1.78937200
H	0.41336300	2.71483700	-1.41046800
H	-1.06445700	2.26754200	0.51127600
H	-1.75829100	1.53479400	-0.92431800
H	0.79317300	-2.19633400	-0.30086400
H	0.24192900	-0.62168200	2.22170300

O	1.94152700	-0.71933800	-0.44209200
O	1.71694600	-2.08462300	-0.01853700
C	1.62170100	1.56100400	-0.03123900
H	1.60151300	2.32896300	0.74990800
C	1.62120500	0.18276700	0.64684900
H	2.41273200	0.10828500	1.39997600
H	2.55971900	1.68059700	-0.58060700
C	-2.21608800	0.00542700	1.38496400
H	-3.11917800	0.10988200	0.78237700
H	-2.23601200	-0.97243800	1.87111300
H	-2.21577500	0.78070300	2.15526000
O	-0.96171100	-0.99003600	-0.45985500
O	-2.13523100	-0.92858400	-1.32208100
H	-1.72139800	-0.68052800	-2.16028100

TS23

C	-1.66547600	1.19972600	-0.22834200
C	-0.46245600	1.91820800	-0.85451200
C	-0.05413100	-0.18683500	1.09314600
C	-1.29560000	-0.21986700	0.23769100
H	-0.16090200	1.37631200	-1.75294500
H	-0.75017700	2.92612100	-1.16709600
H	-2.04607800	1.75628300	0.63564700
H	-2.48342400	1.12461500	-0.95073700
H	3.05523100	-1.66560900	0.32923200
H	0.09082300	-0.98952300	1.80761200
O	1.99110200	-0.30342700	-0.41263700
O	3.18075300	-0.71294200	0.20862700
C	0.72498400	1.99948400	0.11355500
H	0.53187300	2.76569700	0.87816700
C	0.98031000	0.71266900	0.85529500
H	1.80082600	0.73248900	1.56438600
C	-2.44158400	-0.94390700	0.93731500
H	-3.33293600	-0.93206500	0.30656500
H	-2.16185000	-1.98372900	1.11940100
H	-2.68087600	-0.47229200	1.89503900
H	1.63693800	2.30305900	-0.40536500
O	-1.01170200	-0.88638700	-1.03276400
O	-0.26680000	-2.10664100	-0.80486200
H	0.63744100	-1.74306100	-0.85169700

P23

C	-1.68599100	-0.76742600	-0.82689400
C	-0.51567500	-1.35997400	-0.03593900
C	0.55997800	-0.31020200	0.28601000
C	-0.06177200	1.00028600	0.72426300

C	-1.35713700	1.28781400	0.59077100
C	-2.36208300	0.36370600	-0.04289900
H	-0.04281700	-2.17622300	-0.58916700
H	-2.41234100	-1.54820700	-1.07017500
H	-1.73023400	2.23671800	0.96939400
H	-3.02892400	0.93786200	-0.69587200
C	1.56388700	-0.81964800	1.32069500
H	0.60585400	1.71575500	1.19440200
H	2.38902800	-0.11268400	1.41995900
H	1.08635000	-0.93914300	2.29689600
H	1.97014000	-1.78379500	1.00549200
H	-0.87385600	-1.77669900	0.91202400
H	-1.30562400	-0.37567600	-1.77459900
H	-3.00888700	-0.05007600	0.74445100
O	1.23646200	-0.15760000	-0.99942400
O	2.23249700	0.89183700	-0.90163500
H	1.75239300	1.63092900	-1.30037600

R24

C	1.79453200	1.05261500	-0.02729700
C	1.18217100	-0.33232000	0.23692100
C	-0.56157600	1.99550700	0.00529600
C	0.77846300	2.02887600	-0.64751700
H	2.16470600	1.44386700	0.92597900
H	2.65721600	0.94537900	-0.69009500
H	-3.66377600	-0.45589800	0.12756600
H	-1.25185600	2.81412600	-0.16917800
H	0.65691600	1.77230600	-1.71141000
H	1.17738900	3.04948800	-0.63340800
O	-1.93526100	0.12106100	-0.38132600
O	-2.79915300	-0.85664800	0.29131200
C	-0.03450600	-0.18930700	1.16933800
H	0.32239500	0.18474100	2.13479100
C	-1.10532800	0.76883400	0.64708900
H	-1.79845300	1.03121100	1.45394700
C	2.19914200	-1.32166200	0.80255900
H	2.53318900	-1.02145000	1.79990700
H	1.75015100	-2.31420100	0.86889400
H	3.06938900	-1.38119800	0.14491500
H	-0.48819000	-1.16367800	1.35552000
O	0.78797300	-0.74496000	-1.09233700
O	-0.02116400	-1.94226600	-1.02732100
H	-0.90563000	-1.53743100	-1.04838100

TS24

C	1.76020900	1.13624000	-0.18689400
C	1.27174100	-0.26709900	0.20888600
C	-0.63704400	1.92213900	0.03931000
C	0.62745600	2.01101300	-0.75173000
H	2.19169700	1.60654800	0.70278900
H	2.56178700	1.04248700	-0.92410000
H	-3.84154100	-0.12703300	-0.14044900
H	-1.38919600	2.69081200	-0.10780600
H	0.40840300	1.69539800	-1.78174700
H	0.95156800	3.05533400	-0.82291000
O	-1.99407600	-0.24990200	-0.41456400
O	-3.15458700	-0.68211300	0.25567400
C	0.13049700	-0.14914900	1.23560500
H	0.55671600	0.19688300	2.18585900
C	-0.94963700	0.81796400	0.82317500
H	-1.79947900	0.89237800	1.49200900
H	-0.29965100	-1.13204100	1.43024300
C	2.40078100	-1.15273300	0.73195300
H	2.02668100	-2.16306200	0.90593600
H	3.20349400	-1.20740100	-0.00679800
H	2.81123700	-0.76387700	1.66828400
O	0.78813100	-0.78490700	-1.05028700
O	0.08773200	-2.03460600	-0.84489800
H	-0.82334600	-1.68444600	-0.77054300

P24

C	0.51615900	-1.41723900	-0.12308800
C	-0.56505200	-0.38786400	0.24540500
C	0.02299300	0.69690600	1.16368300
C	1.40926000	1.14017600	0.77160900
C	2.17508600	0.48659600	-0.10041300
C	1.74091000	-0.77720700	-0.79275300
H	0.07940500	-2.18128300	-0.77183600
H	3.15968200	0.87532900	-0.34680500
H	2.56783900	-1.49626300	-0.80651800
H	1.78377500	2.04571400	1.24213300
H	0.03244100	0.32068400	2.19486100
H	0.81846600	-1.91285300	0.80539400
H	1.51801200	-0.55542900	-1.84305900
H	-0.66342800	1.54889500	1.17272600
C	-1.79749600	-1.04851100	0.86034400
H	-2.56524200	-0.30179100	1.06995200
H	-2.21308800	-1.78309200	0.16683000
H	-1.54024800	-1.55469600	1.79462000
O	-0.90588900	0.16235200	-1.05318700

O	-1.91674400	1.19629000	-0.90233800
H	-1.40770900	1.96850500	-1.18282000

R25

C	-1.82058900	-0.25780300	0.31003200
C	-0.68478500	0.70308500	0.70597600
C	-0.04301700	-2.02603500	-0.10588000
C	-1.29074700	-1.38306700	-0.61097500
H	-2.14743800	-0.72346300	1.24860900
H	3.53375800	-0.08025400	-0.80080600
H	0.17663400	-3.05764900	-0.35560400
H	-1.09183800	-0.94438800	-1.60198600
H	-2.07146100	-2.13557500	-0.76151700
O	1.74744600	-0.69416200	-0.68883000
O	2.81223300	0.22701200	-0.23627900
C	0.48691700	-0.04756300	1.34851900
H	0.13900700	-0.48146200	2.29240000
C	1.03994300	-1.18606700	0.48386800
H	1.74489700	-1.79053800	1.06624500
H	1.28643600	0.64894600	1.60020300
H	-1.06359700	1.44995600	1.41629000
C	-3.01923700	0.46731600	-0.30990800
H	-3.82823800	-0.23684300	-0.52536000
H	-3.40994800	1.22863700	0.37120000
H	-2.73721200	0.96460500	-1.23946900
O	-0.31632500	1.37424200	-0.50421600
O	0.74761200	2.31294400	-0.22000400
H	1.53036300	1.77853800	-0.44537100

TS25

C	-1.88931300	-0.42777900	0.26727700
C	-0.90985900	0.67987300	0.69637500
C	0.18985300	-1.83722700	-0.16016300
C	-1.16955200	-1.44484800	-0.64474600
H	-2.17523500	-0.94673700	1.19096800
H	3.72319300	-0.72229200	-0.88234500
H	0.64157400	-2.73192600	-0.57730600
H	-1.06468800	-1.01117200	-1.65053500
H	-1.79176900	-2.33849400	-0.77150500
O	2.00783600	-0.02842500	-0.60321500
O	3.35938000	-0.11727400	-0.22014400
C	0.28977500	0.08763700	1.44339000
H	-0.06574900	-0.30710500	2.40414300
C	0.95139000	-1.02735000	0.67475300
H	1.84900900	-1.44723100	1.11385400

H	1.01272100	0.86869100	1.67760300
C	-3.15852300	0.12574500	-0.38811400
H	-3.69026300	0.79506600	0.29396200
H	-2.91557300	0.69028400	-1.29014600
H	-3.84060700	-0.68495200	-0.66081900
H	-1.41511400	1.41151400	1.33994400
O	-0.53444700	1.31554900	-0.52974400
O	0.49591100	2.29829400	-0.27929100
H	1.28043700	1.74289500	-0.46129300

P25

C	0.56003500	0.21793000	0.61484000
C	-0.83062900	0.85572700	0.45604700
C	-1.62524400	0.14226600	-0.65393400
C	-1.52108200	-1.35804300	-0.57190500
C	-0.59416700	-1.98867700	0.14762000
C	0.46231400	-1.27328600	0.95003700
H	1.12890700	0.74377400	1.39005600
H	-0.57871500	-3.07517200	0.17069000
H	1.44450300	-1.72982900	0.79314200
H	-2.24266100	-1.93702300	-1.14234600
H	-1.27173300	0.47979700	-1.63694800
H	-1.34343800	0.65799700	1.40611400
H	0.25951200	-1.38406200	2.02400500
H	-2.67723600	0.44489100	-0.59483700
C	-0.76623600	2.37135000	0.24527800
H	-1.77215200	2.79499900	0.17276500
H	-0.25559800	2.86338700	1.07824100
H	-0.22423900	2.61488700	-0.67046000
O	1.21910200	0.44485700	-0.64055700
O	2.62126000	0.09521400	-0.48053100
H	2.70001200	-0.59781500	-1.14961700

R26

C	1.76539000	-0.27313900	0.29177000
C	1.18396100	-1.35586600	-0.63672400
C	-0.52347200	-0.05384600	1.29304300
C	0.65311200	0.69672400	0.76189800
H	0.84157100	-0.88892100	-1.56343500
H	2.10889300	-0.76439100	1.21192400
H	-2.27116100	1.04760600	-0.39512800
H	-1.00476100	0.27464900	2.20496100
H	1.04416000	1.39205200	1.51323800
O	-1.73688900	-0.73539600	-0.71335600
O	-2.77333900	0.21313000	-0.36244200

C	0.02012900	-2.12401900	0.00663700
H	0.37826300	-2.70271800	0.86538600
C	-1.09315000	-1.18700300	0.50145100
H	-1.83187300	-1.73621100	1.09448200
H	-0.41308500	-2.83397900	-0.70366500
H	1.97968200	-2.05860800	-0.90474000
C	2.96035500	0.45748700	-0.33204400
H	3.35439500	1.22244300	0.34366500
H	3.76761700	-0.24881500	-0.54494200
H	2.68141600	0.94664000	-1.26718900
O	0.32460800	1.46658400	-0.42229600
O	-0.79458800	2.34277000	-0.06769800
H	-0.59140900	3.09152300	-0.64439000

TS26

C	1.85027900	-0.45707500	0.28238600
C	1.09510500	-1.36783100	-0.70352100
C	-0.37454000	0.12878600	1.29647300
C	0.91194200	0.67573900	0.74519600
H	0.84121300	-0.78462800	-1.59178300
H	2.08602300	-1.03742600	1.18475600
H	-3.59377400	0.74336200	-0.00227300
H	-0.90156400	0.70372900	2.04840800
H	1.40191200	1.31350200	1.49039100
O	-2.01866800	-0.07373600	-0.62628800
O	-3.37145400	-0.16185000	-0.26508200
C	-0.18405700	-1.96030800	-0.09440300
H	0.07735800	-2.77744400	0.59305700
C	-0.98992100	-0.97343400	0.71121700
H	-1.87584300	-1.37249800	1.19309500
H	-0.82043900	-2.39947100	-0.86619800
C	3.16280700	0.07634000	-0.30290700
H	3.82880700	-0.75074500	-0.56463500
H	2.98009400	0.66838300	-1.20133700
H	3.68547400	0.71277000	0.41712800
H	1.75117800	-2.18061200	-1.02961800
O	0.68275100	1.46062500	-0.45141900
O	-0.43461700	2.35680800	-0.24780800
H	-1.15422600	1.77300600	-0.55286300

P26

C	-1.44703700	0.67325000	0.66270600
C	-0.44431900	1.04607900	-0.44114400
C	0.61495100	-0.06210300	-0.58305900
C	0.00194800	-1.44167400	-0.60780400
C	-1.24338500	-1.69057600	-0.20127600

C	-2.17506200	-0.63688300	0.33426900
H	-2.17134100	1.48206600	0.79937500
H	-1.63200400	-2.70437700	-0.26550000
H	-2.69208500	-1.02098200	1.22099100
H	0.62511900	-2.24693000	-0.98306500
H	-0.98397500	1.04779300	-1.39784500
H	-0.90505500	0.56539300	1.60787600
H	-2.96587500	-0.45508900	-0.40798900
H	1.20206700	0.10247400	-1.49448500
C	0.17326500	2.43398800	-0.24053000
H	0.89200300	2.66466800	-1.03210100
H	-0.60537400	3.20199100	-0.25844500
H	0.70019300	2.49923100	0.71279400
O	1.51558400	0.10001400	0.53556700
O	2.63745200	-0.79759700	0.33667900
H	2.42988100	-1.48806400	0.98144900

R27

C	-1.70626800	0.33803400	0.76650000
C	-0.32286900	-0.09563700	1.13377800
C	0.83695100	0.14564000	0.21448700
C	0.75955600	1.56858200	-0.38581800
C	-0.64274200	1.93763400	-0.88971200
C	-1.71179400	1.76286300	0.19588700
H	-2.36941700	0.25966100	1.63448100
H	1.07417600	2.26513800	0.39981600
H	1.48645800	1.65232500	-1.19789800
H	-0.63668600	2.97459000	-1.23974000
H	-0.89556700	1.30919000	-1.74580700
H	-1.53330900	2.45967200	1.02269000
H	-2.70819500	1.97694800	-0.20050100
C	2.17063100	-0.15102700	0.92206000
H	2.12985700	-1.18993900	1.26211100
H	2.21527400	0.46918600	1.82410800
O	0.72058700	-0.68014100	-0.99258900
O	0.53123600	-2.07431100	-0.57643700
H	-0.16798200	-0.69545700	2.02254900
H	0.99348600	-2.51515600	-1.30152300
O	-2.26914900	-0.46514600	-0.29916000
O	-2.27085700	-1.85202200	0.11619100
H	-1.40422200	-2.13766100	-0.22575600
C	3.42661300	0.06202000	0.07374200
H	3.58023400	1.11394200	-0.17908300
H	4.31318100	-0.27704400	0.61541700

H	3.36770300	-0.50109900	-0.86044100

TS27

C	1.44942500	0.46967000	-0.89409000
C	0.04605100	0.13898000	-1.28890800
C	-1.03893100	0.26002200	-0.41294400
C	-0.88862800	1.11485700	0.83123200
C	0.55836600	1.32845200	1.30439000
C	1.52086200	1.58737800	0.14366000
H	2.04510500	0.72507300	-1.77815000
H	-1.34927000	2.08545700	0.59730200
H	-1.48164300	0.67945900	1.63757200
H	0.58759400	2.16584800	2.00780400
H	0.88722000	0.44518500	1.85152500
H	1.26928600	2.52607500	-0.36512500
H	2.54932400	1.67914400	0.49913900
C	-2.43247100	0.15353700	-1.01471600
H	-2.42271200	-0.67467200	-1.73006100
H	-2.60074000	1.06466900	-1.60364300
O	-0.97384800	-1.54500900	0.20723800
O	-0.16724300	-1.70265800	1.33331500
H	-0.10465200	-0.36029700	-2.23990400
H	0.71081000	-1.85418900	0.94078500
O	1.98603800	-0.79039500	-0.37975300
O	3.40514600	-0.61559500	-0.09801000
H	3.78893000	-1.10850500	-0.83672000
C	-3.58584000	-0.03482500	-0.02523200
H	-3.70296800	0.82738700	0.63596000
H	-4.52629300	-0.15901900	-0.56838400
H	-3.42373400	-0.92078700	0.58924100

P27

C	-1.09510300	-0.44700000	0.56829200
C	0.37012500	-0.68454100	0.32397200
C	1.23799900	0.27992300	-0.00140000
C	0.79581000	1.71803900	-0.17514300
C	-0.71921200	1.86489200	-0.34769700
C	-1.45638000	1.03194400	0.70198400
H	-1.41909200	-0.99902600	1.45790200
H	1.13241400	2.29568300	0.69820700
H	1.32055500	2.15330600	-1.03358400
H	-1.00808900	2.91803100	-0.28450300
H	-1.00417000	1.51118400	-1.34424400
H	-1.18193000	1.36929700	1.70887000
H	-2.54090500	1.13627200	0.61839500
C	2.71660500	0.03809400	-0.20582300

H	2.96711400	0.34353100	-1.23083100
H	3.26392000	0.74144000	0.43713800
H	0.69683900	-1.71363000	0.41890800
O	-1.74850500	-1.06274700	-0.56477900
O	-3.15849600	-1.22627000	-0.21429300
H	-3.56847900	-0.73951800	-0.94100000
C	3.23140000	-1.38018400	0.03972700
H	4.30994100	-1.42666400	-0.13010700
H	3.04135700	-1.70376800	1.06651900
H	2.75907000	-2.10100500	-0.63257200

R28

C	2.27041800	1.39523700	0.46276400
C	0.75116200	1.34482800	0.66818200
C	2.10099000	-0.70553100	-0.90673000
C	2.85341200	0.00399500	0.16614200
H	0.52313000	0.76937400	1.56842100
H	0.37838000	2.35850300	0.83802200
H	2.49857600	2.06717600	-0.37367000
H	2.75230800	1.82044300	1.34804600
H	-0.47906000	-1.21565300	1.40531300
H	2.58486700	-1.50114600	-1.46332000
H	2.80460700	-0.60280200	1.08639400
H	3.91431800	0.07709400	-0.09451300
C	-1.50682400	0.71651900	-0.45043400
H	-1.91354600	0.27703800	-1.36412800
O	0.00494900	-1.78907100	-0.31724200
O	0.28480300	-1.74651400	1.10672700
C	0.03366500	0.73608200	-0.55498800
H	0.25243500	1.39985200	-1.40404600
C	0.61770100	-0.63215800	-0.99041000
H	0.28533000	-0.84581300	-2.01395000
O	-1.94123600	-0.14972000	0.63544800
O	-2.67198400	-1.27615900	0.09361000
H	-1.92565800	-1.83960300	-0.19246900
C	-2.14540800	2.08358700	-0.22503700
H	-1.81665700	2.79861900	-0.98470300
H	-3.23135000	1.99121600	-0.27924800
H	-1.89062400	2.48206500	0.75858100

TS28

C	1.83206100	-1.93806200	-0.57856100
C	0.37181600	-1.50274500	-0.75784100
C	2.13545700	0.19764300	0.74694400
C	2.74689900	-0.75131800	-0.23587000

H	0.27656700	-0.80752800	-1.59625700
H	-0.24500800	-2.37211900	-0.99838900
H	1.88811900	-2.67960000	0.22721600
H	2.19492000	-2.43367600	-1.48347400
H	1.54829500	3.22565500	-0.16731800
H	2.78365900	0.92915200	1.22045900
H	2.97603200	-0.18607400	-1.15320300
H	3.71630800	-1.10185700	0.13615200
C	-1.67206400	-0.49124900	0.47937600
H	-1.90914700	0.21767100	1.27783400
O	0.47461400	1.71365900	-0.42042700
O	0.70282300	2.95088000	0.21421400
C	-0.15469500	-0.81880200	0.51598000
H	-0.05185900	-1.53945600	1.34358600
C	0.76298300	0.31684200	0.92599600
H	0.41008800	0.96194300	1.72388800
O	-2.07858700	0.11154300	-0.75579600
O	-2.21738300	1.53826700	-0.58244300
H	-1.27839100	1.81203000	-0.64955700
C	-2.53631800	-1.74277900	0.64253100
H	-2.29453600	-2.26830500	1.57047500
H	-3.58861800	-1.45659700	0.66508000
H	-2.39500300	-2.43320400	-0.19221500

P28

C	-2.31186900	1.05593600	-0.42504300
C	-0.78169400	1.09899000	-0.51941400
C	-0.13374500	0.35700600	0.66622400
C	-0.82884000	-0.96194000	0.92833100
C	-2.01764900	-1.29476100	0.42805500
C	-2.83185400	-0.38749800	-0.45747600
H	-0.43792300	2.13647900	-0.54873800
H	-2.75715400	1.63673900	-1.23841900
H	-2.43686000	-2.27102100	0.65824500
H	-3.88408900	-0.41920900	-0.15202800
C	1.39071300	0.15957200	0.52012200
H	-0.30524800	-1.66538700	1.57222600
H	1.73827000	-0.44541500	1.36878600
H	-0.24545400	0.98041400	1.56679600
H	-0.45875700	0.62783000	-1.45265000
H	-2.62922200	1.53100700	0.51124900
H	-2.81198500	-0.77352500	-1.48633600
O	1.56308100	-0.62284000	-0.67106400
O	2.94694900	-1.06491500	-0.71924100
H	2.81856000	-2.01710400	-0.61188400

C	2.19952500	1.45079600	0.45881100
H	2.06423800	2.03063800	1.37650700
H	3.25940300	1.21690900	0.35299900
H	1.89846000	2.06508400	-0.39196100

R29

C	0.16892000	1.78541400	0.01517200
C	-1.31224300	1.78796200	0.41556500
C	-0.22062400	-0.39963600	-1.11851700
C	0.73315600	0.37544000	-0.26172000
H	-1.43434600	1.30165300	1.38671800
H	-1.65624800	2.82043700	0.52968200
H	0.30559000	2.37880800	-0.89613100
H	0.78396400	2.25496500	0.78780200
H	-0.55786100	-2.09873000	0.89479900
H	0.16421800	-1.03179600	-1.90790200
O	-2.02922000	-1.04187400	0.40693200
O	-1.38828300	-2.33856300	0.44950700
C	-2.18743600	1.05780600	-0.61223100
H	-2.16821400	1.58147500	-1.57436000
C	-1.68918400	-0.37858800	-0.83511400
H	-2.23594500	-0.86782400	-1.64811800
H	-3.22958400	1.02076400	-0.28250800
C	2.15250000	0.44838200	-0.85018300
H	2.07496000	0.91684600	-1.83760600
O	0.74821400	-0.41889000	0.97897200
O	1.64803600	0.18769100	1.95334800
H	1.00886100	0.49741800	2.60963200
C	2.88057700	-0.89404200	-0.95370300
H	2.34818700	-1.60682900	-1.58929200
H	3.87522100	-0.75123300	-1.38387900
H	2.99863600	-1.34756500	0.03129900
H	2.73858600	1.12826500	-0.22583200

TS29

C	-1.11754000	1.48791900	-0.24848800
C	0.19470000	2.01226600	-0.84901800
C	0.23898600	-0.14080000	1.07951100
C	-0.98361000	0.02410900	0.21117200
H	0.42262000	1.43425400	-1.74673900
H	0.07144200	3.05384100	-1.15954600
H	-1.42029200	2.09210700	0.61471200
H	-1.91863800	1.55640000	-0.98801100
H	3.08595400	-2.08267700	0.34468600
H	0.24647700	-0.96132700	1.78857200

O	2.26431500	-0.56182100	-0.39760500
O	3.36336200	-1.16110500	0.23684600
C	1.36494500	1.90436100	0.13604000
H	1.27957800	2.68520900	0.90546100
C	1.40504100	0.58765100	0.86702900
H	2.20680900	0.47231000	1.58839800
C	-2.22318900	-0.53839200	0.92468300
H	-2.01002400	-1.59010900	1.14128200
H	-2.32383800	-0.03214600	1.89243200
H	2.32100300	2.06769100	-0.36637800
O	-0.79019500	-0.67142300	-1.06141300
O	-0.23539700	-1.98999700	-0.83642300
H	0.71324700	-1.76286100	-0.86807800
C	-3.52869300	-0.43752600	0.13280500
H	-4.32994200	-0.96921400	0.65225700
H	-3.85372500	0.59811500	0.00356100
H	-3.41118900	-0.88435800	-0.85624900

P29

C	-1.78241300	-0.86475900	-0.97757900
C	-0.39732800	-1.23752700	-0.43847000
C	0.34894500	-0.02295500	0.13917900
C	-0.58042400	0.85393600	0.95471300
C	-1.90839100	0.73265800	0.96003700
C	-2.66283900	-0.26542700	0.12435400
H	0.21253700	-1.68187800	-1.22820600
H	-2.26522600	-1.74383800	-1.41420700
H	-2.49580000	1.37627900	1.61098400
H	-3.54911400	0.21172600	-0.30879900
C	1.57762800	-0.41243700	0.98247000
H	-0.10423100	1.59688600	1.58727200
H	2.05438500	0.51708400	1.30591400
H	1.21876900	-0.91297500	1.88904500
H	-0.49164200	-1.98532000	0.35727400
H	-1.65941800	-0.13347400	-1.78165200
H	-3.04906000	-1.05887900	0.78071600
O	0.78448900	0.67264800	-1.06954200
O	1.41512900	1.92809800	-0.70824800
H	0.68469600	2.54435800	-0.85842300
C	2.60752700	-1.28614900	0.26174300
H	3.48937800	-1.43032400	0.89139100
H	2.21226600	-2.27636400	0.02044400
H	2.92928500	-0.81329800	-0.66811800

R30

C	1.12368700	1.54999600	-0.01068900
C	0.90407300	0.05039500	0.25196000
C	-1.40196800	1.82856100	-0.00513800
C	-0.11224400	2.21710800	-0.64284500
H	1.36417700	2.02535000	0.94617600
H	1.98439900	1.69068000	-0.66703900
H	-3.73687500	-1.36076000	0.10866400
H	-2.28457400	2.43129900	-0.19171800
H	-0.14897800	1.93747300	-1.70730500
H	0.00015300	3.30723100	-0.62840700
O	-2.22060000	-0.34640000	-0.39302500
O	-2.79790400	-1.51711300	0.27861300
C	-0.31810100	-0.13083600	1.17198200
H	-0.08316100	0.32879800	2.13781600
C	-1.60226300	0.50296400	0.63796000
H	-2.34672300	0.57130900	1.43887500
C	2.12875600	-0.65882400	0.85731300
H	2.30691300	-0.25130200	1.85940300
H	1.85158900	-1.70854000	0.99026800
H	-0.49709800	-1.19013000	1.36134400
O	0.64312100	-0.45304000	-1.07925300
O	0.17460400	-1.82095700	-1.01777400
H	-0.78485800	-1.66101500	-1.04569900
C	3.40683500	-0.57943100	0.01821400
H	3.80804200	0.43636700	-0.03175600
H	4.18404200	-1.21566000	0.44941300
H	3.21653900	-0.92061600	-1.00106800

TS30

C	1.16971200	1.52931300	-0.15061800
C	0.96796700	0.05882300	0.25547400
C	-1.34467600	1.81503200	0.00392500
C	-0.10290800	2.14828500	-0.75596200
H	1.47013900	2.08595300	0.74342200
H	1.98780500	1.60565600	-0.86904900
H	-4.06021300	-0.85233600	-0.20230400
H	-2.23293400	2.41275600	-0.17487800
H	-0.22486200	1.78416800	-1.78612500
H	0.00573700	3.23578200	-0.83649900
O	-2.22228300	-0.59260400	-0.44366200
O	-3.28109800	-1.25027400	0.21186600
C	-0.20315900	-0.04153300	1.25121600
H	0.11907900	0.39472100	2.20529500
C	-1.44635000	0.68021400	0.79920600
H	-2.31012700	0.58749100	1.44763600

H	-0.43115500	-1.08858600	1.45300400
C	2.22656200	-0.59689200	0.85173900
H	1.95495800	-1.62281200	1.11637700
H	2.47568800	-0.08468600	1.78857700
O	0.62897400	-0.55936200	-1.00567200
O	0.18515200	-1.92166500	-0.79716800
H	-0.77906500	-1.75834000	-0.74865000
C	3.44382900	-0.62503600	-0.07623500
H	3.84023600	0.37516000	-0.27091700
H	4.24893700	-1.21272800	0.37226100
H	3.18395500	-1.08019100	-1.03353600

P30

C	0.79075500	1.40270000	0.34341300
C	-0.30970700	0.37265600	0.01812300
C	0.11846400	-0.48532100	-1.18231700
C	1.57014000	-0.89162300	-1.15145800
C	2.47510200	-0.34750100	-0.33946600
C	2.14881900	0.75046700	0.63611200
H	0.47101600	2.01622000	1.19027200
H	3.50104000	-0.70567800	-0.36193200
H	2.93428600	1.51447600	0.61035500
H	1.87049700	-1.67582600	-1.84181400
H	-0.09090900	0.07403500	-2.10358200
H	0.87827000	2.06671600	-0.52249900
H	2.15675300	0.34494700	1.65442500
H	-0.51767300	-1.37221600	-1.22789200
C	-1.65600800	1.08871900	-0.19746000
H	-1.89003800	1.60972700	0.73659700
H	-1.49643900	1.86549000	-0.95532300
O	-0.33152400	-0.40578200	1.24631400
O	-1.11948700	-1.61723600	1.09651300
H	-0.41682400	-2.28038000	1.13661000
C	-2.85359100	0.22491600	-0.61043600
H	-3.74156400	0.85315200	-0.72397200
H	-2.68550200	-0.27619800	-1.56757100
H	-3.06866500	-0.54040600	0.13463800

R31

C	-1.44349200	-0.13654800	0.41608400
C	-0.23682900	0.75994900	0.75015100
C	0.22021500	-2.00074600	-0.06083800
C	-1.01638900	-1.29940500	-0.51388800
H	-1.75524600	-0.57404500	1.37461100
H	3.86798900	-0.26846500	-0.92486800

H	0.37325000	-3.04383200	-0.31302100
H	-0.84326700	-0.88211300	-1.51927800
H	-1.83234900	-2.01956900	-0.61788700
O	2.05470600	-0.77466500	-0.73379500
O	3.18910700	0.08617900	-0.33577500
C	0.92017400	-0.04639000	1.34941500
H	0.59117900	-0.45369400	2.31174100
C	1.37257400	-1.21886900	0.47294700
H	2.06817100	-1.85767300	1.02936600
H	1.76565300	0.60855900	1.55894500
H	-0.54170300	1.53333600	1.46767100
C	-2.62765600	0.67325300	-0.14031400
H	-2.79162800	1.53178700	0.52105400
H	-2.35046400	1.09086300	-1.11213700
O	0.11318700	1.39906400	-0.48302100
O	1.23897900	2.27982300	-0.25679900
H	1.98046300	1.70151800	-0.51122100
C	-3.93345000	-0.11940200	-0.25997400
H	-4.75097600	0.52949000	-0.58495600
H	-3.85738700	-0.93203900	-0.98727100
H	-4.22240000	-0.55837600	0.70087500

TS31

C	-1.52875100	-0.17330300	0.41446400
C	-0.41506400	0.83690400	0.74683600
C	0.37354600	-1.80153800	-0.06788100
C	-0.96859900	-1.29361800	-0.49042600
H	-1.81715000	-0.62584600	1.37317100
H	3.96119500	-1.08581300	-1.00698700
H	0.70872700	-2.74971600	-0.47691500
H	-0.88481300	-0.91362200	-1.51999300
H	-1.67253600	-2.13087500	-0.54027600
O	2.34206400	-0.20967900	-0.67147800
O	3.69627200	-0.42382800	-0.35239400
C	0.75747700	0.15058800	1.45453400
H	0.41697900	-0.17374700	2.44654000
C	1.25953400	-1.05038300	0.69557400
H	2.13251300	-1.54747800	1.10252000
H	1.56699900	0.86088300	1.62125000
C	-2.77233400	0.51149300	-0.17850200
H	-3.05261700	1.33814300	0.48434400
H	-2.50450100	0.96832600	-1.13544200
H	-0.80572700	1.63972400	1.38529600
O	-0.04502100	1.38684900	-0.52186600
O	1.09125500	2.26698500	-0.36372500

H	1.80487400	1.62807000	-0.56282800
C	-3.97900800	-0.41579100	-0.35615200
H	-4.84945000	0.14511800	-0.70619000
H	-3.78758500	-1.20513200	-1.08795400
H	-4.25428000	-0.89677100	0.58848000

P31

C	-0.54457400	-0.55830400	0.60278500
C	0.80714000	0.14506600	0.38843600
C	0.68117300	1.22065200	-0.70850000
C	-0.56768500	2.05196400	-0.57073700
C	-1.60117100	1.70543300	0.19458400
C	-1.64515700	0.43060500	0.99649500
H	-0.44451400	-1.33619800	1.36829000
H	-2.46763100	2.35857300	0.25678500
H	-2.61428600	-0.06538100	0.88490400
H	-0.60858600	2.97772000	-1.13884600
H	0.69459100	0.74325000	-1.69737000
H	1.01173500	0.66267300	1.33591200
H	-1.55564800	0.65453800	2.06822600
H	1.55630300	1.87808100	-0.68154200
C	1.94744500	-0.85627300	0.13692400
H	1.91131100	-1.62087100	0.92181400
H	1.76055100	-1.38028600	-0.80480900
O	-0.82712300	-1.20850900	-0.64642100
O	-1.96300200	-2.09368500	-0.44550500
H	-2.58505500	-1.71801200	-1.08276600
C	3.34466200	-0.22747600	0.11921900
H	4.11425700	-0.99655200	0.01237200
H	3.46891900	0.47337400	-0.71038000
H	3.54805500	0.31664200	1.04758700

R32

C	1.39459500	-0.14064800	0.39586500
C	0.92336200	-1.26205200	-0.55096000
C	-0.95051200	-0.06611900	1.29087700
C	0.19835100	0.75451500	0.80589300
H	0.58983700	-0.81897600	-1.49264300
H	1.72604400	-0.60501200	1.33562100
H	-2.68839600	0.90914300	-0.48268900
H	-1.49157500	0.23606700	2.17805300
H	0.50868700	1.47895600	1.56738500
O	-2.02755200	-0.83740000	-0.76078500
O	-3.13726400	0.04437100	-0.46430000
C	-0.21588700	-2.10068800	0.04787600

H	0.14134300	-2.64997000	0.92609300
C	-1.40979900	-1.23824300	0.48510100
H	-2.13622600	-1.83205200	1.04967800
H	-0.56856200	-2.84165600	-0.67530100
H	1.76422300	-1.92116400	-0.78348500
C	2.57078900	0.68555200	-0.15793000
H	2.72026700	1.55511700	0.49302100
H	2.30119800	1.08470900	-1.14012000
O	-0.12417400	1.49362100	-0.39958200
O	-1.31316800	2.29762000	-0.10451800
H	-1.13321400	3.05337500	-0.67975500
C	3.88701800	-0.09296100	-0.25449200
H	3.82004800	-0.92713500	-0.95732300
H	4.69663000	0.55667400	-0.59743700
H	4.17945400	-0.49989200	0.71900100

TS32

C	1.49976000	-0.18563200	0.39309600
C	0.89415800	-1.19786300	-0.59884200
C	-0.82231700	0.16047900	1.30099100
C	0.41528300	0.83856700	0.78556900
H	0.61109800	-0.66427300	-1.50933100
H	1.76090200	-0.71534800	1.32075800
H	-4.03181900	0.37491700	-0.13285300
H	-1.44197400	0.68775500	2.01637300
H	0.79634100	1.54623700	1.53136600
O	-2.35235000	-0.27374200	-0.67746800
O	-3.69971300	-0.50537300	-0.36239400
C	-0.33389000	-1.92019400	-0.02428600
H	-0.00920500	-2.68667000	0.69390400
C	-1.28217800	-1.01805100	0.72242600
H	-2.13537400	-1.50708300	1.17984100
H	-0.88129900	-2.44696000	-0.80945200
C	2.77313100	0.50444400	-0.13018200
H	2.55192700	0.99074200	-1.08438000
H	3.03727900	1.30973300	0.56522300
H	1.64153300	-1.94321600	-0.88160300
O	0.14930500	1.56357000	-0.44092100
O	-1.07059500	2.33008900	-0.30652600
H	-1.70543300	1.66056700	-0.62345100
C	3.97543200	-0.43295100	-0.28273400
H	3.80099400	-1.20778800	-1.03365100
H	4.86304000	0.12457800	-0.59301200
H	4.21255000	-0.93233700	0.66251700

P32

C	0.66344300	1.19269200	0.75516600
C	0.76877600	0.11233800	-0.33474400
C	-0.61434200	-0.52384400	-0.56540500
C	-1.71224000	0.50573100	-0.68204600
C	-1.56730500	1.76891400	-0.28064000
C	-0.30735600	2.30706800	0.34110500
H	1.64574500	1.62102500	0.96950100
H	-2.38910000	2.46875000	-0.41417300
H	-0.56253800	2.93181200	1.20474500
H	-2.64768600	0.17580600	-1.12215400
H	1.01399300	0.61191600	-1.28302900
H	0.30925200	0.72289400	1.67855100
H	0.17838300	2.98533500	-0.37544700
H	-0.58742700	-1.14440000	-1.46915600
C	1.85727000	-0.94263600	-0.06609700
H	1.72006400	-1.76978200	-0.77170600
H	1.70779200	-1.37239800	0.92831800
O	-0.83396900	-1.41378800	0.55228300
O	-2.03585500	-2.17959300	0.28150600
H	-2.66152900	-1.74722900	0.87915100
C	3.28667700	-0.40676200	-0.19888800
H	3.50164200	0.37977800	0.52949900
H	4.01634900	-1.20487300	-0.03829900
H	3.46411400	0.00741800	-1.19682600

R33

C	-2.04137500	0.25472900	0.85701500
C	-0.61291100	-0.10803800	1.11088400
C	0.45870400	0.20491100	0.10933500
C	0.25987900	1.62853600	-0.46174700
C	-1.19464300	1.92757100	-0.85086700
C	-2.16597300	1.68354500	0.31048700
H	-2.63046900	0.13117200	1.77190900
H	0.59735100	2.33219700	0.30784200
H	0.91629500	1.76015700	-1.32580100
H	-1.26997800	2.96736000	-1.18408800
H	-1.47972400	1.29659400	-1.69487700
H	-1.96090600	2.37944000	1.13186900
H	-3.19990400	1.84843800	-0.00506600
C	1.85543500	-0.02864700	0.71009400
H	1.89566500	-1.07249200	1.03859500
H	1.93765600	0.58396000	1.61587300
O	0.29131900	-0.61225000	-1.09773000
O	0.20998000	-2.01943100	-0.68972800

H	-0.35846300	-0.70777100	1.97644100
H	0.63305200	-2.42601000	-1.45751900
O	-2.64178700	-0.56570900	-0.17443900
O	-2.53847600	-1.95511800	0.21903200
H	-1.68658000	-2.18992400	-0.19176800
C	3.04167700	0.25364800	-0.21815800
H	3.07060700	1.31625700	-0.48029600
H	2.89891500	-0.29051300	-1.15691000
C	4.38048200	-0.14401100	0.41131600
H	5.21444100	0.06511800	-0.26393400
H	4.56134400	0.40456400	1.34103000
H	4.40475900	-1.21195200	0.64945400

TS33

C	1.79676400	0.42434200	-0.95938200
C	0.36424300	0.11239700	-1.25145300
C	-0.65870100	0.28201900	-0.31079600
C	-0.40960900	1.17026600	0.89367100
C	1.06927000	1.36567300	1.26535000
C	1.95891600	1.56943200	0.03743700
H	2.33727800	0.64168700	-1.88796300
H	-0.86485400	2.14336200	0.65987800
H	-0.95725600	0.77263100	1.75001400
H	1.16108600	2.22238500	1.93951200
H	1.41535700	0.49168800	1.81675800
H	1.69419700	2.49868500	-0.48192100
H	3.01012800	1.64811400	0.32255000
C	-2.08936400	0.18685500	-0.81865300
H	-2.14395300	-0.66622600	-1.50382100
H	-2.27917100	1.08070200	-1.42893900
O	-0.58653700	-1.50519700	0.35891000
O	0.29178700	-1.64762500	1.43225300
H	0.14169800	-0.41047300	-2.17524500
H	1.13826300	-1.82749700	0.98593700
O	2.34192200	-0.83177800	-0.44433900
O	3.77969400	-0.67940100	-0.26172200
H	4.10402800	-1.20062900	-1.00943700
C	-3.19124400	0.05788800	0.24028100
H	-3.20256100	0.94770200	0.87828500
H	-2.96066700	-0.79231500	0.88583500
C	-4.57491000	-0.12297600	-0.39117200
H	-4.61459000	-1.02902300	-1.00371400
H	-5.35020600	-0.20755700	0.37480800
H	-4.83590100	0.72340000	-1.03504600

P33

C	-1.50794700	-0.51651300	0.57052100
C	-0.02028000	-0.55033700	0.34980700
C	0.71005200	0.52193200	0.02335200
C	0.07378100	1.88205000	-0.17612100
C	-1.44404000	1.81423800	-0.37131100
C	-2.07415100	0.89880500	0.67934200
H	-1.76569300	-1.09837500	1.46277400
H	0.31317500	2.51114600	0.69373700
H	0.54582700	2.37631400	-1.03317300
H	-1.87761800	2.81751200	-0.32637000
H	-1.66168100	1.41280000	-1.36675600
H	-1.86516900	1.28271600	1.68527600
H	-3.16120500	0.84964500	0.57941500
C	2.21079400	0.48915100	-0.15773000
H	2.43260900	0.81095000	-1.18540500
H	2.64462900	1.27374300	0.47959700
H	0.44428200	-1.52290100	0.46328000
O	-2.05126900	-1.23028800	-0.56329000
O	-3.42971300	-1.58601400	-0.23015500
H	-3.89269200	-1.16823100	-0.96786700
C	2.92981000	-0.83366200	0.11760600
H	2.72215400	-1.15611900	1.14362700
H	2.52760500	-1.61268900	-0.53858600
C	4.44403400	-0.72918100	-0.08823400
H	4.88513700	0.01879200	0.57813000
H	4.93783900	-1.68359500	0.11221300
H	4.68634700	-0.44018900	-1.11571900

R34

C	2.20346300	-1.81525200	-0.45805000
C	0.75042900	-1.38932900	-0.70355900
C	2.50739100	0.23376700	0.96496700
C	3.09951200	-0.61673500	-0.10577000
H	0.69989300	-0.75864000	-1.59419200
H	0.14695200	-2.27711700	-0.90999900
H	2.23169700	-2.53991200	0.36492100
H	2.59604200	-2.32739400	-1.34143300
H	0.20238800	1.40767800	-1.40661200
H	3.15206400	0.87390500	1.55790700
H	3.23312500	0.00212800	-1.00945000
H	4.10038200	-0.95404900	0.18286900
C	-1.32435900	-0.25440300	0.37186700
H	-1.64947200	0.23716700	1.29304500
O	0.76780500	1.81233900	0.33921700

O	1.06674800	1.72316100	-1.07854200
C	0.16274800	-0.64640500	0.51533600
H	0.18764400	-1.35898700	1.35252500
C	1.04970700	0.52788800	1.00186700
H	0.74423000	0.80060700	2.01969800
O	-1.50531600	0.72722600	-0.68736400
O	-1.92995000	1.99033800	-0.11853800
H	-1.06788800	2.33504200	0.18975800
C	-2.26385000	-1.42625900	0.07334400
H	-2.02423800	-2.24911600	0.75691300
H	-2.06411200	-1.79036900	-0.93873900
C	-3.74043500	-1.04444100	0.19894500
H	-3.97675300	-0.20627100	-0.45922400
H	-4.38527300	-1.88407500	-0.07238200
H	-3.98859700	-0.74809900	1.22253000

TS34

C	1.77791100	-2.23368800	-0.55530100
C	0.45078000	-1.50810900	-0.81214400
C	2.42282000	-0.22313700	0.84000100
C	2.89030700	-1.26156400	-0.13118400
H	0.55237600	-0.79989900	-1.63884600
H	-0.31315500	-2.23090600	-1.10901400
H	1.63156700	-2.97938200	0.23517900
H	2.08938900	-2.78340600	-1.44804200
H	2.52184600	2.88087000	0.00007900
H	3.17414200	0.35183200	1.37331900
H	3.28334100	-0.74152600	-1.01922900
H	3.74508000	-1.80588000	0.28601300
C	-1.41529600	-0.10244500	0.31552900
H	-1.55112700	0.63808200	1.11013600
O	1.18306600	1.62662100	-0.37132800
O	1.61451700	2.77672500	0.31916500
C	-0.00773500	-0.74378400	0.44247000
H	-0.11384000	-1.47935900	1.25628500
C	1.09454000	0.17103400	0.94087100
H	0.82960500	0.85919600	1.73681400
O	-1.59244000	0.57965100	-0.93319200
O	-1.46764000	2.00558200	-0.74139800
H	-0.49088400	2.08991400	-0.72585400
C	-2.54151700	-1.14321300	0.40370600
H	-2.37088600	-1.76847200	1.28745000
H	-2.48199400	-1.80559500	-0.46617200
C	-3.93092500	-0.50617600	0.46917600
H	-4.09351000	0.14097300	-0.39400800

H	-4.71226600	-1.27065000	0.47947500
H	-4.04502400	0.10206900	1.37169900

P34

C	2.56138800	-1.21428000	-0.42233700
C	1.03973700	-1.10431000	-0.57808700
C	0.40954100	-0.36274200	0.61779100
C	1.21788900	0.86326800	0.98344400
C	2.45363600	1.10573200	0.54864200
C	3.21567500	0.17215500	-0.35572900
H	0.60043700	-2.10045700	-0.67857800
H	2.98520400	-1.79333000	-1.24834200
H	2.95504000	2.02085700	0.85383300
H	4.25235200	0.08721900	-0.01001000
C	-1.07601000	0.00609500	0.40198100
H	0.73945100	1.57673900	1.65142900
H	-1.40234900	0.62515000	1.24562600
H	0.41329300	-1.03912400	1.48619100
H	0.80662200	-0.55670700	-1.49679100
H	2.79122600	-1.76432400	0.49829000
H	3.27564000	0.61086000	-1.36167300
O	-1.11109300	0.84073400	-0.77287500
O	-2.19672500	1.79868800	-0.63919500
H	-1.68658300	2.62005700	-0.62211100
C	-2.00691600	-1.20153800	0.25992500
H	-1.82151700	-1.87126800	1.10879500
H	-1.72678600	-1.75304300	-0.64265800
C	-3.49612400	-0.84789000	0.20189200
H	-3.71547200	-0.20407200	-0.65071900
H	-4.10236800	-1.75351000	0.11267700
H	-3.81454700	-0.31935800	1.10547400

R35

C	0.35457400	-1.81756100	-0.08552900
C	1.87627300	-1.70828200	0.07768500
C	0.36668600	0.48960100	-1.02613500
C	-0.36468500	-0.45154700	-0.11820500
H	2.11485300	-1.30366400	1.06453800
H	2.32007500	-2.70715700	0.02596700
H	0.12012800	-2.33480200	-1.02292100
H	-0.08324200	-2.41588300	0.71825100
H	0.88587700	2.02247700	1.08052600
H	-0.19419800	1.14341700	-1.68080000
O	2.34092600	1.16844500	0.26011100
O	1.60831100	2.38460200	0.53980400

C	2.50616500	-0.80245800	-0.98915400
H	2.37380300	-1.23469100	-1.98711700
C	1.85826100	0.59096900	-0.97740500
H	2.22033500	1.20508300	-1.80870700
H	3.58135500	-0.69114000	-0.82256400
C	-1.84986600	-0.61337800	-0.48201500
H	-1.89865700	-0.97251100	-1.51712600
O	-0.23908100	0.21882800	1.18815000
O	-0.91317300	-0.56081000	2.21962200
H	-0.15147500	-0.86760900	2.73040000
C	-2.71069000	0.64497000	-0.32283900
H	-2.30134500	1.46365100	-0.92479400
H	-2.66386200	0.97647200	0.71723900
H	-2.26560800	-1.40876800	0.14426500
C	-4.16887600	0.40353300	-0.72530200
H	-4.62095900	-0.38600200	-0.11708300
H	-4.76994300	1.30713600	-0.59406000
H	-4.25005900	0.10039400	-1.77411700

TS35

C	-0.52018300	1.67914300	-0.28650200
C	0.88531700	2.06269000	-0.77119800
C	0.53186800	-0.16286900	1.03867600
C	-0.59756700	0.19075500	0.10266300
H	1.10555000	1.50070600	-1.68093700
H	0.91098700	3.12410600	-1.03433800
H	-0.80869400	2.27712500	0.58581900
H	-1.25214600	1.87972400	-1.07215200
H	3.16514400	-2.40842600	0.39286200
H	0.38973700	-1.00876300	1.70223100
O	2.59358200	-0.76445800	-0.32114600
O	3.56082200	-1.52538200	0.35344900
C	1.96042900	1.76761200	0.28152700
H	1.91460500	2.51811300	1.08381200
C	1.78919400	0.42482900	0.94274000
H	2.51706900	0.17926600	1.70851700
C	-1.94190000	-0.24633500	0.70468000
H	-1.87543300	-1.32747700	0.87114900
H	-2.04690200	0.21717200	1.69421800
H	2.96275900	1.83361900	-0.14757400
O	-0.39891000	-0.46604100	-1.19008000
O	-0.02143400	-1.85100200	-0.99976600
H	0.94727700	-1.73995600	-0.95648500
C	-3.17830800	0.05350900	-0.14927600
H	-3.31897300	1.13583900	-0.24094000

H	-3.00646600	-0.32754300	-1.15977200
C	-4.45135900	-0.56969900	0.43145600
H	-4.65627600	-0.19458200	1.43948200
H	-5.32219100	-0.34309600	-0.18951600
H	-4.36347900	-1.65857100	0.49536400

P35

C	-2.11105800	-0.96143200	-0.99746200
C	-0.62339200	-1.12573000	-0.66885100
C	-0.04440100	0.10764900	0.04548300
C	-1.00743000	0.63772400	1.08953500
C	-2.27853600	0.24988600	1.19920100
C	-2.93746900	-0.73602200	0.27327800
H	-0.04889900	-1.30586100	-1.58022000
H	-2.47599200	-1.83976100	-1.53774500
H	-2.88570500	0.64998600	2.00808400
H	-3.94480400	-0.38705600	0.01978300
C	1.33125800	-0.15231700	0.68664300
H	-0.59891600	1.35263400	1.79724300
H	1.66865600	0.79301800	1.12356100
H	1.18967000	-0.84979200	1.52138600
H	-0.47555300	-1.99379700	-0.01631400
H	-2.22916700	-0.10245100	-1.66428000
H	-3.08288900	-1.68511000	0.80958000
O	0.09717000	1.06030500	-1.05354400
O	0.52893500	2.34362800	-0.53316600
H	-0.31548300	2.81416000	-0.49512900
C	2.41429400	-0.67853500	-0.26185600
H	2.13367700	-1.66585900	-0.64408700
H	2.47632100	-0.01436800	-1.12841800
C	3.78402900	-0.77476800	0.41726000
H	4.54333100	-1.15276000	-0.27281400
H	4.11741600	0.20481400	0.77338200
H	3.75573500	-1.44798000	1.28029000

R36

C	0.46573900	1.77854300	-0.03062000
C	0.51512100	0.25502800	0.17750800
C	-2.06693800	1.62628000	0.11350000
C	-0.89813400	2.24860500	-0.57015900
H	0.67800000	2.25454700	0.93257200
H	1.25189700	2.08521900	-0.72282300
H	-3.82616600	-1.91475400	0.20444400
H	-3.04678100	2.07744300	-0.00256700
H	-0.94804000	2.00368500	-1.64261800

H	-0.96834600	3.34090800	-0.51392700
O	-2.53227200	-0.64084300	-0.32754700
O	-2.86615100	-1.91523000	0.32004600
C	-0.60669700	-0.16116500	1.14837900
H	-0.39773400	0.29824700	2.12023300
C	-2.00671600	0.26449900	0.70724400
H	-2.70548800	0.17847700	1.54673300
C	1.87138800	-0.25930000	0.69143900
H	2.03246100	0.12896700	1.70502200
H	1.78289000	-1.34674100	0.78378400
H	-0.59548100	-1.24128400	1.30062800
O	0.26631600	-0.23904800	-1.15984900
O	0.03669600	-1.66763200	-1.13469700
H	-0.93597100	-1.67137800	-1.10703100
C	3.08238800	0.06515800	-0.19058100
H	3.25890500	1.14613500	-0.20925500
H	2.85721500	-0.23312800	-1.21818100
C	4.35568700	-0.63988500	0.28751400
H	5.20852300	-0.39522100	-0.35160500
H	4.61417500	-0.34859600	1.31095800
H	4.23221000	-1.72708700	0.27467100

TS36

C	0.53396700	1.75780300	-0.16670400
C	0.57476300	0.26087800	0.18868100
C	-1.98501300	1.65445800	0.10529100
C	-0.84465500	2.19690200	-0.69193800
H	0.79080500	2.32364500	0.73510300
H	1.29557200	1.98096100	-0.91587200
H	-4.27827100	-1.38147400	-0.09945600
H	-2.96021300	2.11650900	-0.01197700
H	-0.95992400	1.85329300	-1.72989300
H	-0.90378100	3.29041200	-0.73307400
O	-2.51167600	-0.84214300	-0.40234200
O	-3.43118100	-1.67167800	0.26815100
C	-0.52020200	-0.04816200	1.22787800
H	-0.22206800	0.40007800	2.18419000
C	-1.87758200	0.49135300	0.85761500
H	-2.68600500	0.24679600	1.53718800
H	-0.57881100	-1.12374700	1.39730300
C	1.94260000	-0.21788200	0.70640900
H	1.84122700	-1.28389400	0.93473900
H	2.15505800	0.28837900	1.65650700
O	0.27140100	-0.35942000	-1.08094100
O	0.04717100	-1.77929000	-0.90862400

H	-0.92712000	-1.76556400	-0.81181600
C	3.11820400	-0.03020900	-0.25922000
H	3.30487100	1.03630900	-0.42613100
H	2.84608600	-0.45573700	-1.22878100
C	4.40304900	-0.68697200	0.25493800
H	4.26770500	-1.76441000	0.38967800
H	5.23024000	-0.54164000	-0.44520500
H	4.70812500	-0.26869000	1.21984000

P36

C	1.03697600	-1.35887900	-0.57788600
C	0.06099100	-0.25042400	-0.13304400
C	0.49772800	0.31035300	1.22934500
C	1.98817700	0.49778200	1.35953700
C	2.87734900	-0.02297900	0.51543900
C	2.49026100	-0.87323400	-0.66396000
H	0.71076900	-1.76081600	-1.54111900
H	3.93647700	0.17081400	0.66400700
H	3.16071600	-1.73752600	-0.73350200
H	2.33153000	1.09627900	2.19958600
H	0.13852000	-0.36562900	2.01632300
H	0.96057200	-2.16958400	0.15368000
H	2.63917700	-0.30176600	-1.58738600
H	-0.01542900	1.25905800	1.40233400
C	-1.38211600	-0.78728700	-0.13592000
H	-1.60339800	-1.09801700	-1.16329100
H	-1.39595800	-1.70060700	0.47352700
O	0.25050100	0.72594500	-1.19394500
O	-0.37417900	1.99633300	-0.86818400
H	0.41322700	2.54217100	-0.73684500
C	-2.49632900	0.14824600	0.35419700
H	-2.29912900	0.45682600	1.38656300
H	-2.49727200	1.06029400	-0.24504800
C	-3.87310000	-0.52112000	0.28653700
H	-4.65928200	0.15287200	0.63750700
H	-4.12071300	-0.81274500	-0.73898000
H	-3.91001200	-1.42458700	0.90420800

R37

C	-0.99025500	-0.29797000	0.51862100
C	0.16259500	0.67769900	0.82025700
C	0.78111400	-2.01568500	-0.10118800
C	-0.52638200	-1.39778800	-0.46882000
H	-1.21706800	-0.78482000	1.47708300
H	4.24067900	0.01131600	-1.08840800

H	0.99784400	-3.03654400	-0.39431400
H	-0.43773200	-0.93971000	-1.46737100
H	-1.29046600	-2.17483700	-0.55551200
O	2.48214000	-0.63567900	-0.82446700
O	3.56855200	0.29715700	-0.45577500
C	1.40626100	-0.05476100	1.33471900
H	1.15939500	-0.51269400	2.29886000
C	1.89847400	-1.16436300	0.39970300
H	2.66751000	-1.76413700	0.90008700
H	2.21034900	0.65654200	1.52242300
H	-0.16098500	1.40525300	1.57644700
C	-2.25823600	0.43621100	0.04958200
H	-2.44583900	1.26749000	0.74076400
H	-2.06902900	0.89440600	-0.92619400
O	0.39893900	1.37577000	-0.40795800
O	1.46639600	2.33282500	-0.21070300
H	2.23451100	1.81994500	-0.52035700
C	-3.51726400	-0.43726400	-0.01914200
H	-3.37936200	-1.23969600	-0.75183000
H	-3.67071800	-0.93011400	0.94938000
C	-4.77075400	0.36258300	-0.38725200
H	-5.65481600	-0.27913900	-0.43452400
H	-4.96839800	1.14843800	0.34834200
H	-4.65740300	0.84574100	-1.36253100

TS37

C	-1.07208600	-0.25828200	0.54309300
C	0.02527000	0.78233200	0.83452900
C	0.85311200	-1.80810100	-0.08759400
C	-0.52833600	-1.33917000	-0.41846300
H	-1.28831200	-0.74065900	1.50610700
H	4.35015400	-0.94766600	-1.22056100
H	1.19542600	-2.73463400	-0.53823200
H	-0.51936200	-0.93546500	-1.44233200
H	-1.20485600	-2.19971500	-0.44581900
O	2.72694300	-0.13459200	-0.76682700
O	4.10419500	-0.31046600	-0.53472300
C	1.25986600	0.12196600	1.45636700
H	0.98924800	-0.23628500	2.45819000
C	1.75682600	-1.04316900	0.64051900
H	2.66884900	-1.51790900	0.98351300
H	2.05325500	0.85643300	1.59330900
C	-2.36942600	0.39707100	0.03943300
H	-2.63486600	1.20471500	0.73305200
H	-2.17646300	0.87839200	-0.92463000

H	-0.35384700	1.55682200	1.51359700
O	0.30166900	1.37251000	-0.43991600
O	1.41439200	2.28886500	-0.32639400
H	2.13650000	1.68028200	-0.58181500
C	-3.56457700	-0.55650000	-0.08321100
H	-3.34787800	-1.33802000	-0.81952600
H	-3.71817500	-1.07149800	0.87375100
C	-4.85490800	0.16361400	-0.48664400
H	-5.69197600	-0.53442100	-0.57195200
H	-5.12954600	0.92476200	0.24995400
H	-4.74018200	0.66576600	-1.45191100

P37

C	0.88046000	0.59400500	0.60383500
C	-0.32092900	-0.34416400	0.39230800
C	-0.00346800	-1.37819400	-0.70638800
C	1.37713600	-1.96641600	-0.57452200
C	2.33237600	-1.43750300	0.18806000
C	2.14475300	-0.17731900	0.99225600
H	0.64235300	1.33930100	1.37124300
H	3.30410600	-1.92081900	0.24602200
H	3.00629100	0.48788100	0.87860900
H	1.58489100	-2.86820000	-1.14469100
H	-0.10832500	-0.91111400	-1.69470900
H	-0.42404900	-0.89081600	1.33982900
H	2.10137400	-0.41526500	2.06383400
H	-0.74289400	-2.18509900	-0.67679000
C	-1.62472100	0.43341300	0.14556100
H	-1.72370600	1.19685200	0.92782300
H	-1.54374700	0.97913100	-0.80006900
O	1.03511500	1.28665800	-0.64490300
O	1.99004600	2.36482500	-0.44542500
H	2.66809700	2.11102100	-1.08574500
C	-2.89310500	-0.42894500	0.14007900
H	-2.84601500	-1.15944600	-0.67450800
H	-2.94126800	-1.01014300	1.06975100
C	-4.17163700	0.40158000	-0.00766100
H	-5.06209900	-0.23299000	-0.01398300
H	-4.27729000	1.11530400	0.81535000
H	-4.16419500	0.97379200	-0.94051000

R38

C	0.94228100	-0.30730900	0.50098600
C	0.43112000	-1.36183400	-0.50089200
C	-1.43446600	-0.05467200	1.27229000

C	-0.19617400	0.67636600	0.87175100
H	0.18866000	-0.86965900	-1.44601100
H	1.18037600	-0.82008200	1.44371700
H	-2.98487800	1.10723000	-0.56199500
H	-1.99485600	0.26993400	2.13932400
H	0.13131100	1.35397100	1.66833300
O	-2.45838400	-0.68221900	-0.85437300
O	-3.50491600	0.28364800	-0.59200600
C	-0.80599500	-2.11403200	0.01250300
H	-0.54524900	-2.71288000	0.89227800
C	-1.94572800	-1.16364300	0.41047200
H	-2.74923100	-1.70669500	0.91894600
H	-1.17917900	-2.80452600	-0.74932400
H	1.22500800	-2.08477500	-0.70722700
C	2.21136700	0.42988100	0.03433800
H	2.38971500	1.27801100	0.70781800
H	2.03721600	0.86204700	-0.95651700
O	-0.38864500	1.46755600	-0.32826300
O	-1.52007400	2.36354400	-0.07438900
H	-1.24521700	3.11447800	-0.61753100
C	3.47396000	-0.44119800	0.00306000
H	3.33815400	-1.27328000	-0.69553500
H	3.62588400	-0.89256900	0.99145300
C	4.72571700	0.34676100	-0.39468600
H	4.91825800	1.16477100	0.30654000
H	5.61181300	-0.29347100	-0.40969700
H	4.61586700	0.78534000	-1.39125600

TS38

C	1.04818600	-0.26110900	0.49841500
C	0.45810700	-1.23805700	-0.53779800
C	-1.30324500	0.14794400	1.29943000
C	-0.02087900	0.79227800	0.85488700
H	0.23602400	-0.68146900	-1.45139700
H	1.24602700	-0.81238500	1.42905100
H	-4.43122900	0.48836100	-0.28358900
H	-1.93933200	0.68485100	1.99290600
H	0.34613500	1.47602800	1.62946000
O	-2.74884700	-0.20697300	-0.75693100
O	-4.11670700	-0.39891000	-0.51099400
C	-0.81854900	-1.92807100	-0.03414900
H	-0.55356000	-2.71507200	0.68636800
C	-1.77238000	-1.00626100	0.68070800
H	-2.66202100	-1.47349200	1.08884100
H	-1.34399400	-2.42522400	-0.85291800

C	2.36646200	0.39477900	0.04837400
H	2.21365100	0.88896400	-0.91627000
H	2.61575500	1.19263400	0.75943500
H	1.19363100	-2.00319000	-0.79765200
O	-0.20354700	1.54341400	-0.37109200
O	-1.40332600	2.34753400	-0.28313600
H	-2.04321300	1.70381300	-0.64076000
C	3.55727600	-0.56799300	-0.04152700
H	3.35321200	-1.35052600	-0.77997900
H	3.68211300	-1.08020600	0.92088000
C	4.86294700	0.14101500	-0.41408600
H	5.12379500	0.90430400	0.32556500
H	5.69687000	-0.56365000	-0.47330000
H	4.77799600	0.63858800	-1.38493500

P38

C	0.08690300	-1.36623000	0.75679200
C	-0.27667800	-0.34512500	-0.33512700
C	0.91171400	0.60644600	-0.56554100
C	2.22629400	-0.12688000	-0.68013900
C	2.39103200	-1.38730200	-0.27771200
C	1.29814200	-2.21358900	0.34405900
H	-0.76292400	-2.01842500	0.97292500
H	3.35790200	-1.86753900	-0.40991900
H	1.69630100	-2.75735100	1.20840200
H	3.05450400	0.41947900	-1.11965600
H	-0.39183000	-0.89029600	-1.28288900
H	0.31758400	-0.82269900	1.67883500
H	0.99096800	-2.98974900	-0.37194500
H	0.73632900	1.20098300	-1.47016500
C	-1.58766400	0.41589700	-0.06850700
H	-1.64520200	1.26580000	-0.75954300
H	-1.55843500	0.85165100	0.93527400
O	0.90801700	1.52416000	0.55119700
O	1.88850800	2.55832000	0.27994900
H	2.59980900	2.29115600	0.87848200
C	-2.85373800	-0.43499300	-0.23061400
H	-2.82988600	-1.28225800	0.46340700
H	-2.87188400	-0.86716300	-1.23899100
C	-4.13867100	0.36549000	0.00269600
H	-4.21720400	1.20051900	-0.70030700
H	-5.02720100	-0.25939800	-0.12232000
H	-4.16502700	0.78315400	1.01379900

R39

C	-2.48564300	0.27006000	0.80563400
C	-1.07296700	-0.11088300	1.11513500
C	0.04156200	0.19207200	0.15827300
C	-0.11701200	1.61984800	-0.41504900
C	-1.55108900	1.93806800	-0.86076500
C	-2.57092500	1.70207500	0.26000300
H	-3.11244400	0.15065700	1.69568400
H	0.19795100	2.31674800	0.37008000
H	0.57516900	1.74613100	-1.25154300
H	-1.60044200	2.97983400	-1.19268000
H	-1.80988500	1.31352200	-1.71792500
H	-2.39036700	2.39265900	1.09156200
H	-3.58935200	1.88073900	-0.09590800
C	1.41014700	-0.06056400	0.81387100
H	1.42383000	-1.10619500	1.13852800
H	1.46183400	0.54709600	1.72505500
O	-0.08756100	-0.61918500	-1.05729900
O	-0.20152700	-2.02659200	-0.65793300
H	-0.86053900	-0.71620200	1.98810500
H	0.24701500	-2.43566400	-1.40977300
O	-3.05403000	-0.53951600	-0.25223000
O	-2.98304600	-1.93141800	0.13954800
H	-2.11804700	-2.17483400	-0.23749100
C	2.63527400	0.21191800	-0.06488600
H	2.69342800	1.27753300	-0.31404900
H	2.52201900	-0.31985700	-1.01592800
C	3.94880600	-0.21277300	0.60331700
H	4.05405000	0.31006900	1.56175600
H	3.90121900	-1.28137200	0.84539400
C	5.18116800	0.05996000	-0.26356400
H	5.27851000	1.12653300	-0.48898100
H	6.09978700	-0.25835800	0.23661300
H	5.11965800	-0.47403500	-1.21700000

TS39

C	2.26896100	0.43580900	-0.90011300
C	0.84659500	0.15869900	-1.26716300
C	-0.21590600	0.31724700	-0.36941900
C	-0.00828900	1.16268300	0.87311400
C	1.45480800	1.31968800	1.31812200
C	2.40332700	1.54617300	0.13922800
H	2.85537100	0.67237400	-1.79555500
H	-0.43581900	2.15073800	0.64994400
H	-0.60180400	0.74850600	1.69014800
H	1.52943400	2.15297800	2.02307200

H	1.76076100	0.42277200	1.85627800
H	2.17752200	2.49559900	-0.36169100
H	3.44140200	1.59765200	0.47430700
C	-1.62286300	0.26325200	-0.94525600
H	-1.65897600	-0.56831100	-1.65740800
H	-1.76804500	1.17783500	-1.53650500
O	-0.20357300	-1.49142600	0.24489800
O	0.62119300	-1.68327100	1.35251100
H	0.65845900	-0.33098400	-2.21644900
H	1.48456600	-1.86397200	0.94019700
O	2.76915900	-0.84526100	-0.40102500
O	4.19925100	-0.72419500	-0.14812800
H	4.54928000	-1.22729000	-0.89667600
C	-2.77386700	0.12352100	0.05802800
H	-2.80582200	0.99819500	0.71769800
H	-2.58577300	-0.74598300	0.69328900
C	-4.13477300	-0.02440800	-0.63295500
H	-4.11283200	-0.90515800	-1.28596000
H	-4.30774300	0.83723000	-1.29003500
C	-5.29752700	-0.15281600	0.35501600
H	-5.37109600	0.73078500	0.99684400
H	-6.25294800	-0.26587300	-0.16462200
H	-5.16697100	-1.02295400	1.00546800

P39

C	-1.87871800	-0.62386800	0.56487100
C	-0.40555300	-0.43191400	0.32847200
C	0.15287800	0.74247900	0.01426500
C	-0.68141900	1.99565700	-0.15347600
C	-2.17472200	1.70589200	-0.33423500
C	-2.64798100	0.68911700	0.70552600
H	-2.03554800	-1.25247100	1.44882000
H	-0.52702800	2.63877300	0.72532500
H	-0.29934500	2.56903000	-1.00608300
H	-2.75210000	2.63248100	-0.26594500
H	-2.34323900	1.29365200	-1.33478400
H	-2.48570600	1.08294500	1.71623200
H	-3.71677100	0.48005900	0.61565000
C	1.63940100	0.93747400	-0.18306200
H	1.79626900	1.30532600	-1.20710200
H	1.95840100	1.76812200	0.46343500
H	0.20010300	-1.32602600	0.41926500
O	-2.32426400	-1.39175200	-0.57625000
O	-3.63018900	-1.95419000	-0.23587300
H	-4.15959000	-1.59733500	-0.96078600

C	2.55097300	-0.26726500	0.06151500
H	2.40750500	-0.63535600	1.08427700
H	2.26308200	-1.08835700	-0.60515500
C	4.03418200	0.05646200	-0.15436500
H	4.32587100	0.87856400	0.51052800
H	4.17895700	0.42659800	-1.17659300
C	4.95341400	-1.14399900	0.08708300
H	4.70932400	-1.97088400	-0.58732500
H	6.00281800	-0.88238400	-0.07470300
H	4.85789700	-1.51540900	1.11237000

R40

C	2.14881600	-2.14066700	-0.44669800
C	0.81263500	-1.42574200	-0.68419900
C	2.88710100	-0.18270700	0.94525500
C	3.27752900	-1.14954800	-0.11933300
H	0.88350000	-0.80819400	-1.58263800
H	0.03559300	-2.17106100	-0.87316100
H	2.03656400	-2.84614800	0.38571000
H	2.41622200	-2.73291700	-1.32676500
H	0.84528600	1.41408500	-1.42004600
H	3.65732200	0.31629200	1.52379000
H	3.52509700	-0.58208500	-1.03266500
H	4.19050600	-1.68325900	0.16430400
C	-0.96986100	0.12523100	0.39507200
H	-1.17461600	0.68454600	1.31214700
O	1.50430100	1.71454100	0.31388800
O	1.76027300	1.54852500	-1.10529000
C	0.40612900	-0.56360800	0.52997200
H	0.29325400	-1.25624400	1.37658400
C	1.52240500	0.40729300	0.99187200
H	1.29228900	0.74928800	2.00861300
O	-0.95690200	1.11077500	-0.67632400
O	-1.10397400	2.44089800	-0.12095800
H	-0.18574300	2.60292600	0.17506500
C	-2.13342700	-0.83104400	0.12120100
H	-2.05068800	-1.69165600	0.79674100
H	-2.03876900	-1.22166100	-0.89740700
C	-3.50511900	-0.16739800	0.28458500
H	-3.53856900	0.72823700	-0.34198200
H	-3.61955700	0.17545700	1.31986500
C	-4.66371500	-1.10017900	-0.07774400
H	-4.66583000	-1.99812800	0.54864600
H	-5.62803600	-0.60252400	0.05368800

H	-4.59836800	-1.42465500	-1.12091300

TS40

C	-1.79359700	-2.42586200	0.56566700
C	-0.61343100	-1.48770500	0.85004800
C	-2.71302500	-0.56753300	-0.88639900
C	-3.03578400	-1.65745700	0.08730400
H	-0.85780100	-0.79479500	1.65977400
H	0.24854100	-2.07072400	1.18342600
H	-1.49895300	-3.14804600	-0.20501900
H	-2.04093800	-3.00704100	1.45855700
H	-3.36411100	2.47414600	-0.09346100
H	-3.53045200	-0.13065700	-1.45247800
H	-3.54113100	-1.19915100	0.95225500
H	-3.77306500	-2.34151300	-0.34799500
C	1.03672000	0.18573100	-0.24950000
H	1.08389100	0.91519200	-1.06433500
O	-1.84039800	1.47444800	0.33374500
O	-2.44374400	2.52436900	-0.38717800
C	-0.24520000	-0.67545600	-0.40405600
H	0.00405300	-1.39466100	-1.20106300
C	-1.46543400	0.04031600	-0.95145800
H	-1.29083100	0.75414000	-1.74974900
O	1.05602000	0.92330700	0.98018100
O	0.69845800	2.30122200	0.73673000
H	-0.27800500	2.21912500	0.70080500
C	2.31597200	-0.66346300	-0.25974900
H	2.27284600	-1.36036400	-1.10567500
H	2.34440800	-1.27451200	0.64956200
C	3.59356700	0.17787900	-0.34412300
H	3.58010700	0.91262900	0.46490700
H	3.58801900	0.74814000	-1.28087000
C	4.86874200	-0.66559800	-0.26538500
H	4.91565600	-1.39936500	-1.07694100
H	5.76248600	-0.03957500	-0.33361000
H	4.92139300	-1.21484300	0.67998300

P40

C	2.73230400	-1.49841500	-0.43014100
C	1.25330100	-1.12784000	-0.59444300
C	0.74900300	-0.30522900	0.60794700
C	1.75075900	0.76126000	0.99439800
C	3.01360200	0.79424100	0.57125600
C	3.61332900	-0.24521200	-0.33984600
H	0.65073200	-2.03255900	-0.71191600
H	3.05836900	-2.13098200	-1.26124800

H	3.66095200	1.60696700	0.89128000
H	4.61684100	-0.51046700	0.01237500
C	-0.65156000	0.31219300	0.39037000
H	1.39481100	1.53834600	1.66787300
H	-0.87438000	0.96424300	1.24290300
H	0.63179300	-0.98330600	1.46700000
H	1.12549300	-0.53651900	-1.50669200
H	2.85644800	-2.09121600	0.48425600
H	3.75718900	0.18887300	-1.33926900
O	-0.53294800	1.15871000	-0.77013000
O	-1.45875100	2.27218900	-0.63867800
H	-0.82762400	3.00385800	-0.59973900
C	-1.77034700	-0.71987800	0.22395200
H	-1.69278400	-1.44060300	1.04883000
H	-1.59414200	-1.28059400	-0.70015900
C	-3.18809500	-0.13525000	0.19929500
H	-3.26864700	0.58474300	-0.61834300
H	-3.36208600	0.43180500	1.12155800
C	-4.26411200	-1.21479000	0.05051100
H	-4.22675800	-1.93349800	0.87580100
H	-5.26609600	-0.77735100	0.03701400
H	-4.13563500	-1.77541200	-0.88086100

R41

C	0.99862300	-1.81141800	-0.22638900
C	2.49373500	-1.49188300	-0.09718000
C	0.63944400	0.53746700	-0.97570900
C	0.08825300	-0.56797100	-0.12770400
H	2.71014700	-1.13404200	0.91257400
H	3.07500800	-2.40742300	-0.24316600
H	0.80472500	-2.28436800	-1.19589200
H	0.68414300	-2.52747500	0.53787800
H	1.01242600	1.96415900	1.23395800
H	-0.03540200	1.14871600	-1.56018300
O	2.54293900	1.39926300	0.30762000
O	1.65360000	2.46919500	0.70550000
C	2.94329900	-0.42351400	-1.10310100
H	2.83578000	-0.79234600	-2.12910500
C	2.10128400	0.85397600	-0.95939000
H	2.33786900	1.57702700	-1.74712700
H	3.99673900	-0.16796700	-0.95770100
C	-1.37098200	-0.91900600	-0.46246600
H	-1.40695100	-1.20125200	-1.52153300
O	0.16663400	0.01132200	1.22502200

O	-0.34689500	-0.93587400	2.20750500
H	0.47055500	-1.16563700	2.67031200
C	-2.39698600	0.18301600	-0.17721000
H	-2.13490600	1.09881500	-0.72041400
H	-2.36028000	0.43693800	0.88595600
H	-1.64117600	-1.81266900	0.10833900
C	-3.82463400	-0.22912900	-0.55667200
H	-4.09036100	-1.14558900	-0.01612500
H	-3.86009700	-0.48418100	-1.62312600
C	-4.86215300	0.85661700	-0.25775600
H	-4.64453200	1.77544300	-0.81132000
H	-5.86951900	0.53351300	-0.53417500
H	-4.87368300	1.10816500	0.80713400

TS41

C	-0.07729700	1.69607000	-0.25680400
C	1.31847900	2.05236300	-0.78815800
C	0.98479700	-0.15412100	1.04871300
C	-0.16735400	0.21198900	0.14571700
H	1.50059000	1.48051200	-1.70017300
H	1.35364100	3.11136000	-1.05981700
H	-0.32811500	2.30484700	0.61969700
H	-0.83035200	1.90355400	-1.02040800
H	3.55804700	-2.44881400	0.33811100
H	0.84940400	-0.99276600	1.72279600
O	2.99237200	-0.80042000	-0.37040400
O	3.96728300	-1.57305000	0.27942900
C	2.42111700	1.74632300	0.23249800
H	2.41333100	2.50312000	1.03012900
C	2.24817900	0.41139400	0.90898500
H	2.99552700	0.15889700	1.65345800
C	-1.49924000	-0.19805800	0.79295700
H	-1.44525200	-1.27912400	0.96376100
H	-1.56365300	0.27286500	1.78231400
H	3.41039900	1.79211900	-0.22834700
O	-0.01997200	-0.45715700	-1.14745400
O	0.33997100	-1.84697300	-0.95845800
H	1.31130100	-1.75206200	-0.94607100
C	-2.75602400	0.11897900	-0.02357000
H	-2.88758900	1.20394600	-0.11002900
H	-2.62102600	-0.26282800	-1.04048700
C	-4.02599400	-0.48613000	0.58700400
H	-4.14676800	-0.12120000	1.61467400
H	-3.90469900	-1.57326400	0.66279800
C	-5.28938400	-0.16915600	-0.21793700

H	-6.17616100	-0.61938200	0.23631600
H	-5.45956900	0.91027800	-0.27917900
H	-5.20996400	-0.54918900	-1.24088600

P41

C	-2.37449600	-1.20292200	-0.97837800
C	-0.89640600	-1.16803900	-0.57589500
C	-0.49866700	0.17226800	0.06581900
C	-1.56692200	0.65729700	1.02616900
C	-2.78937900	0.13144600	1.11165400
C	-3.28428700	-0.98735200	0.23579700
H	-0.25958800	-1.34409000	-1.44569300
H	-2.60926000	-2.15374400	-1.46558300
H	-3.47801900	0.51295700	1.86215700
H	-4.31071300	-0.77743500	-0.08537300
C	0.86418600	0.12243700	0.78104600
H	-1.27849400	1.46294300	1.69443000
H	1.06861200	1.13001400	1.15677500
H	0.76098500	-0.52473300	1.66054100
H	-0.68408100	-1.96409500	0.14689300
H	-2.55578100	-0.41264500	-1.71257300
H	-3.34764700	-1.90699900	0.83561400
O	-0.41309000	1.05518400	-1.09545300
O	-0.15743300	2.41385200	-0.65641200
H	-1.05125000	2.78269700	-0.68714000
C	2.04481200	-0.33997000	-0.07968600
H	1.90394400	-1.38261300	-0.38794700
H	2.06884300	0.25612900	-0.99743600
C	3.38952700	-0.21613300	0.64698500
H	3.54082700	0.82769400	0.94681300
H	3.35696400	-0.79781400	1.57671400
C	4.57671400	-0.67930500	-0.20209200
H	5.52123500	-0.57277400	0.33861400
H	4.47389400	-1.73139900	-0.48634800
H	4.65322900	-0.09474500	-1.12405600

R42

C	0.01430300	1.79928800	0.00470900
C	0.08613700	0.27926100	0.23068200
C	-2.51862600	1.59418600	0.03865600
C	-1.33396800	2.23508100	-0.59904900
H	0.17388100	2.28888700	0.97125700
H	0.82376300	2.11593900	-0.65539700
H	-4.20667300	-1.98128700	0.09014300
H	-3.50164600	2.02299200	-0.12539200

H	-1.33123400	1.97929900	-1.67012300
H	-1.42951000	3.32610600	-0.55707300
O	-2.91612900	-0.68680800	-0.39818900
O	-3.25292300	-1.96111000	0.24809100
C	-1.06841700	-0.15125400	1.15595900
H	-0.91231100	0.32241600	2.13088600
C	-2.45615100	0.23982700	0.64896900
H	-3.18951700	0.14689400	1.45762600
C	1.42867200	-0.20161200	0.80903200
H	1.53424000	0.19604200	1.82609900
H	1.35881800	-1.29005600	0.90437700
H	-1.04112600	-1.22929200	1.32049700
O	-0.09352100	-0.23283100	-1.11097400
O	-0.29530800	-1.66551000	-1.08092000
H	-1.26807200	-1.68872500	-1.09450400
C	2.67014100	0.14505100	-0.02011300
H	2.83129300	1.22961600	-0.02979300
H	2.49556600	-0.15467500	-1.05822200
C	3.94005400	-0.53668700	0.50356500
H	4.10077200	-0.25444800	1.55167900
H	3.79074600	-1.62288500	0.50075800
C	5.18835200	-0.19190000	-0.31351200
H	5.38704200	0.88452000	-0.29851600
H	6.07520400	-0.69813600	0.07770300
H	5.06882000	-0.49059100	-1.35945400

TS42

C	0.06435600	1.79353200	-0.13537800
C	0.13828000	0.30590300	0.25196400
C	-2.46004700	1.60713600	0.01362300
C	-1.30028100	2.17390400	-0.73731100
H	0.25590900	2.38473200	0.76640200
H	0.85479400	2.02917100	-0.85005300
H	-4.63498900	-1.51158600	-0.24093700
H	-3.44326700	2.03262900	-0.16099000
H	-1.35143700	1.80646700	-1.77224100
H	-1.39451500	3.26366500	-0.80425000
O	-2.87538300	-0.91526700	-0.46854700
O	-3.79743000	-1.76474600	0.17296500
C	-0.99631200	-0.02203200	1.24167900
H	-0.76199800	0.45404000	2.20230000
C	-2.35100200	0.46271900	0.79390300
H	-3.18368900	0.20278700	1.43752400
H	-1.02678200	-1.09567600	1.42975900
C	1.49395800	-0.11515300	0.84644700

H	1.41783600	-1.18071900	1.08592100
H	1.63816600	0.41182800	1.79788100
O	-0.07974800	-0.34815400	-1.01823600
O	-0.26403800	-1.77156500	-0.82859100
H	-1.24190400	-1.79018600	-0.78082600
C	2.70915800	0.10208100	-0.06176800
H	2.87644300	1.17352500	-0.22458800
H	2.49792300	-0.33421300	-1.04286500
C	3.99170300	-0.51524500	0.50894300
H	3.83558600	-1.58994000	0.65982800
H	4.18997600	-0.09411600	1.50262900
C	5.21344200	-0.29914800	-0.38851300
H	5.41925200	0.76686100	-0.52780600
H	6.10982000	-0.75673700	0.03894900
H	5.05614400	-0.73685500	-1.37889800

P42

C	1.56960700	-1.38775300	-0.42333100
C	0.52279100	-0.28950000	-0.14557900
C	0.84820400	0.41449700	1.18096800
C	2.31839900	0.68242200	1.38173900
C	3.28028000	0.12781400	0.64584300
C	3.00442200	-0.84503600	-0.46824600
H	1.32233600	-1.89210300	-1.36156700
H	4.31893900	0.38370400	0.83842200
H	3.71260600	-1.67989100	-0.41486200
H	2.58260400	1.37141000	2.18002900
H	0.46719100	-0.20196000	2.00565700
H	1.47968400	-2.12977200	0.37640700
H	3.18871600	-0.35513900	-1.43130700
H	0.28668400	1.35011500	1.23205000
C	-0.89418800	-0.89091100	-0.18426800
H	-1.03614600	-1.30222300	-1.18994800
H	-0.90845900	-1.74551900	0.50489200
O	0.74038900	0.59218900	-1.28142100
O	0.04650200	1.85759200	-1.11619000
H	0.80162100	2.44804600	-0.98820900
C	-2.07349900	0.03263700	0.15072600
H	-1.95734500	0.43980100	1.16215500
H	-2.07365500	0.88891400	-0.52750700
C	-3.42156400	-0.69399000	0.06293700
H	-3.54434600	-1.10423100	-0.94679500
H	-3.41723400	-1.55598800	0.74193800
C	-4.61369300	0.20866400	0.39302800
H	-4.66351000	1.06124200	-0.29120800

H	-5.56003500	-0.33413000	0.31765000
H	-4.53933700	0.60641100	1.41018700

R43

C	-0.53814700	-0.22319500	0.57061900
C	0.65830900	0.71199500	0.82688600
C	1.15037400	-2.01627400	-0.06723500
C	-0.14246600	-1.35497500	-0.41044900
H	-0.75801300	-0.68654200	1.54222300
H	4.65774000	-0.13752300	-1.17339500
H	1.32093400	-3.04873100	-0.35008700
H	-0.06378700	-0.91629900	-1.41853500
H	-0.93729500	-2.10365500	-0.46398500
O	2.88348500	-0.71308700	-0.85443500
O	4.01395200	0.18233800	-0.52770900
C	1.88598400	-0.05973500	1.32163800
H	1.64661300	-0.49368900	2.29867100
C	2.31167000	-1.20100500	0.39208600
H	3.06999100	-1.82240000	0.88234800
H	2.72103400	0.62288200	1.47803200
H	0.38227800	1.46294000	1.57905900
C	-1.78912600	0.55243100	0.12272400
H	-1.92843100	1.39723300	0.80875000
H	-1.60550200	0.99263400	-0.86219500
O	0.88928000	1.38160700	-0.41804800
O	1.99692000	2.30001500	-0.26315200
H	2.73691900	1.75329500	-0.58318800
C	-3.08022100	-0.27482500	0.09379300
H	-2.99378100	-1.08581900	-0.63890600
H	-3.22663900	-0.75841000	1.06925600
C	-4.32064000	0.56155900	-0.24307800
H	-4.42246800	1.36706200	0.49406600
H	-4.16936400	1.05432300	-1.21085000
C	-5.61236600	-0.25962200	-0.28396600
H	-5.80776300	-0.73884500	0.68066500
H	-6.47624200	0.36628400	-0.52396100
H	-5.55554300	-1.04985600	-1.03930800

TS43

C	-0.61410100	-0.13596200	0.61476400
C	0.54550200	0.85409500	0.83152700
C	1.20487500	-1.80816100	-0.01837800
C	-0.16041600	-1.27796000	-0.32255000
H	-0.82237300	-0.57147800	1.60154000
H	4.71248100	-1.17298800	-1.27845500

H	1.48285400	-2.76717600	-0.44468600
H	-0.16479100	-0.91199400	-1.36055000
H	-0.88170900	-2.10133500	-0.29625300
O	3.14286700	-0.26438200	-0.81710600
O	4.51633900	-0.49707500	-0.61395400
C	1.76391100	0.15111800	1.43863800
H	1.50851000	-0.15559900	2.46134900
C	2.17128600	-1.06767300	0.65221700
H	3.06751700	-1.57862500	0.98463600
H	2.59901800	0.84624400	1.52261300
C	-1.89205800	0.56896300	0.12847900
H	-2.09309700	1.41113100	0.80225200
H	-1.70493700	1.00797400	-0.85654200
H	0.23076100	1.67192900	1.49244700
O	0.80946500	1.38205400	-0.47248500
O	1.97267300	2.23987300	-0.42944200
H	2.65236400	1.58374900	-0.68377200
C	-3.13632100	-0.32594600	0.07467200
H	-2.98661900	-1.13825300	-0.64638800
H	-3.28325200	-0.80717100	1.05126500
C	-4.41000200	0.43902500	-0.30502700
H	-4.57552300	1.24488500	0.42004100
H	-4.25847900	0.92968500	-1.27379300
C	-5.65384400	-0.45141400	-0.37188000
H	-5.85004000	-0.93090000	0.59240200
H	-6.54302700	0.12437300	-0.64256900
H	-5.53339900	-1.24472500	-1.11640300

P43

C	1.38797400	0.57947800	0.60321000
C	0.14317600	-0.30109500	0.39446700
C	0.40967400	-1.34988000	-0.70370000
C	1.76112400	-2.00257400	-0.57357300
C	2.74156200	-1.51876300	0.18696500
C	2.61504800	-0.25047700	0.99032500
H	1.18669300	1.33590900	1.37027300
H	3.68944700	-2.04743900	0.24381600
H	3.50687600	0.37317400	0.87468000
H	1.92506200	-2.91368500	-1.14320300
H	0.32524200	-0.87929600	-1.69231400
H	0.01594500	-0.84150500	1.34260100
H	2.56218900	-0.48517400	2.06219200
H	-0.36713700	-2.12075000	-0.67193400
C	-1.12287000	0.53710200	0.14896300
H	-1.18503400	1.30306400	0.93238900

H	-1.01565800	1.07941600	-0.79584900
O	1.57325600	1.26287400	-0.64643600
O	2.57900500	2.29432800	-0.44975400
H	3.24365000	2.00677500	-1.08986500
C	-2.42958900	-0.26558500	0.14270800
H	-2.42058800	-0.99334700	-0.67703300
H	-2.50381100	-0.85040800	1.06962400
C	-3.67602700	0.61691600	0.00446300
H	-3.69935600	1.33935100	0.82926400
H	-3.59622600	1.20947800	-0.91477800
C	-4.98350000	-0.17988100	-0.01332600
H	-5.10743000	-0.75787300	0.90793100
H	-5.85130200	0.47811600	-0.11097700
H	-5.00607500	-0.88486900	-0.85031800

R44

C	0.49565100	-0.23427400	0.54640000
C	0.05203300	-1.31972100	-0.45446600
C	-1.90722700	-0.07104000	1.25849500
C	-0.69066200	0.70588600	0.87829400
H	-0.18820100	-0.84814300	-1.41058600
H	0.73167200	-0.72643700	1.50052900
H	-3.45915000	1.00522300	-0.62547600
H	-2.50066800	0.23948400	2.10853500
H	-0.41040500	1.40526900	1.67407100
O	-2.85358900	-0.76432500	-0.88367400
O	-3.94446100	0.16079800	-0.65761500
C	-1.16492700	-2.11670500	0.03911300
H	-0.90080100	-2.69418300	0.93206800
C	-2.35191500	-1.20986000	0.39858800
H	-3.14437200	-1.77982100	0.89480300
H	-1.49112200	-2.83071000	-0.72261800
H	0.87969300	-2.01128200	-0.63381800
C	1.74442100	0.54967400	0.10110400
H	1.87193900	1.41005600	0.77039000
H	1.57469100	0.96548300	-0.89734200
O	-0.88657600	1.47509100	-0.33539800
O	-2.05925000	2.32679100	-0.11887600
H	-1.80262100	3.08191700	-0.66511400
C	3.04090800	-0.27014200	0.10704700
H	2.95828800	-1.11132900	-0.59058300
H	3.18749600	-0.71108700	1.10210100
C	4.27718100	0.55850400	-0.26239700
H	4.37087100	1.39779700	0.43739900

H	4.12804800	1.00465000	-1.25297200
C	5.57410200	-0.25556700	-0.25930500
H	5.52529700	-1.08055800	-0.97686400
H	6.43502200	0.36394400	-0.52477100
H	5.76794100	-0.68762500	0.72750700

TS44

C	0.59533500	-0.14672400	0.55573000
C	0.08623600	-1.17995000	-0.46872700
C	-1.79518700	0.15406100	1.28662400
C	-0.53785800	0.85565800	0.85749900
H	-0.14008700	-0.65948700	-1.40237000
H	0.79792300	-0.66294400	1.50520600
H	-4.89482900	0.28302500	-0.38542600
H	-2.47713200	0.67266000	1.94979000
H	-0.22860600	1.57753100	1.62265000
O	-3.16430400	-0.32901000	-0.79614700
O	-4.52588200	-0.59063400	-0.58159200
C	-1.16503800	-1.92487900	0.02022400
H	-0.87793800	-2.67847700	0.76748500
C	-2.18542300	-1.03862100	0.68637200
H	-3.05947300	-1.54303500	1.08379500
H	-1.64117500	-2.46971400	-0.79836700
C	1.88762800	0.56914500	0.12167600
H	1.73175800	1.03265200	-0.85744600
H	2.07489800	1.39483600	0.81973500
H	0.86830200	-1.91062900	-0.68877500
O	-0.72832200	1.56482600	-0.39203500
O	-1.97116800	2.30495900	-0.35505900
H	-2.56624700	1.61887700	-0.71133100
C	3.12961500	-0.32979400	0.08311300
H	2.99070600	-1.13555300	-0.64698400
H	3.25569700	-0.81918400	1.05835300
C	4.41278300	0.43430000	-0.26515400
H	4.56444900	1.23643000	0.46713800
H	4.28370100	0.92977700	-1.23472300
C	5.65586600	-0.45868100	-0.30902300
H	5.54968500	-1.24855200	-1.05929300
H	6.55195200	0.11626300	-0.55798500
H	5.83008500	-0.94269300	0.65721200

P44

C	-0.50110900	1.34108700	0.76944900
C	-0.17809700	0.30446700	-0.32066600
C	-1.41209900	-0.58250800	-0.56865000
C	-2.68458100	0.21951400	-0.69758000

C	-2.78757500	1.48606600	-0.29386700
C	-1.66051300	2.25209400	0.34384000
H	0.37917500	1.94701500	0.99793400
H	-3.72598200	2.01700500	-0.43684000
H	-2.04031800	2.81438300	1.20458800
H	-3.53489400	-0.28139400	-1.14889300
H	-0.02246100	0.84483000	-1.26534200
H	-0.77188700	0.80860700	1.68703800
H	-1.30364600	3.01238700	-0.36611200
H	-1.25687700	-1.18334900	-1.47280700
C	1.08741600	-0.52553600	-0.03970400
H	1.10824400	-1.37498500	-0.73320000
H	1.02100800	-0.96197400	0.96186600
O	-1.47110100	-1.50179500	0.54516800
O	-2.50111000	-2.48220200	0.25822800
H	-3.20530700	-2.17885100	0.84784800
C	2.39785900	0.25848900	-0.18230000
H	2.41184000	1.10355800	0.51629700
H	2.45285900	0.69451800	-1.18882500
C	3.64355100	-0.60210900	0.06049500
H	3.63962700	-1.44676300	-0.63892100
H	3.58764600	-1.04019600	1.06416700
C	4.95469700	0.17533000	-0.08526700
H	5.00437200	1.00502400	0.62700700
H	5.82187300	-0.46645400	0.09281600
H	5.05513000	0.59724500	-1.09029100
